# New insights into the evolutionary dynamic and lineage divergence of gasdermin E in metazoa

**DOI:** 10.3389/fcell.2022.952015

**Published:** 2022-07-22

**Authors:** Zihao Yuan, Shuai Jiang, Kunpeng Qin, Li Sun

**Affiliations:** ^1^ CAS and Shandong Province Key Laboratory of Experimental Marine Biology, Institute of Oceanology, CAS Center for Ocean Mega-Science, Chinese Academy of Sciences, Qingdao, China; ^2^ Laboratory for Marine Biology and Biotechnology, Pilot National Laboratory for Marine Science and Technology, Qingdao, China; ^3^ College of Earth and Planetary Sciences, University of Chinese Academy of Sciences, Beijing, China

**Keywords:** metazoan, fish, gasdermin, evolution, recognition motif, auto-inhibition

## Abstract

Gasdermin (GSDM) is a family of pore-forming proteins that induce pyroptosis. To date, the origin and evolution of GSDM in Metazoa remain elusive. Here, we found that GSDM emerged early in Placozoa but is absent in a large number of invertebrates. In the lower vertebrate, fish, three types of GSDME, i.e., GSDMEa, GSDMEb, and a previously unreported type (designated GSDMEc), were idenitied. Evolutionarily, the three GSDMEs are distinctly separated: GSDMEa is closely related to tetrapod GSDME; GSDMEb exists exclusively in fish; GSDMEc forms the lineage root of tetrapod GSDMA/B/C/D. GSDMEc shares conserved genomic features with and is probably the prototype of GSDMA, which we found existing in all tetrapod classes. GSDMEc displays fast evolutionary dynamics, likely as a result of genomic transposition. A cross-metazoan analysis of GSDME revealed that GSDMEa shares a conserved caspase recognition motif with the GSDME of tetrapods and cnidarians, whereas GSDMEb has a unique caspase recognition motif similar to that of mammalian GSDMD, and GSDMEc exhibits no apparent caspase recognition motif. Through functional test, four highly conserved residues in vertebrate GSDME proved to be essential to auto-inhibition. Together our results provide new insights into the origin, evolution, and function of metazoan GSDMs.

## Introduction

Pyroptosis is an inflammatory type of programmed cell death executed by unique pore-forming proteins belonging to the gasdermin (GSDM) family (Shi et al., 2017; Kesavardhana et al., 2020; Liu et al., 2021a). In humans, six GSDM family members have been identified, five of which are designated GSDMA to GSDME (DFNA5), and the sixth is a truncate GSDM named GSDMF (DFNB59 or PJVK) (Tamura et al., 2007; Zhang et al., 2020). GSDMA-E exhibit similar structural architectures containing a pore-forming N-terminal (NT) domain and an auto-inhibitory C-terminal (CT) domain connected by a linker region (Shi et al., 2015). Different from GSDMA-E, GSDMF is composed of a conserved NT domain and a very short CT domain. GSDMF exhibits no pyroptosis-inducing capacity, but was reported to be involved in peroxisome homeostasis (Defourny et al., 2019; Angosto-Bazarra et al., 2022). During pyroptosis, GSDM is cleaved by a protease, and the cleavage releases the NT fragment that induces cell death by forming pores on the plasma membrane (Ding et al., 2016; Evavold et al., 2018; Liu et al., 2020). Caspase is the most well studied protease involved in GSDM-mediated pyroptosis. In human, caspase-1/4/5/8 and neutrophil elastase cleave GSDMD, while caspase-3 cleaves GSDME, and all these cleavages occur in the inter-domain linker region (Kayagaki et al., 2015; Shi et al., 2015; Rogers et al., 2017; Wang et al., 2017; Kambara et al., 2018; Orning et al., 2018). In contrast, different cleavage modes exist in the lower vertebrate teleost, which comprise more than half of all living vertebrate species and possess only GSDME and GSDMF (Hofmann, 2020; Morimoto et al., 2021). In zebrafish *Danio rerio*, two GSDME orthologs, designated GSDMEa and GSDMEb, are cleaved by caspase-3 and caspy2 (caspase-4/5 homolog), respectively, to induce pyroptosis (Li et al., 2020; Wang et al., 2020), while in tongue sole *Cynoglossus semilaevis,* GSDME-mediated pyroptosis is activated by caspase-1/3/7 (Jiang et al., 2019). A recent study showed that in turbot *Scophthalmus maximus*, GSDMEa is cleaved by caspase-3/7 and caspase-6, which activate and inactivate, respectively, GSDMEa (Xu et al., 2022).

Phylogenetically, GSDME and GSDMF are categorized into a segregated GSDM cluster far from GSDMA-D, and evolutionary analysis supports the notion that GSDME/F represent the ancient members of vertebrate GSDM (Zhang and Lieberman, 2020; Daskalov and Glass, 2021). Recently, GSDME homologs were identified in the invertebrate *Orbicella faveolata* (Cnidaria, Anthozoa), Mollusca, Brachiopoda, and Cephalochordata (Jiang et al., 2020). *O. faveolata* GSDME was activated by caspase-3 and involved in pathogen-triggered coral necrotic death. Later, GSDM homologs were also identified in sea urchin and acorn worm by homologous sequence searching (De Schutter et al., 2021). These findings suggest that GSDM may have an extensive presence in invertebrate. However, the origin and evolution of GSDM through metazoan remain elusive.

Next to GSDME/F, GSDMA is also considered as an early form of GSDM that has been found to exist in Mammalia, Aves and Reptilia, but not in amphibians or Actinopterygii (De Schutter et al., 2021; Angosto-Bazarra et al., 2022). The generally accepted view is that gene duplication of GSDMA has resulted in the emergence of GSDMB/C/D in Mammalia (Angosto-Bazarra et al., 2022). Human has one copy of GSDMA locating together with GSDMB on chromosome 17, while mouse has three GSDMA paralogs locating tandemly on chromosome 11 (Zheng et al., 2020; Deng et al., 2022). The high variation of GSDMA in vertebrates raises the question about strong selective forces that may have driven gene amplification during evolution (De Schutter et al., 2021). However, the evolutionary path of GSDMA and its relationship with the ancient GSDME/F are unclear.

In the present study, by deep data mining, we investigated the primitive forms of GSDM through the entire metazoan phylogeny and explored their evolutionary fates and consequences. We also examined the conserved determinants essential to GSDME auto-inhibition and proteolytic activation. Our work provides new understandings of GSDM from the evolutionary and functional aspects.

## Materials and methods

### Sequence collection

A total of 284 GSDME reference sequences and 367 GSDMF reference sequences were downloaded from NCBI Orthologs. The sequences were used as queries to search against the non-redundant database and specialized databases such as OIST Marine Genomics Unit (https://marinegenomics.oist.jp/) (Luo et al., 2018) via TBLASTN with E-value set as 1e-5 to ensure accuracy. The sequences were next validated using the conserved domains database (https://www.ncbi.nlm.nih.gov/cdd/) (Lu et al., 2020), and filtered based on length (100–1,000 amino acids). The protein sequences of the putative GSDM homologs were further aligned using Clustal Omega (Madeira et al., 2019) to remove duplicates.

### Phylogenetic and syntenic analysis

The phylogenetic tree containing the major metazoan phyla was collected from the public knowledge-base TimeTree (http://timetree.org/) (Kumar et al., 2017). The phyla icon shown in the phylogenetic tree were downloaded from the PhyloPic (http://www.phylopic.org/), with the detailed credentials provided in the Supplementary Table S3. Phylogenetic analyses were based on the identified GSDME and GSDMF sequences. Multiple alignments of the sequences were conducted with Clustal Omega (Madeira et al., 2019). The phylogenetic tree was constructed using the maximum likelihood method with the IQTREE2 software (Minh et al., 2020) with the best substitution model. The bootstrap of 1,000 replications was conducted to evaluate the phylogenetic tree. The final presented tree was visualized with iTOL (https://itol.embl.de/) (Letunic and Bork, 2021). The phylogenetic distance was calculated with Mega7 (Kumar et al., 2016). The conserved syntenic blocks near the GSDME genes in common carp (*Cyprinus carpio*) were based on the information from Genomicus (https://www.genomicus.bio.ens.psl.eu/genomicus-100.01/) (Louis et al., 2012; Louis et al., 2015), NCBI database (http://www.ncbi.nlm.nih.gov/) and ENSEMBL genome browser (http://www.ensembl.org/).

### Repetitive element analysis

To explore the influence of the genome repetitive elements on the copy number variation of GSDME in Perciformes species. The *Poecilia formosa* (Amazon molly) genome generated by Wesley Warren and The Genome Institute, Washington University School of Medicine, was retrieved from NCBI Genome. The repetitive elements were identified using RepeatModeler 1.0.8 containing RECON (Bao and Eddy, 2002) and RepeatScout with default parameters (Price et al., 2005). The derived repetitive sequences were searched against Repbase (Bao et al., 2015). The total repetitive elements on the *P. formosa* scaffold 1,076 (Accession: NW_006801015.1) was subtotaled and collected in a 1,000 bp bin. The gene structure was plotted using R package “gggenes”, a ggplot2 extension (Hadley, 2016; R.Core.Team, 2018).

### The evolutionary analysis of GSDME in actinopterygii

The GSDME sequences were aligned via Clustal Omega (Madeira et al., 2019). Eleven sites with less than one mismatch in mammals, birds, and >80% consensus in teleost were considered conservative sites. These sites were visualized via Weblogo3 (Crooks et al., 2004). To compare the selection pressures in different clades, the GSDMEs from phyla Cnidaria and Mollusca were selected. The CDS sequences of the GSDMEs were first codon aligned *via* MUSCLE (Edgar, 2004). The Ka/Ks ratio of each gene was calculated using the KaKs calculator (Zhang et al., 2006) with the following setting: genetic code Table 1 (standard code); method of calculation: YN (Yang and Nielsen, 2000). The boxplot and violin plot were plotted *via* R package “ggplot2” and “plotly” (Hadley, 2016; R.Core.Team, 2018; Sievert, 2020). The pair-wise comparison of full-length GSDME from GSDMEc containing species (Supplementary Table S4) with 228 tetrapod GSDME and GSDMA was conducted *via* EMBOSS Needle (Madeira et al., 2022).

### Gene cloning and mutagenesis

The CDS of human GSDME was synthesized by Sangon Biotech (Shanghai, China). GSDME N-terminal (NT) and full length were sub-cloned from the synthesized sequence. Mutations of Phe2 to Ala (F2A), Ala5 to Asp (A5D), Gly17 to Asp (G17D), Trp44 to Ala (W44A), Gln47 to Ala (Q47A), Tyr51 to Ala (Y51A), Pro70 to Ala (P70A), Glu223 to Arg (E223R), Leu327 to Asp (L327D), Leu451 to Asp (L451D), Gly487 to Asp(G487D), and Leu491 to Asp (L491D) were performed using a Site-Directed Mutagenesis Kit (New England Biolabs, Beverly, MA, United States) according to the manufacturer’s instruction. The primers used for the mutagenesis are listed in the Supplementary Table S5. The CDS were subcloned into pmCherry-N1 or pCMV-c-Myc expression vector (Clontech, Mountain View, CA, United States).

### Confocal microscopy

The microscopic analysis of GSDME-induced pyroptosis was performed as described previously (Jiang et al., 2020). Briefly, HEK293T cells were plated on 35-mm culture dishes (Nest Scientific, Rahway, NJ, United States), and incubated at 37°C in 5% CO_2_ overnight. The cells were transfected with pmCherry-N1 or pmCherry-N1 expressing indicated GSDME variants using PlyJet transfection reagent (SignaGen Laboratories, Ijamsville, MD, United States). After 48 h transfection, Sytox green (Thermo Fisher Scientific, Oregon, United States) was used to stain the cells according to the manufacturer’s instructions. The images of the cells were recorded with a Carl Zeiss LSM 710 confocal microscope (Carl Zeiss, Jena, Germany).

### Lactate dehydrogenase assay

HEK293T cells were seeded in 96-well plates (Costar, Corning, NY, United States) and incubated at 37°C in 5% CO_2_ overnight. The cells were transfected with the indicated plasmid (0.1 μg per well) expressing GSDME variants using PlyJet transfection reagent (SignaGen Laboratories, Ijamsville, MD, United States). The medium was replaced by fresh Opti-modified Eagle’s medium (Opti-MEM; Thermo Fisher Scientific, Waltham, MA, United States) after 6 h, and the cells were incubated as above. After 48 h, the release of LDH from the transfected cells was measured by CytoTox-ONE homogeneous membrane integrity assay (Promega, Leiden, Netherlands).

### Statistical analysis

Statistical analysis of the differences in Ka/Ks ratio between different clades was performed with Wilcoxon rank test function in R (R.Core.Team, 2018). For experiments, the two-sample student *t*-test was used for comparison between groups with GraphPad Prism seven software (https://www.graphpad.com/), and statistical significance was defined as *p*-value < 0.05.

## Results

### GSDM emerged early in invertebrate metazoan and underwent varied evolutionary selections

**FIGURE 1:**
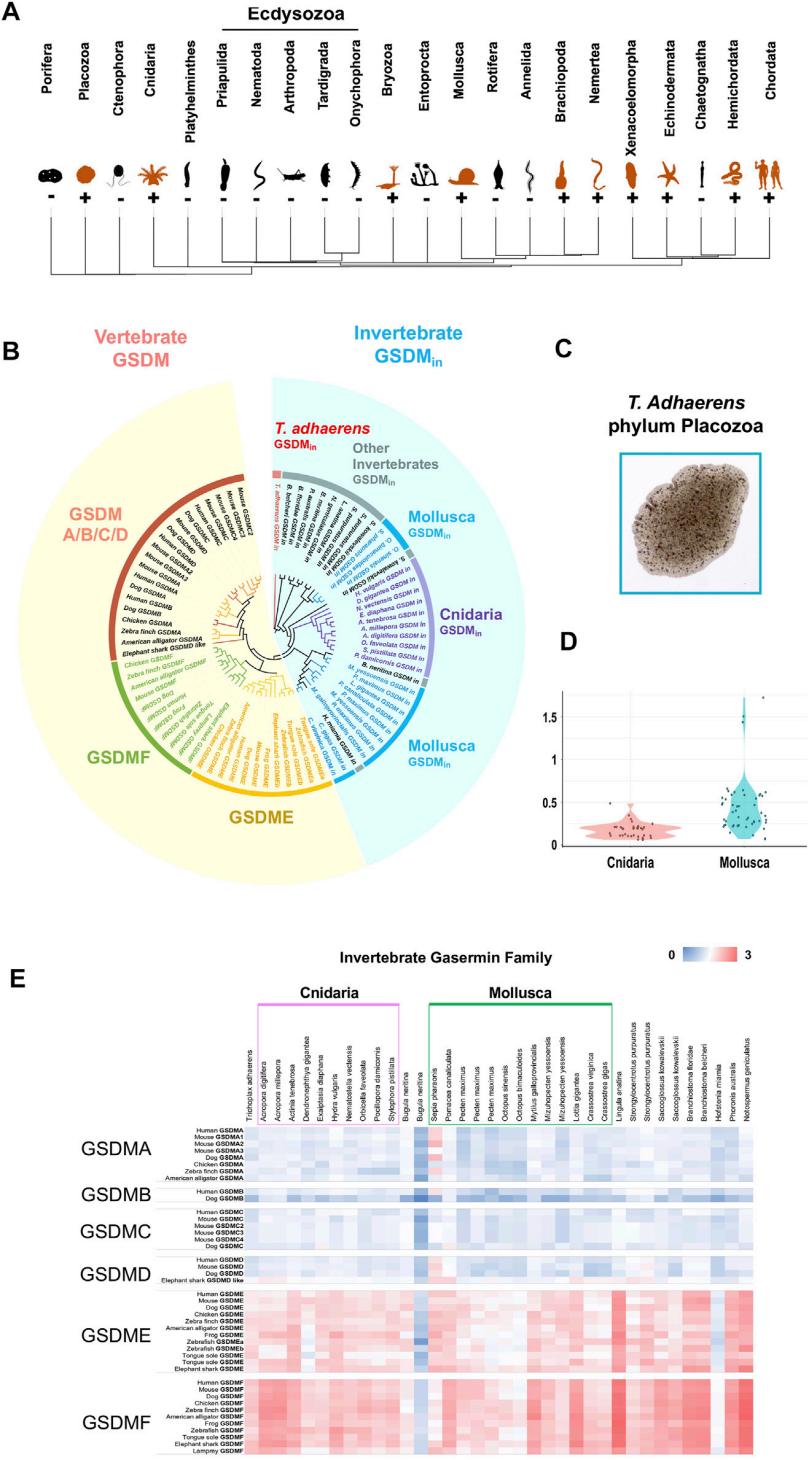
The identification and phylogenetic analysis of invertebrate GSDM. **(A)** The phylogenetic tree of the major metazoan phyla with GSDM distribution. Orange and black icons indicate GSDM-positive and negative phyla, respectively. **(B)** The phylogenetic analysis of GSDM in invertebrate and vertebrate. The different categories of GSDM and metazoan clades are presented in different colors. The vertebrate and invertebrate GSDM are shaded with yellow and blue, respectively. **(C)** A micrograph of *Trichoplax adhaerens* (phylum: Placozoa). **(D)** The violin plot showing the Ka/Ks ratios of GSDM_in_ in Cnidaria and Mollusca. **(E)** The genetic distance between invertebrate GSDM_in_ and vertebrate GSDMA/B/C/D/E/F. The color from blue to red indicates decreasing genetic distance.

To understand the origin and evolution of GSDM across the metazoan, we screened 34 phyla with available genomic sequences. We found that, besides Vertebrata and Cephalochordata, GSDM homologs also exist in 11 invertebrate clades (Figure 1A, Supplementary Table S1). The 23 invertebrate phyla that lack GSDM include Ecdysozoa, a major animal clade represented by fruit fly *Drosophila melanogaster* (Arthropoda) and nematode worm *Caenorhabditis elegans* (Nematoda). GSDM homologs in invertebrate share low identities (average 26.7%) with vertebrate GSDM and are phylogenetically segregated from the vertebrate GSDM clade. We therefore designated the invertebrate GSDM homologs GSDM_in_ (“_in_” for “invertebrate”). GSDM_in_ emerged as early as in Placozoa *Trichoplax adhaerens*, one of the most primitive multicellular metazoan (Figures 1A–C, Supplementary Figures S1–S11). GSDM_in_ is most abundant in Cnidaria and Mollusca, which formed two major clusters in the phylogenetic tree (Figure 1B). However, Cnidaria and Mollusca differ considerably in the sequence conservedness of GSDM_in_. The GSDM_in_ of Mollusca exhibit a much lower average identity (25.6%) than that of Cnidaria (54.5%) (Supplementary Figures S12, S13). Consistently, the pairwise Ka/Ks value of Mollusca GSDM_in_ is significantly higher than that of Cnidarian GSDM_in_ (*p*-value of 2.17e-10) (Figure 1D), suggesting stronger positive selection pressures on Mollusca GSDM_in_. Pair-wise genetic distance analysis showed that GSDM_in_ is relatively more closely related to vertebrate GSDME and GSDMF (Figure 1E).

### Fish GSDME diversified into three distinct lineages and formed the root of tetrapod GSDMA/B/C/D lineages

**FIGURE 2:**
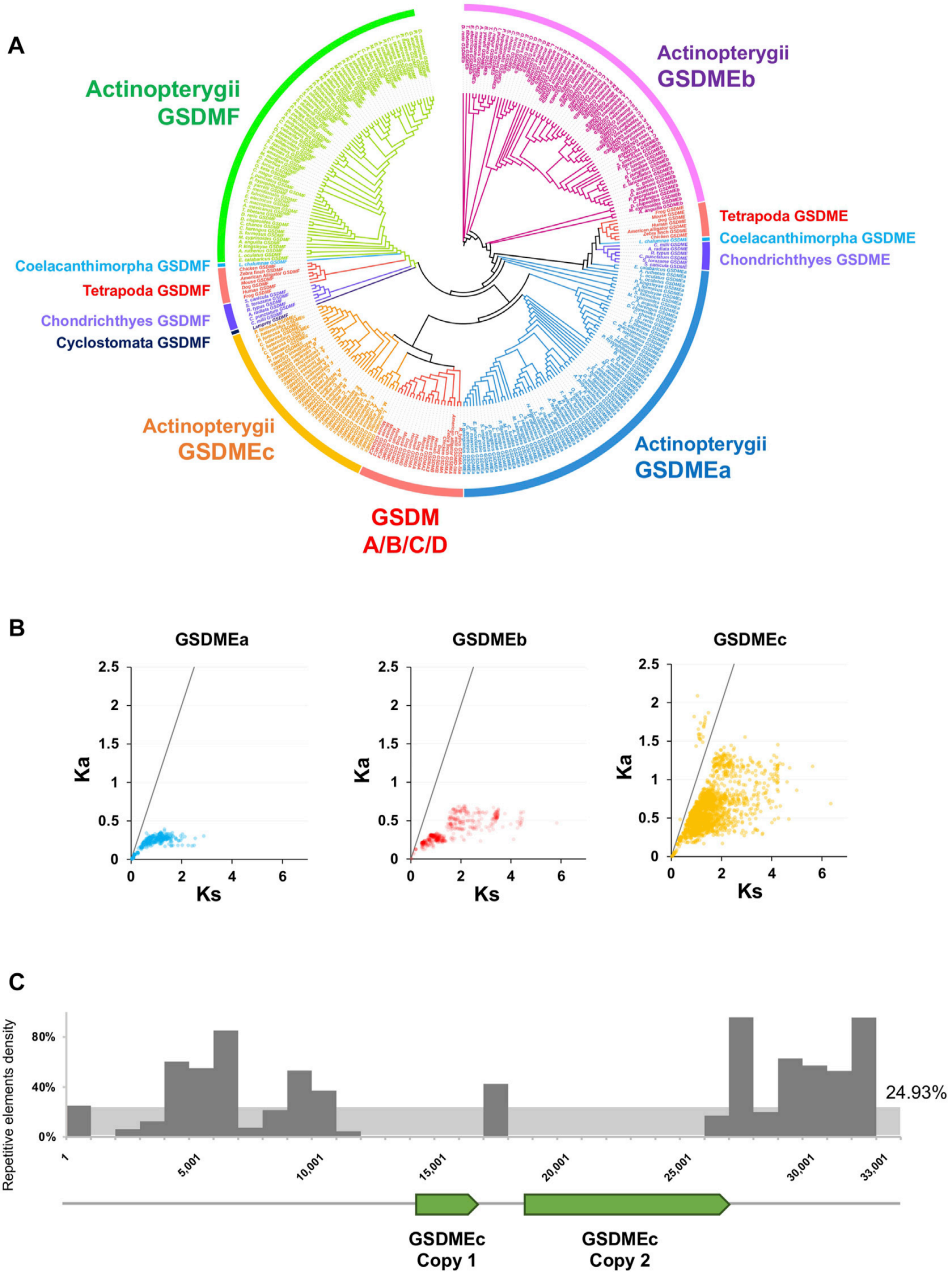
The phylogeny and evolutionary dynamics of GSDM in Actinopterygii. **(A)** The phylogenetic relationship between the GSDM of Actinopterygii and other vertebrate were conducted via JTT + F + R7 method with bootstrap of 1,000. Different clades are indicated by different colors. **(B)** The Ka/Ks ratios of GSDMEa, GSDMEb and GSDMEc in Actinopterygii. The black line represents Ka/Ks = 1. **(C)** The locations of the two GSDMEc copies and the density of the adjacent transposons in the *Poecilia formosa* reference genome scaffold 1,076 in a 1,000 bp bin. The *Y*-axis represents the percentage of repetitive elements.

We next investigated the evolution of GSDM in fish. Fish are representative organisms of lower vertebrate and comprise more than half of all living vertebrate species. Owing to their extensive genetic variations, fish are important models for studying gene evolution and function. Jawed fish, including Actinoperygii (accounting for 95% of the extant fish species), Sarcopterygii, and Chondrichthyes, contain the ancient GSDM family members, GSDME and GSDMF. However, in jawless fish, only GSDMF was found, which occurs in lamprey (Cyclostomata). GSDMF is highly conserved in fish and formed one phylogenetic clade with tetrapod GSDMF (Figure 2A, Supplementary Figure S14). By contrast, fish GSDME displays marked diversity in copy number and genetic variation. Both Sarcopterygii and Chondrichthyes have one GSDME ortholog, which are most closely related to tetrapod GSDME. The Sarcopterygii GSDME forms the phylogenetic basis for the tetrapod GSDME, and together they constitute a cluster that shares a common origin with the clade of Chondrichthyes GSDME. In contrast to Sarcopterygii and Chondrichthyes, Actinoperygii generally possess two GSDME orthologs, GSDMEa and GSDMEb, which have been observed previously in zebrafish (Broz et al., 2020). GSDMEa is highly diversified in Actinoperygii and has a close relationship with the GSDME of Chondrichthyes, Sarcopterygii, and Tetrapoda, while GSDMEb forms a clade of its own, which is constituted exclusively by teleost. In addition to GSDMEa and GSDMEb, we unexpectedly found a novel type of GSDM in Actinoperygii, mainly from the freshwater living perch-like species (Perciformes) and some primitive ray-finned fish, the latter including European eel *Anguilla* and reedfish *Erpetoichthys calabaricus.* This newly identified GSDM differs markedly from GSDMEa/b in sequence (average 25.4% identity) and was designated GSDMEc. GSDMEc forms a unique lineage distinctly separated from that of GSDMEb and GSDMF, but is clustered together with higher vertebrate GSDMA/B/C/D into one clade, which parallels the clade of some Actinoperygii GSDMEa (Figure 2A, Supplementary Figure S15). GSDMEc has a much higher Ka/Ks than GSDMEa and GSDMEb, suggesting rapid evolution of GSDMEc (Figure 2B). In *P. formosa*, two tandem copies of GSDMEc were found on Scaffold1076, which are flanked by repetitive elements including long interspersed nuclear element (LINE) sequences. These repetitive elements occur in much higher densities (over 80% in hot spots) than the genome background density (∼25%) (Figure 2C, Supplementary Table S2), suggesting a role of transposable elements in GSDMEc duplication.

### Actinoperygii GSDMEc and tetrapod GSDMA exhibit strong genomic collinearity

**FIGURE 3:**
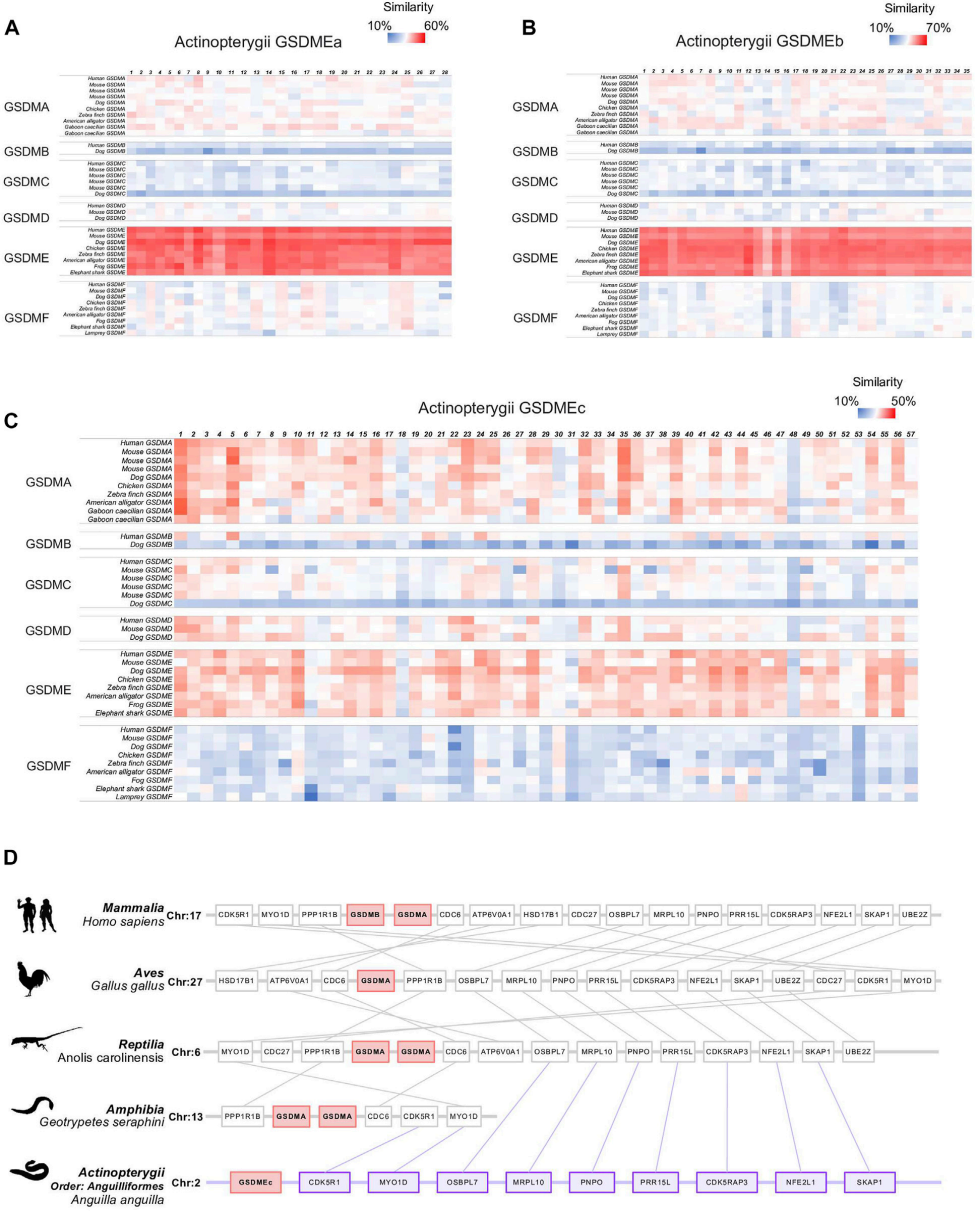
Sequence and synteny analysis of Actinopterygii GSDME in comparison with other vertebrate GSDM. **(A–C)**. Heatmap showing the sequence similarity between Actinopterygii GSDMEa **(A)**, GSDMEb **(B)**, and GSDMEc **(C)** and the GSDM from other vertebrates. **(D)**. Schematic diagrams showing the conserved neighbor genes of a representative Actinopterygii (*Anguilla*) GSDMEc in comparison with the conserved neighbor genes of GSDMA in Mammalia (*Homo sapiens*), Aves (*Gallus*), Reptilia (*Anolis carolinensis*), and Amphibia (*Geotrypetes seraphini*).

Different from GSDMEa and GSDMEb, which exist as a single copy in most Actinoperygii, GSDMEc exists generally in several duplicated copies that locate tandemly on the same chromosome. It is interesting that tandem duplications were also observed with GSDMA in tetrapod including reptile, birds, and mammals. It is believed that GSDMA has diverged into the mammalian GSDMB/C/D through gene duplication. These observations led us to examine the evolutionary relationship between GSDMEc and GSDMA. A large-scale pairwise sequence alignment of GSDM sequences from 228 species revealed that GSDMEc was closely and almost equally related to tetrapod GSDMA (average similarity 33.5%) and GSDME (average similarity 35.2%) (Figures 3A–C). In contrast, GSDMEa and GSDMEb were much more closely related to GSDME than to GSDMA. To date, the earliest emergence of GSDMA was documented in Reptilia, and no GSDMA was identified in Amphibia. In the present study, we detected no GSDMA in *Xenopus tropicalis*. However, we identified GSDMA in the Caecilian amphibians *Geotrypetes seraphini* (Family: Dermophiidae), *Microcaecilia unicolor* (Family: Siphonopidae) and *Rhinatrema bivittatum* (Family: Rhinatrematidae). Hence, GSDMA is present in all four tetrapod classes, implying the emergence of GSDMA in the common ancestor of tetrapod. With these findings, we then performed synteny analysis to further examine the evolutionary relationship between Actinopterygii GSDMEc and representative GSDMA from all tetrapod classes. We found markedly conserved genomic synteny proximal to GSDMEc and tetrapod GSDMA. For example, MYO1D and CDK5R1 was highly conserved around GSDMEc and the GSDMA of amphibia *G. seraphini*, reptile *Anolis carolinensis*, bird *Gallus*, and mammal *Homo sapiens* (Figure 3D). Collectively, these results, combined with that of phylogenetic analysis, suggest that Actinopterygii GSDMEc is the linkage root of higher vertebrate GSDMA/B/C/D.

### Functionally essential molecular patterns of GSDME are conserved across vertebrate and yet distinct in teleost

**FIGURE 4:**
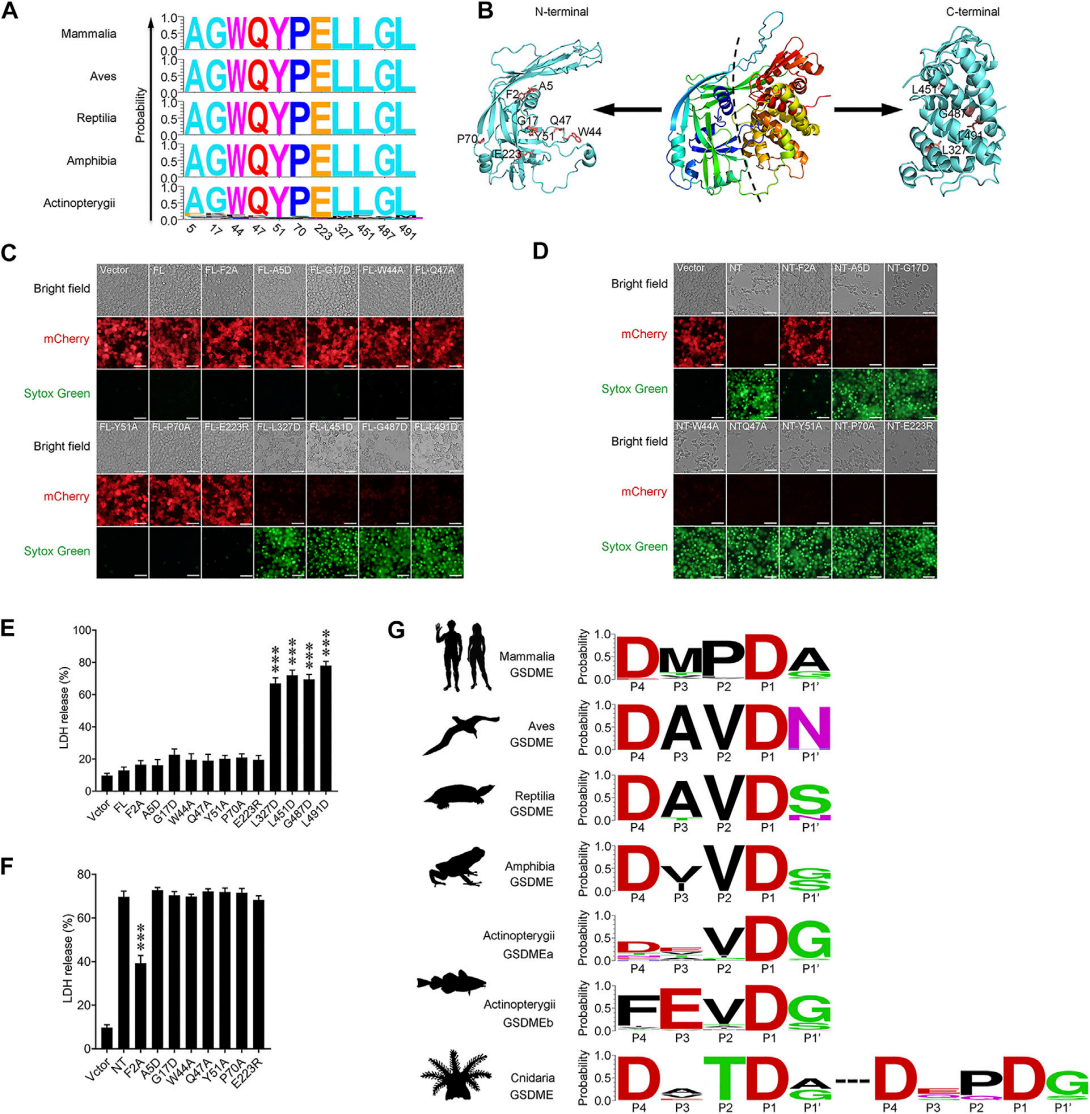
The identification and analysis of conserved GSDME motifs in vertebrate. **(A)** The 11 most conserved amino acid residues in Mammalia, Aves, Reptilia, Amphibia and Actinopterygii. The *X*-axis represents the position of the residue corresponding to that in human GSDME. The *Y*-axis represents probability. **(B)** The three dimensional structural model of human GSDME predicted by AlphaFold2. The 11 conserved residues, as well as Phe2, in the N and C terminal domains are labeled. **(C)** Pyroptosis analysis of full length GSDME (FL) with/without mutation at the 11 conserved residues. Images were captured with a confocal microscope under red (mCherry), green (Sytox Green), and bright field. **(D)** The analysis of the pyroptosis-inducing ability of GSDME N-terminal (NT) with/without mutation at seven conserved residues. Images were captured as above. **(E,F)** LDH release from the cells of **(C)** and **(D)** was measured and shown in **(E)** and **(F)**, respectively. Data are the means ± standard deviation of triplicate experiments. ***, *p* < 0.001. **(G)** The caspase consensus tetrapeptide motifs in the linker region of vertebrate GSDME. The motifs are represented by the amino acids in the P4–P1 and P1′ positions, with Asp set as P1.

GSDME is a conserved pore-forming member of the GSDM family existing across the vertebrate phyla. By comparing the GSDME sequences from Mammalia, Aves, Reptilia, Amphibia, and Actinopterygii, 11 residues that are top-ranked in conservedness were identified, and their functional importance in the pyroptotic activity of GSDME was examined (Figure 4A). Of these residues, A5, G17, W44, Q47, Y51, P70 and E223 are located in the NT domain, while L327, L451, G487 and L491 are located in the CT domain (Figure 4B). Unlike the wild type GSDME, which did not induce pyroptosis when expressed in HEK293T cells, GSDME mutants bearing L327D, L451D, G487D, and L491D substitutions exerted rigorous pyroptosis-inducing activity, despite their extremely low levels of expression (Figure 4C). Consistently, these cells released significant amounts of lactate dehydrogenase (LDH) and were stained positive by Sytox Green (Figures 4C,E). In contrast, GSDME mutants bearing A5D, G17D, W44A, Q47A, Y51A, P70A, and E223R substitutions failed to induce pyroptosis, despite their high levels of expression. Furthermore, introducing each of these mutations into GSDME-NT, the pyroptosis-inducing domain of GSDME, had no effect on the pyroptotic activity of GSDME-NT (Figures 4D,F). It is known that cleavage at the inter-domain linker region to release the NT fragment is essential for GSDM to execute pyroptosis. Analysis of the GSDME sequences from different vertebrate animals revealed a conserved tetrapeptide motif _P4_DxxD_P1_ (x represents any amino acid), which is well fitted to the consensus recognition site of the caspase-3/7, in Mammalia, Aves, Reptilia and Amphibia (Figure 4G). In Actinopterygii, this caspase-3/7 recognition motif is conserved in GSDMEa; however, a caspase-1 recognition consensus motif, _P4_FxxD_P1_, is prevalent in GSDMEb (Figure 4G), whereas no apparent caspase recognition motif was identified in the linker region of GSDMEc. In invertebrate, as shown in Figure 1, Cnidaria and Mollusca are the two phyla from which most of the GSDM_in_ were identified. Cnidaria GSDM_in_ display the caspase-3/7 recognition tetrapeptide, _P4_DxxD_P1,_ as two adjacent motifs in the linker region (Figure 4G). In contrary to Cnidaria, Mollusca GSDM_in_ exhibit no evident caspase recognition site in the linker region.

## Discussion

Study of GSDM in keystone species is vital to the understanding of the evolution of pyroptosis (De Schutter et al., 2021). In this work, we conducted a systematic analysis of GSDM across over 30 major metazoan phyla. We found that GSDM_in_ occurs in 12 phyla, and the earliest form of GSDM_in_ was detected in Placozoa *T. adhaerens*, indicating an ancient nature of GSDM in metazoan. Absences of GSDM_in_ were observed across the invertebrate phyla, especially in the Ecdysozoa group, which includes some largest animal phyla such as Arthropoda and Nematoda. It is possible that in these species, GSDM-mediated function may have lost during evolution or been effected through alternative mechanisms involving no GSDM. Of the identified GSDM_in_, most were derived from Cnidaria and Mollusca, the latter exhibiting much higher Ka/Ks ratios, which could be the result of stronger positive selections. Since Mollusca is the second largest animal phylum, its high species diversity may also have a contribution to the high genetic variation of GSDME_in_ (Liu et al., 2021b). Apart from Metazoa, GSDM-like proteins were recently reported to exist in fungi and bacteria (Daskalov et al., 2020; Johnson et al., 2022). However, these proteins share extraordinarily low sequence identities with metazoan GSDM and are completely separated from metazoan GSDM in phylogeny, supporting the notion that GSDM and GSDM-like pore-forming proteins have diverse evolutionary origins (Dal Peraro and Van Der Goot, 2016).

Fish of various groups account for more than half of all vertebrate species and have been used widely as important models to study evolutionary transitions from invertebrate to vertebrate (Bi et al., 2021; Vernaz et al., 2021). In our study, only the most primitive forms of GSDM, GSDME and GSDMF, were found in fish. GSDMF is remarkably conserved in fish and most closely related to tetrapod GSDMF, implying highly conserved evolution of GSDMF in vertebrate. Actinopterygii, the largest clade of fish, exhibit dynamic evolution of GSDME. Previous reports showed that zebrafish has two forms of GSDME named GSDMEa and GSDMEb (Wang et al., 2017), probably due to the fish-specific whole genome duplication (Meyer and Van de Peer, 2005; Kasahara et al., 2007). In this study, we found that the occurrence of GSDMEa and GSDMEb is a general genetic feature of Actinopterygii. These two orthologs were classified into two widely separated phylogenic groups, suggesting an early divergence of GSDME in fish. Unexpectedly, in addition to GSDMEa and GSDMEb, we also identified a third type of GSDME, named GSDMEc, mainly in freshwater perch-like species and some primitive ray-finned fishes. Phylogenetically, GSDMEc is distinctly separated from GSDMEa/b, and closely related to higher vertebrate GSDMA/B/C/D. This discovery suggests that GSDMEc and the advanced forms of tetrapod GSDM may have descended from a common ancestor of Euteleostomi and evolved independently over time. Compared to GSDMEa and GSDMEb, GSDMEc exhibits much higher Ka/Ks ratios and, in some species of Poeciliini, exists in tandem duplication on the same chromosome. A similar pattern of gene duplication was also observed with the GSDMA in mammals (Tamura et al., 2007). As the ancestor type of GSDMB/C/D, GSDMA was previously reported to be formed after the divergence of Lissamphibians based on the evolutionary analysis using the Anura species *Xenopus laevis* as the model organism (De Schutter et al., 2021; Angosto-Bazarra et al., 2022). In our study, we traced the earlier form of GSDMA in some Gymnophiona species including *M. unicolor, R. bivittatum* and *G. seraphini*, thus indicating the ubiquitous presence of GSDMA in all tetrapod classes. This finding fills the gap of GSDMA evolution in tetrapod and suggests the occurrence of GSDMA in the common ancestor of tetrapods. In line with these results, we found marked syntenic conservation between Actinopterygii GSDMEc and tetrapod GSDMA. Together, our results suggest that GSDMEc is probably the prototype of GSDMA, and also lend support to the current belief that GSDMA has diverged into GSDMB/C/D.

Transposable elements are believed to be the driving force of genetic diversification and generation of new genes (Cerbin and Jiang, 2018; Yuan et al., 2018; Shao et al., 2019; Cosby et al., 2021). In the reference genome of the well-studied model organism *P. formosa* (Lampert and Schartl, 2008), we found high densities of transposable elements around the two adjacent GSDMEc on scaffold 1,076, suggesting that transposable elements may be one of the evolutionary propelling forces that have driven the duplication of GSDMEc in *P. formosa*. Previous studies indicated that in fish, the proportion of repetitive elements is general positively correlates with genome size (Shao et al., 2019). Consistently, in the present study, we found that GSDMEc-harboring species are predominately fresh-water species with relatively complex genomes and large genome sizes (Average 946.5 MB). The fresh-water species are known to be rich in repetitive elements in the genomes, which lead to fast evolution to adapt to the drastic changing environments (Streelman et al., 1998; Wei et al., 2014). In contrast, Actinopterygii with small genomes, such as the Tetraodontiformes species, which have genome sizes around 400 MB (Neafsey and Palumbi, 2003), lack GSDMEc.

As an ancient member of GSDM, GSDME possesses genetic sites highly conserved in vertebrate (Xia et al., 2019). In this study, we identified 11 top-ranked conserved amino acid residues in vertebrates. Although the structure of GSDME remains elusive, the structures of other GSDM members reveal that the auto-inhibition of GSDM is mainly achieved by the CT domain (Ding et al., 2016). In agreement, our results showed that the CT domain was essential to auto-inhibition. Based on the structural models of GSDMA3 and GSDMD (Ruan et al., 2018; Xia et al., 2021), the main function of the GSDM-NT domain is pore forming by adopting transmembrane *β*-strands. We found that the highly conserved residues in the GSDME NT domain (A5, G17, W44, Q47, Y51, P70 and E223) are probably not located within the transmembrane *β*-strands, and therefore may be not involved in the pore-forming activity. These results are useful for future studies on the structure and function of GSDME, especially concerning the CT region. In addition, we also observed conserved caspase recognition patterns in the linker region of GSDME in higher and lower vertebrates. Caspase-3 is known to cleave after the tetrapeptide motifs DMPD and DMLD in human and mouse GSDME, respectively (Rogers et al., 2017; Wang et al., 2017). Accordingly, we found that there exists a highly conserved caspase-3 recognition motif, DxxD, in the linker region of mammalian GSDME, indicating that caspase-3 activated GSDME-mediated pyroptosis is likely conserved in mammal. Although the function of GSDME in bird, reptile and amphibian is unknown, we found a conserved DxxD motif in the linker region of GSDME in these animals, suggesting the existence of a caspase-3-mediated GSDME cleavage mode of pyroptosis activation in tetrapod. In fish, the DxxD motif is also conserved, but only in GSDMEa. Different from all other GSDME, GSDMEb exhibits a unique conserved FxxD motif in the linker region, which is similar to the caspase-1 consensus recognition motif (FLTD) in human GSMD (Shi et al., 2015). This observation indicates that caspase-1 may play an important role in fish GSDMEb activation. It has been suggested that zebrafish GSDMEb might act as a functional homologue of mammalian GSDMD if it could be cleaved by caspase-1 homologue (Broz et al., 2020). In zebrafish, IL-1β secretion was reported to be associated with GSDMEb-mediated pyroptosis, suggesting that GSDMEb-formed transmembrane channels may serve as the secretion path of intracellular cytokines (Li et al., 2020). Our results indicated that indeed, GSDMEb might function as a GSDMD homologue, however, since GSDMEb is far separated from GSDMD in phylogeny, the evolutionary paths that led to the caspase-1 recognition feature in GSDMEb and GSDMD may be different. In contrast to GSDMEa and GSDMEb, GSDMEc exhibits no obvious caspase recognition site in the linker region, implying that there may exist an unknown cleavage mechanism of GSDMEc activation. Based on these findings, as well as the previous reports that GSDMEa was cleaved by caspase-3 and GSDMEb (or its equivalent) was cleaved by caspase-1 (or its functional analog) in zebrafish and tongue sole (Wang et al., 2017; Jiang et al., 2019; Li et al., 2020; Wang et al., 2020), it is conceivable that complicated scenarios of GSDME cleavage and pyroptosis activation exist in fish. In invertebrate, we found that coral possess two tandem DxxD motifs in the linker region of GSDM, which have been shown to be specifically cleaved by caspase-3 (Jiang et al., 2020). Collectively, our observations in invertebrate and vertebrate indicate that caspase-3-mediated GSDME activation is probably an evolutionarily conserved event in the pathway of pyroptosis.

In conclusion, in this study we demonstrated that GSDM emerged early in invertebrate Metazoan and has undergone dynamic evolution. Teleost GSDME duplicated and diversified into three orthologs that have evolved separately into distinct lineages. A previously unidentified fish GSDME, GSDMEc, forms the lineage root of higher vertebrate GSDMA/B/C/D and is probably the prototype of tetrapod GSDMA. We also provided evidences that GSDMA had emerged in the common ancestor of all four, rather than three, tetrapod classes. In addition, our study identified both conserved and unique function-essential genetic patterns of GSDME in different vertebrate clades. Together, these results shed new light on the origin, evolution and function of GSDM.

## Data Availability

The datasets presented in this study can be found in online repositories. The names of the repository/repositories and accession number(s) can be found in the article/Supplementary Material.
