# Supplementary material for: New insights into the evolutionary dynamic and lineage divergence of gasdermin E in metazoa
Source: Front Cell Dev Biol. 2022 Jul 22;10:952015. doi: 10.3389/fcell.2022.952015 (PMC9355259; doi:10.3389/fcell.2022.952015)
Supplement: Supplementary file 1 [file DataSheet1.PDF]

Supplementary Materials for

New insights into the evolutionary dynamic and lineage divergence of gasdermin E in Metazoa

Zihao Yuan[a,b,1], Shuai Jiang[a,b,c,1,*], Kunpeng Qin[a,b,c], Li Sun[a,b,c,*]

a. CAS and Shandong Province Key Laboratory of Experimental Marine Biology, Institute of Oceanology; CAS Center for Ocean Mega-Science, Chinese Academy of Sciences, Qingdao, China.
b. Laboratory for Marine Biology and Biotechnology, Pilot National Laboratory for Marine Science and Technology, Qingdao, China.
c. College of Earth and Planetary Sciences, University of Chinese Academy of Sciences, Beijing, China

Z.Y[1] and S.J[1] contributed equally to this work.
*Correspondence:
Prof. Li Sun
Institute of Oceanology, Chinese Academy of Sciences;
7 Nanhai Road, Qingdao 266071, China;
Email: lsun@qdio.ac.cn
Prof. Shuai Jiang
Institute of Oceanology, Chinese Academy of Sciences;
7 Nanhai Road, Qingdao 266071, China;
Email: sjiang@qdio.ac.cn

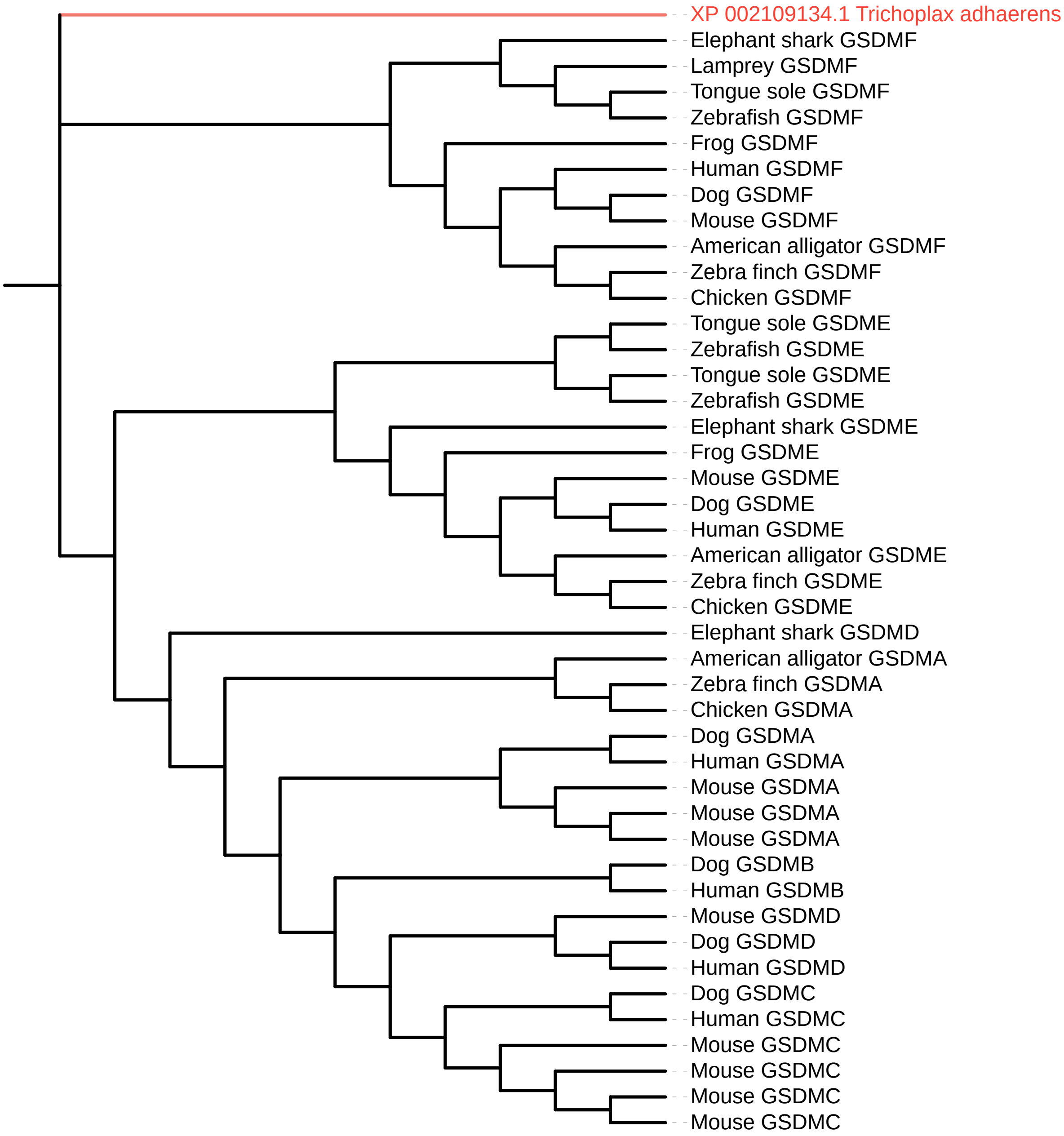

Fig. S1. The phylogenetic tree based on Placozoa and vertebrate gasdermins. The Placozoa gasdermin is marked in red, and the representative vertebrate gasdermins are marked in black. The bootstrap of 1000 and the method of JTT+F+R4 were applied.

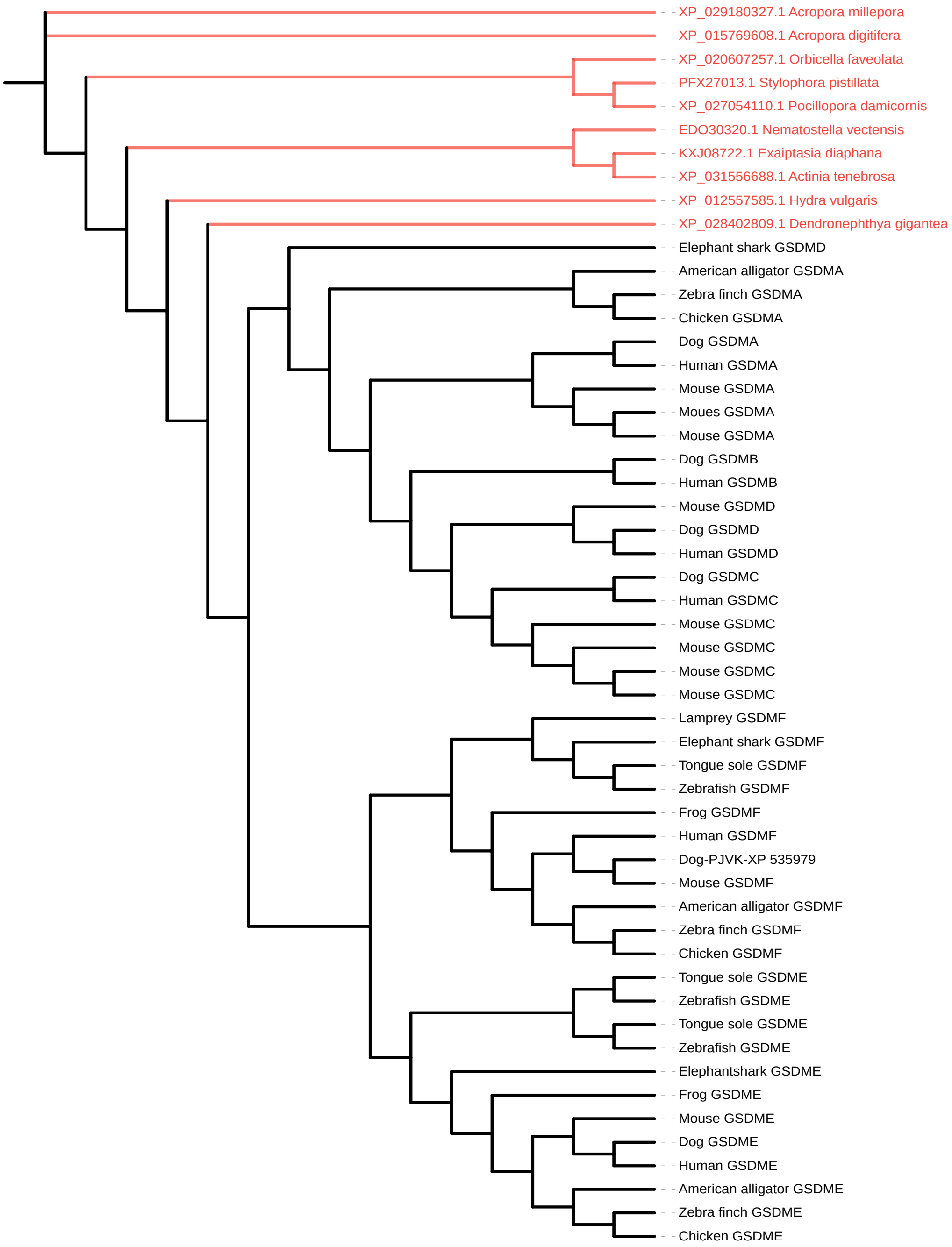

Fig. S2. The phylogenetic tree based on Cnidaria and vertebrate gasdermins. The Cnidaria gasdermins are marked in red, and the representative vertebrate gasdermins are marked in black. The bootstrap of 1000 and the method of JTT+F+G4 were applied.

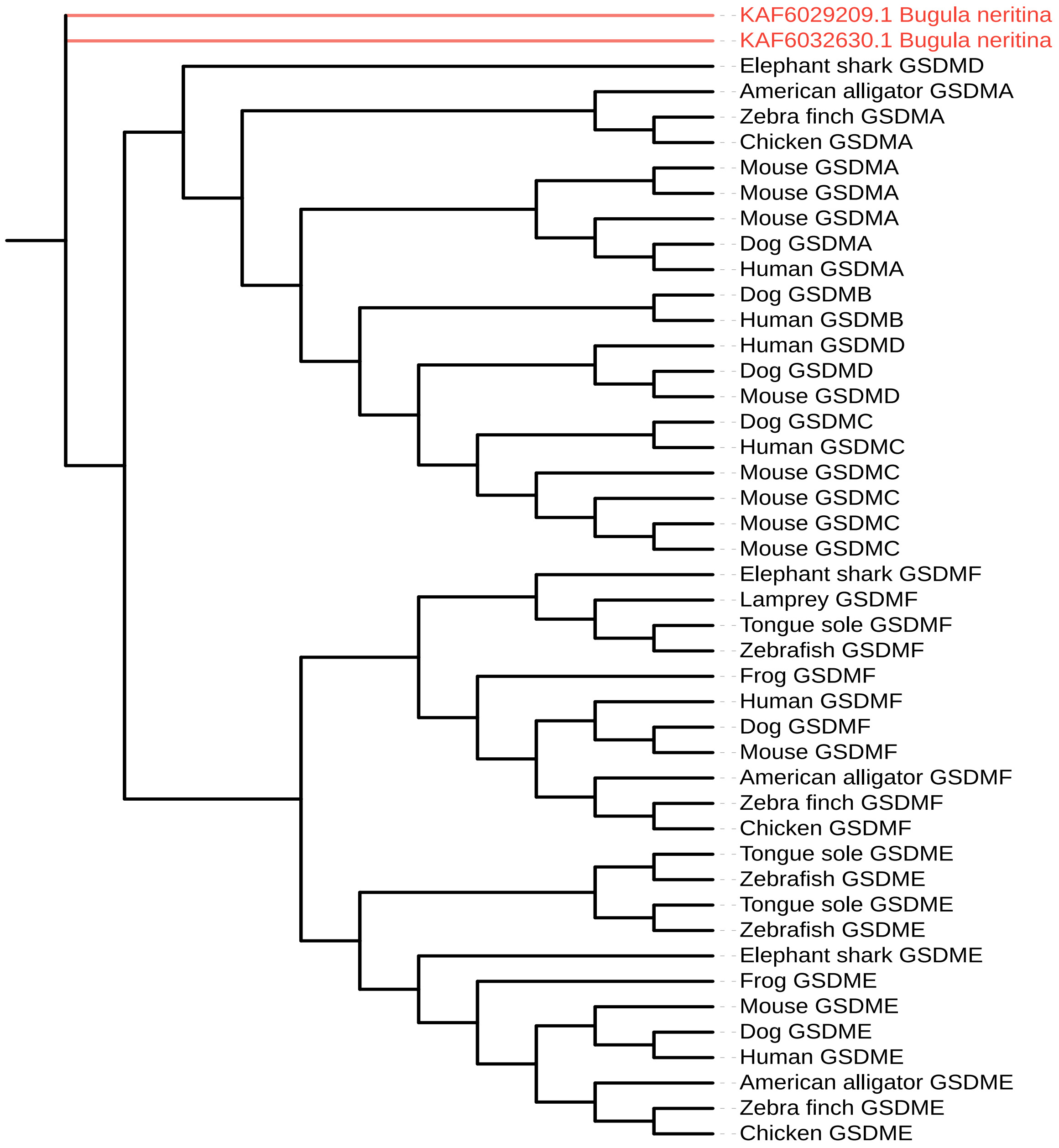

Fig. S3. The phylogenetic tree based on Bryozoa and vertebrate gasdermins. The Bryozoa gasdermins are marked in red, and the representative vertebrate gasdermins are marked in black. The bootstrap of 1000 and the method of JTT+F+R4 were applied.

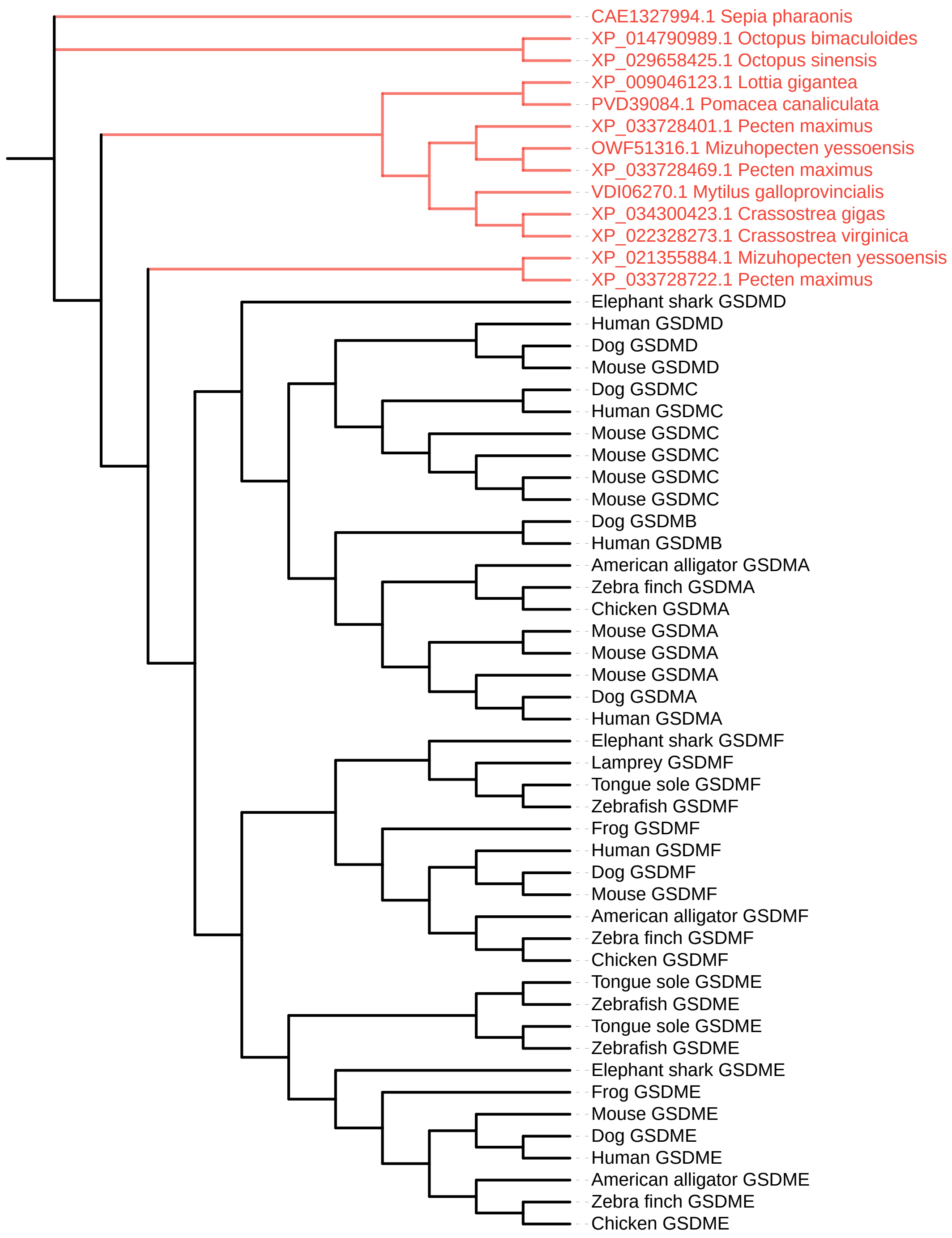

Fig. S4. The phylogenetic tree based on Mollusca and vertebrate gasdermins. The Mollusca gasdermins are marked in red, and the representative vertebrate gasdermins are marked in black. The bootstrap of 1000 and the method of JTT+F+R4 were applied.

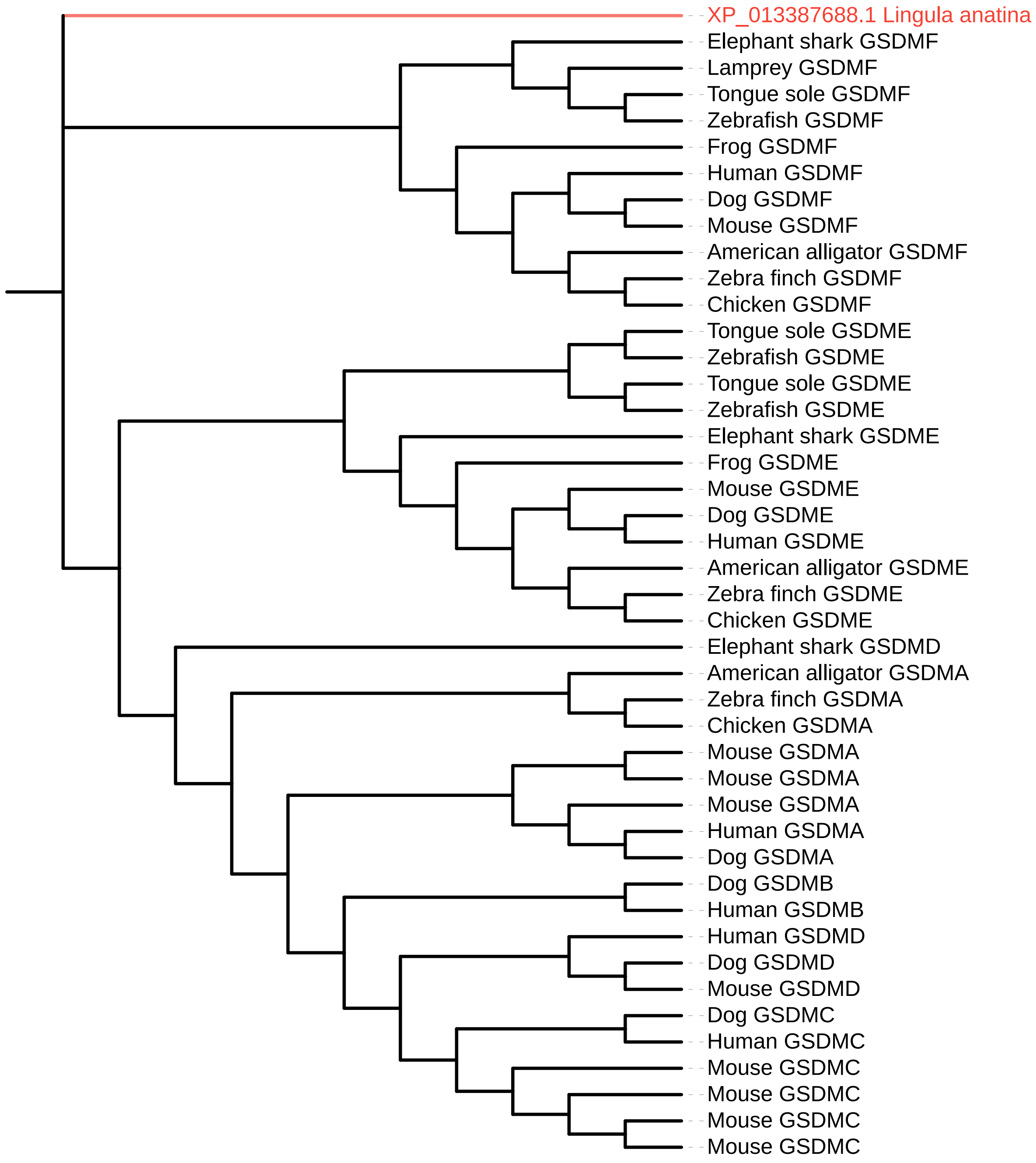

Fig. S5. The phylogenetic tree based on Brachiopoda and vertebrate gasdermins. The Brachiopoda gasdermin is marked in red, and the representative vertebrate gasdermins are marked in black. The bootstrap of 1000 and the method of JTT+F+R4 were applied.

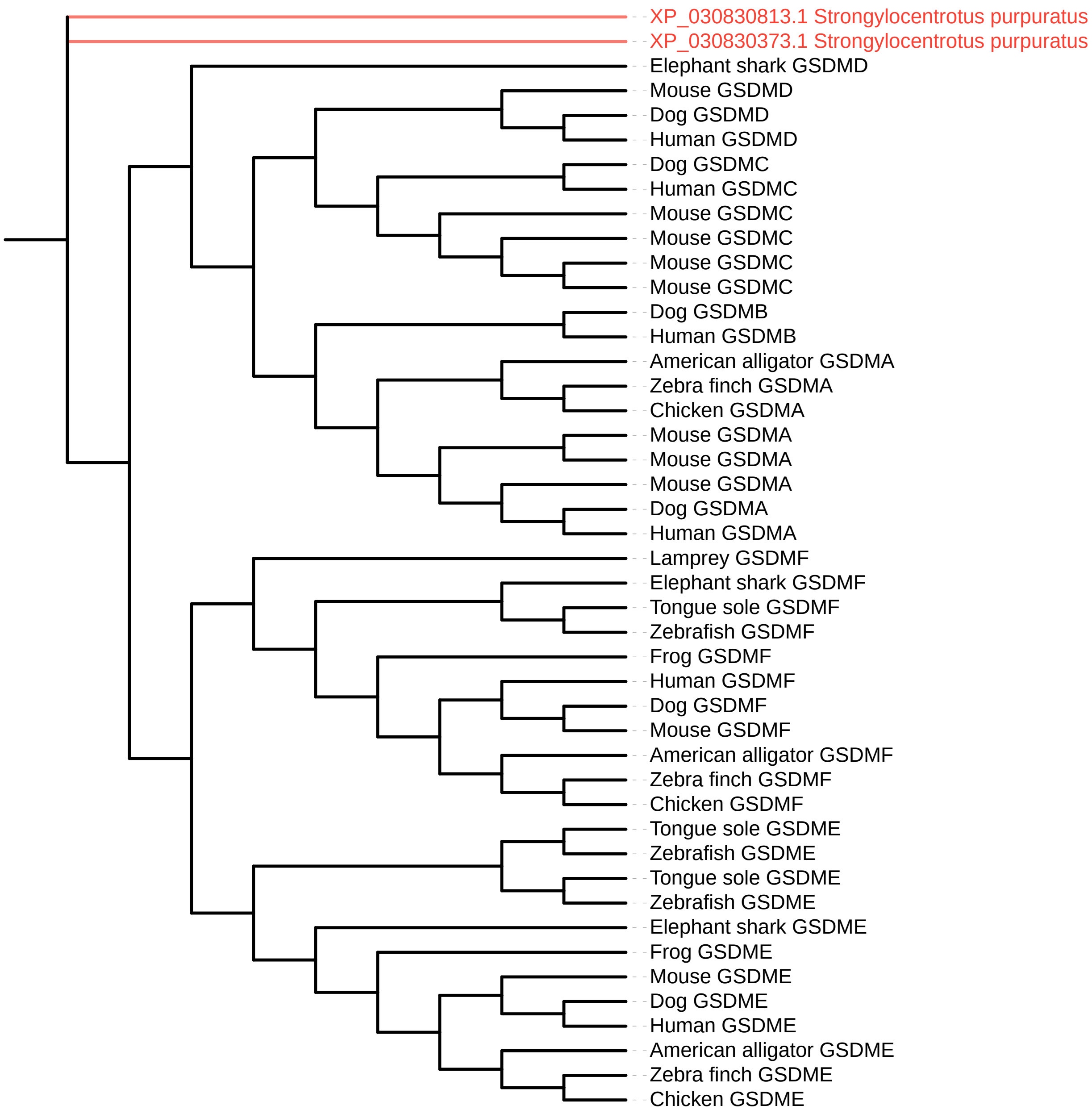

Fig. S6. The phylogenetic tree based on Echinodermata and vertebrate gasdermins. The Echinodermata gasdermins are marked in red, and the representative vertebrate gasdermins are marked in black. The bootstrap of 1000 and the method of JTT+F+G4 were applied.

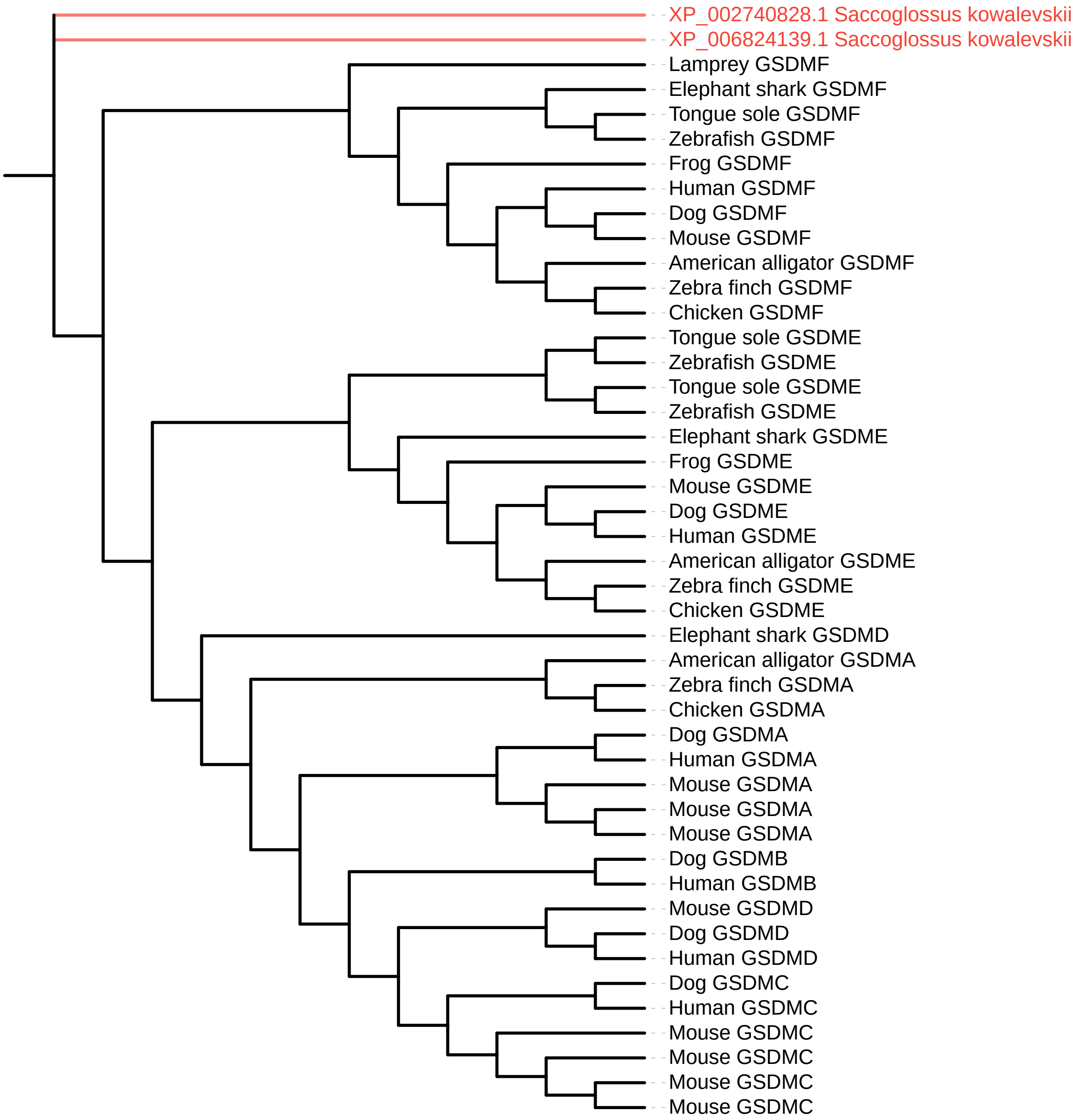

Fig. S7. The phylogenetic tree based on Hemichordata and vertebrate gasdermins. The Hemichordata gasdermins are marked in red, and the representative vertebrate gasdermins are marked in black. The bootstrap of 1000 and the method of JTT+F+R4 were applied.

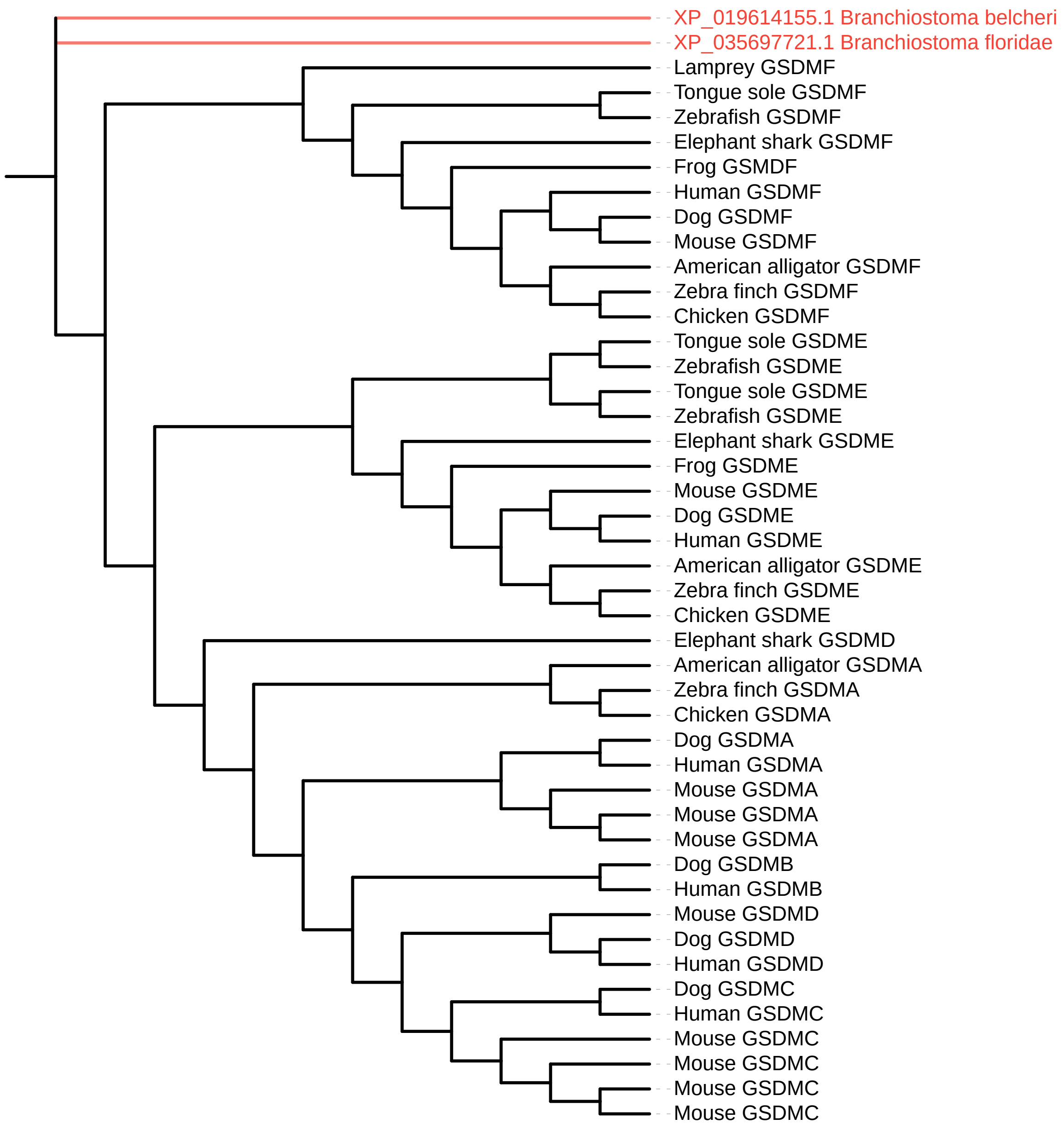

Fig. S8. The phylogenetic tree based on Cephalochordate and vertebrate gasdermins. The Cephalochordate gasdermins are marked in red, and the representative vertebrate gasdermins are marked in black. The bootstrap of 1000 and the method of JTT+F+R4 were applied.

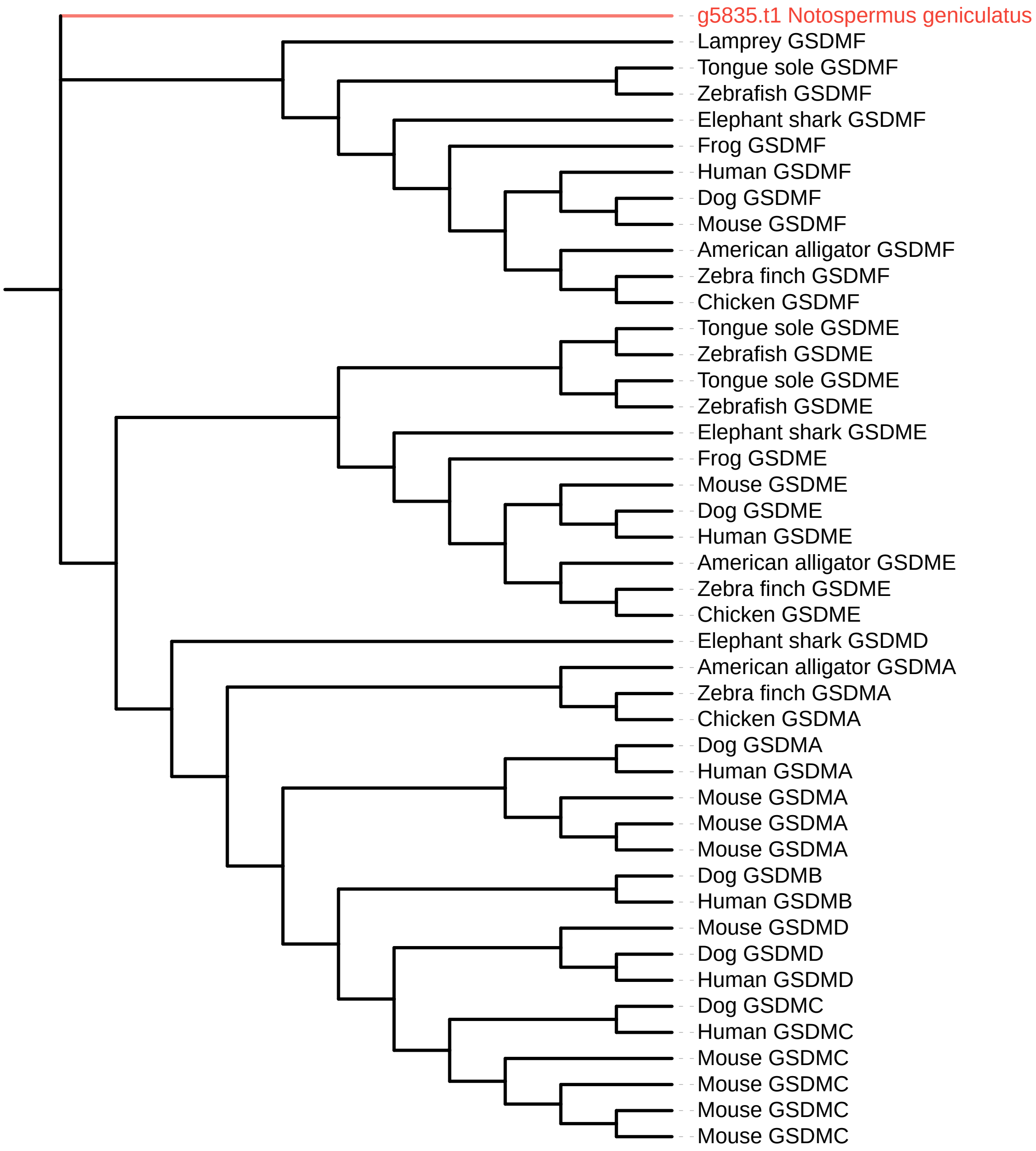

Fig. S9. The phylogenetic tree based on Nemertea and vertebrate gasdermins. The Nemertea gasdermin is marked in red, and the representative vertebrate gasdermins are marked in black. The bootstrap of 1000 and the method of JTT+F+R4 were applied.

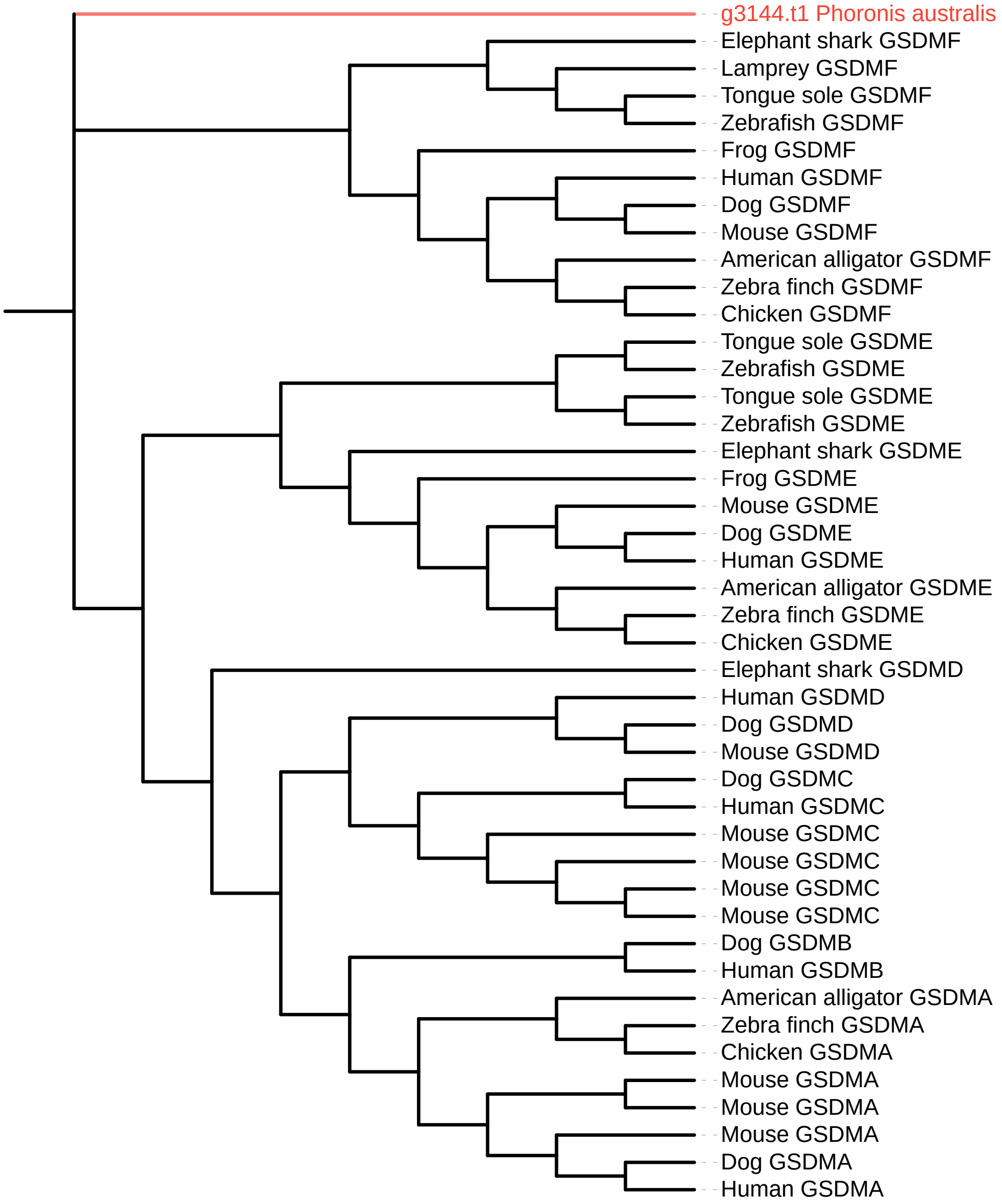

Fig. S10. The phylogenetic tree based on Phoronida and vertebrate gasdermins. The Phoronida gasdermin is marked in red, and the representative vertebrate gasdermins are marked in black. The bootstrap of 1000 and the method of JTT+F+R4 were applied.

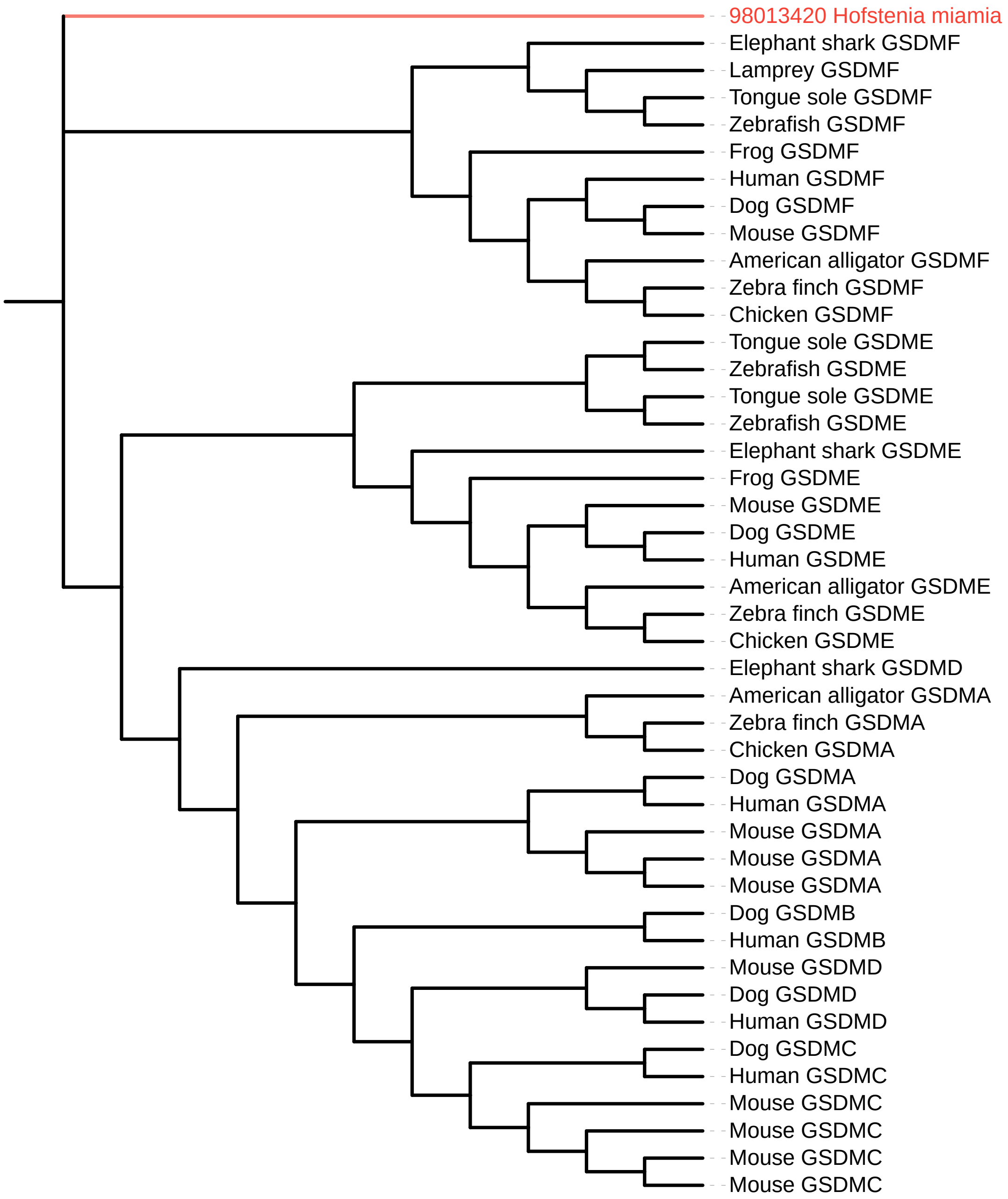

Fig. S11. The phylogenetic tree based on Xenacoelomorpha and vertebrate gasdermins. The Xenacoelomorpha gasdermin is marked in red, and the representative vertebrate gasdermins are marked in black. The bootstrap of 1000 and the method of JTT+F+R4 were applied.

```
XP_015769608.1     1 ---MAVFECCAKQFVKDTGRLTLRPVVDLNSAIRFDILCVVIKKRSRWIWKSV---KYEA
XP_029180327.1     1 ---MAVFECCTKQFVKDTGRFTLRPVVDLNSAIRFNILCVVIKKRSRWIWKSV---KYEA
XP_027054110.1     1 MVNMALFEACSKQFIKDTGRSTLRPILDLNTSTRCSILCVVTRKRSRWFWKAD---TYKS
PFX27013.1         1 ---MALFEACSKQFVKDTGRSTLRPILDLNTSTRSSILCVVTKKRSRWFWKTD---TYKS
XP_020607257.1     1 ---MALFEACAKQFVKDTGRSTLFPVLDLNTSARCDILCVVTKKRSRWLWKSD---KYKT
XP_031556688.1     1 ---MALFESASKQLVKDTGRSSLHPVLDLNSSSKYGIMCVVEKKKSRWFWRKT---KYLP
KXJ08722.1         1 ---MALFESTSKQLVKDTGRSSLHPIVDLNSASQCKIMCIIEKKKSRWFWRPT---KYLP
EDO30320.1         1 ---MALFESASKQFVKDTGRRSLHAVPDLNSSECCRILCVIERKKSRWFWRST---KYLT
XP_012557585.1     1 ---MAMFEAATKCIAKSIGSKTLHHVSDLNSSERFKLLCVVCQKKSFWPWKKT---QIFP
XP_028402809.1     1 ---MSLFDATVSQVVKDVSHDTLVPVRNRCAADYCKLMSLVERKTRCWHLPWKSKYKYIP

XP_015769608.1    55 TPFTLNEILTKPVDCVSKSEKSVFIRNYKNEPEFNVKGKLGGKLAQYFGIDASCIDSFTV
XP_029180327.1    55 TPFTLNEILTKPVDCVSKFEKSVFIRNYRNEPKFDVKGKLGGKLAQYFGIDASCIDSFTV
XP_027054110.1    58 TSFTLNELLTKPVLIKGHIEKSTFISDYKNEPKFHVSGKLGGKIASEFGIDASAIDSFTI
PFX27013.1        55 TPFTLNELLTKPVHIKGHIEKSTFISNYKNEPKFHISGKLGGKIASEFGIDASAVDSFTI
XP_020607257.1    55 TPFSLNELLTKPVDISGQIKKSVFIANYKNEPKFHVSGKLGAKIATEFGIDVSAIDSFTI
XP_031556688.1    55 TPFNLNEILLQPIDYSSTVKTTEFVANYENDPSLHVSGKLGAKLAGELGIDVSTTDSFTV
KXJ08722.1        55 TPFTLNELMTKPIDVSKKIKTSEFVSNYSNQPTLHVSGKLGGKIAKELGIDVSTADSFVV
EDO30320.1        55 TPFVLNELLTEPVDLKEKQKEEVFITDYQNNPQFHVSGKLGAKIAKDLGIDVSTTDSFVV
XP_012557585.1    55 TQIVLQDILLTKLNIEQSDVNFTLVKDYIENPTLNIKGDVGAKIASELQLEFSASDSVSL
XP_028402809.1    58 AETTLNDILTKEVDVKSHFCTSVLFKDYASTPSFHKAGKIGGKIAEEFDVDVSINETMQV

XP_015769608.1   115 DMNVGTVTKREVKWDVLNQLLADNTLNVDHEFVQTILGKPRRSLCIVYETVSTGRDADVD
XP_029180327.1   115 DMNVGTVTKREVKWDVLNQLLADNTLNVDHEFVQTILGKPRQSLCIVYETVSTGRDADVD
XP_027054110.1   118 SMDVGTVLKREVTWDNLKKVLADTTLNLDHEYVQYILAKQRRSICVIYETVATKGDTDLD
PFX27013.1       115 SMDVGTVLKREVTWVNLKKALADTTLNLGHEYVQYICTKQRRSLCIIYETVATQGDTDLA
XP_020607257.1   115 SMDVGDVIKKQVHWGNLNDALADITLNLDHEYVQYILSKPRRSLCVIYETVATHGDSELD
XP_031556688.1   115 KINLGTINKSKIRWQELSNLMKDKKLNLEHEFVQAILKSKRRKLCIVNEVLKTTKETNIQ
KXJ08722.1       115 KMDLGSINKSEIRWQDLNDALKDQTLKLDHEFVQAILNCRRRSLCIVFETLNTTKDTNIQ
EDO30320.1       115 KMNIGAVNKTEIRWQDMFSALKGKRLNVDHEFVQAIIASKRRSLSVVCEMLATTGDTKME
XP_012557585.1   115 NINVGSLQKQEIKWQKLDNALQSSKVNTRHPLLEEIQFLKRTELAVVLESISTCADSTLS
XP_028402809.1   118 SLKLGDVIKREVVWQELYDCVNGKAAKGDHPLLKTIKSSTKKSLFLIGMVVTSGVITSLD

XP_015769608.1   175 --SDADQEGDANVTG-GKSEFSINLSGSVQAKHHRSFELPNHTILGFACYEITIDPD---
XP_029180327.1   175 --SDADQEGDANVTG-GKSKFSINLSGSVQAEHHRSFELPNHTILGFACYEMTIDAG---
XP_027054110.1   178 --SDSKEEGDASVSV-GKPKFFIKLTGSIELEHHRSYELPSNTILGYACYEVKFDPD---
PFX27013.1       175 --SDSREEGDASVST-GKPKFSISLSGSVELEHHRSYELPSNTILGYACYEAQFDLD---
XP_020607257.1   175 --SDSSGQGDASLNA-GKPTFSINLSGSVQVAHHRSFELPNNTILGYACNEITFDSA---
XP_031556688.1   175 --SVLIVEGDADAKASATTKAEVDVKGSVSDTHHHSFDIPKDTIXAYGCYEFDFDDG-IG
KXJ08722.1       175 --SDIQVEGDAVASTDIISKASVNISGSIKDTHHHSFDIPNGTVMAYGCYGLKLLEG-LG
EDO30320.1       175 --SELQVEGDADIETTGIPMASGKVEGSVKDSHQRSFIIPKGTVLGYGCYRLRIVDKDQG
XP_012557585.1   175 GSADLNVTTDDSVSNKSVVVVDVHTKDSIEKKTTHSYTLPSNTVLAFSCNTFSVTEYG--
XP_028402809.1   178 ----KETKFGGDLGGTPHVQLTLNSDGSSDAKQHRQYTIPPGTTLAYCCYKLKVGND---

XP_015769608.1   229 ----LGTFQLLLPDS---------------------------------------------
XP_029180327.1   229 ----LGTFQLVLPDSSSFTSDLSLSLLID---------KALAALFDDLFNSPYCSKIVQAF
XP_027054110.1   232 ----MGTFELVLPDETDAGGEIGK-KYLFDEPDGQKDKALSAVFDDLLKSPRLGKIISLY
PFX27013.1       229 ----TGTFELVLPDATDGGGEVGMNYCLFDEPDGQKDKALKAVFDDLLKSPLLGKIIGLY
XP_020607257.1   229 ----MGTFQLVLADATDGGGMSTRECCVFDEPDGQNDKALKAVFDDLLNSPLCSKIFDLY
XP_031556688.1   232 MIDLAIEKKLEDVT-----DAAPSDETTVDHPDGQ-DSALTSIFHGLLKSPKKDDVIKCF
KXJ08722.1       232 LIELDVDKKLKDVVT-DATDGKPPDAFSMDQPDGP-DTALTSAFNALMKSPHKGDIIKCF
EDO30320.1       233 SVEMDIDKELEDKDVTDAVDGAPTAAHPFDQPDGQNDSALKSEFKTLLESPSFGKIIECF
XP_012557585.1   233 ----------GIDFHAAPDEADFSTKTLLQKTKKDSFDFVKSMLFSFVKSSKFSSIQQFF
XP_028402809.1   231 ------------GVLNLQLPDDLTDAPLPAKEKIFEVESIQQALSGLLTNEANDDLIILL

XP_015769608.1   240 ----------------------IDGGDPQEFKKHCVFDEPDGQN--A-------------
XP_029180327.1   277 REILAYPP--AIPPLRDLLEQALSCCEEKNFKALALAKFKTGVG--AGYKSCEDLLTLLG
XP_027054110.1   287 KEILSYPE--AVAPLRDMLEKALSAVEKKSFKPLEMKEFQTKAG--PAFGSLKELLLLIG
PFX27013.1       285 KEILSYPQ--AVAPLRDMLEKALSAVEKKNFEPLEMKEFQTKAG--PGYGPLKDLLPLIG
XP_020607257.1   285 RKILSCPA--AVAPLRDLLEKGLSSAEKKNPKPFELKELKTKAG--AAYENCKELFLLLG
XP_031556688.1   286 KQILATPE--SIKPLDELLEKAVYSVEGEMVKPYTFQEFKSLVE--HCEG-WEKLLALLG
KXJ08722.1       290 REILATPE--YCHVIEQLLDNVCDYFDKEKVKSYSLDEVKSLFG--SCNS-WERLLTILG
EDO30320.1       293 RNILASPG--HIKPLRDLLEDSVLSLDGEKVNALKEDAFLDSMG--PCQG-WRELLDIIG
XP_012557585.1   283 KSIIGSVEQSSLEAIYLLLLAVEGSSENNFYLNSCHSALSKTIV--ENIGLWKPLLLSLN
XP_028402809.1   279 RKILNCRP-ACAFKLNIVVENAIDILQGESVLCTITIPHLKKIFTEDCFPDCFKFLEIAG
```

Fig. S12. Alignment of 10 Cnidaria GSDM sequences.

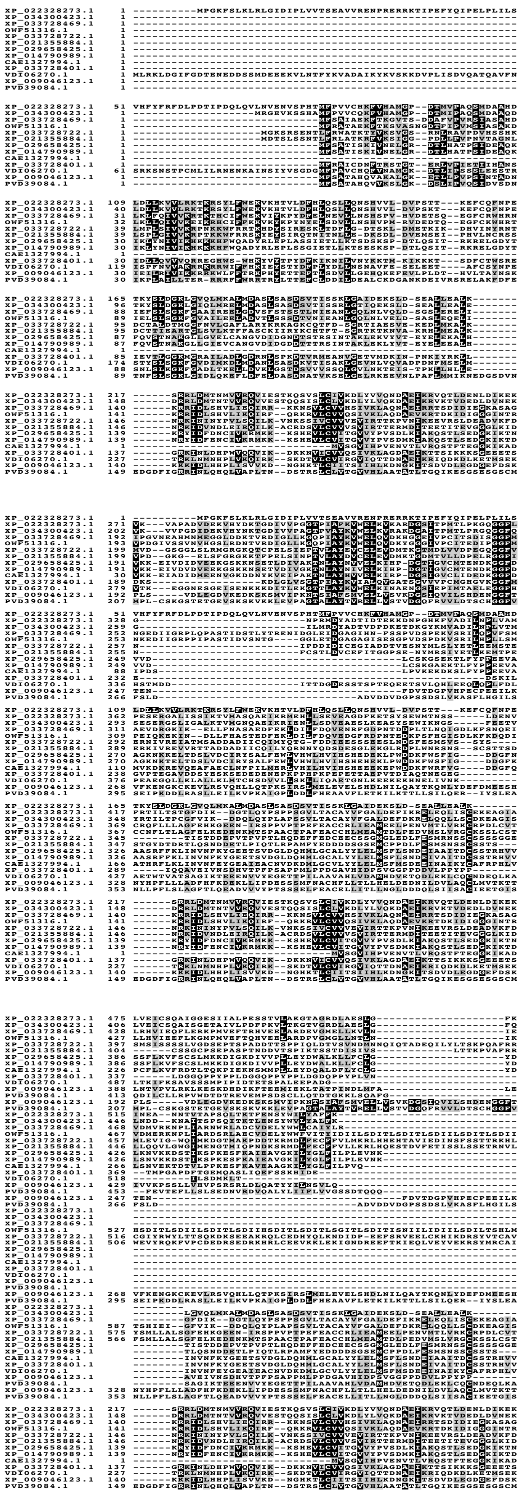

Fig. S13. Alignment of 13 Mollusca GSDM sequences.

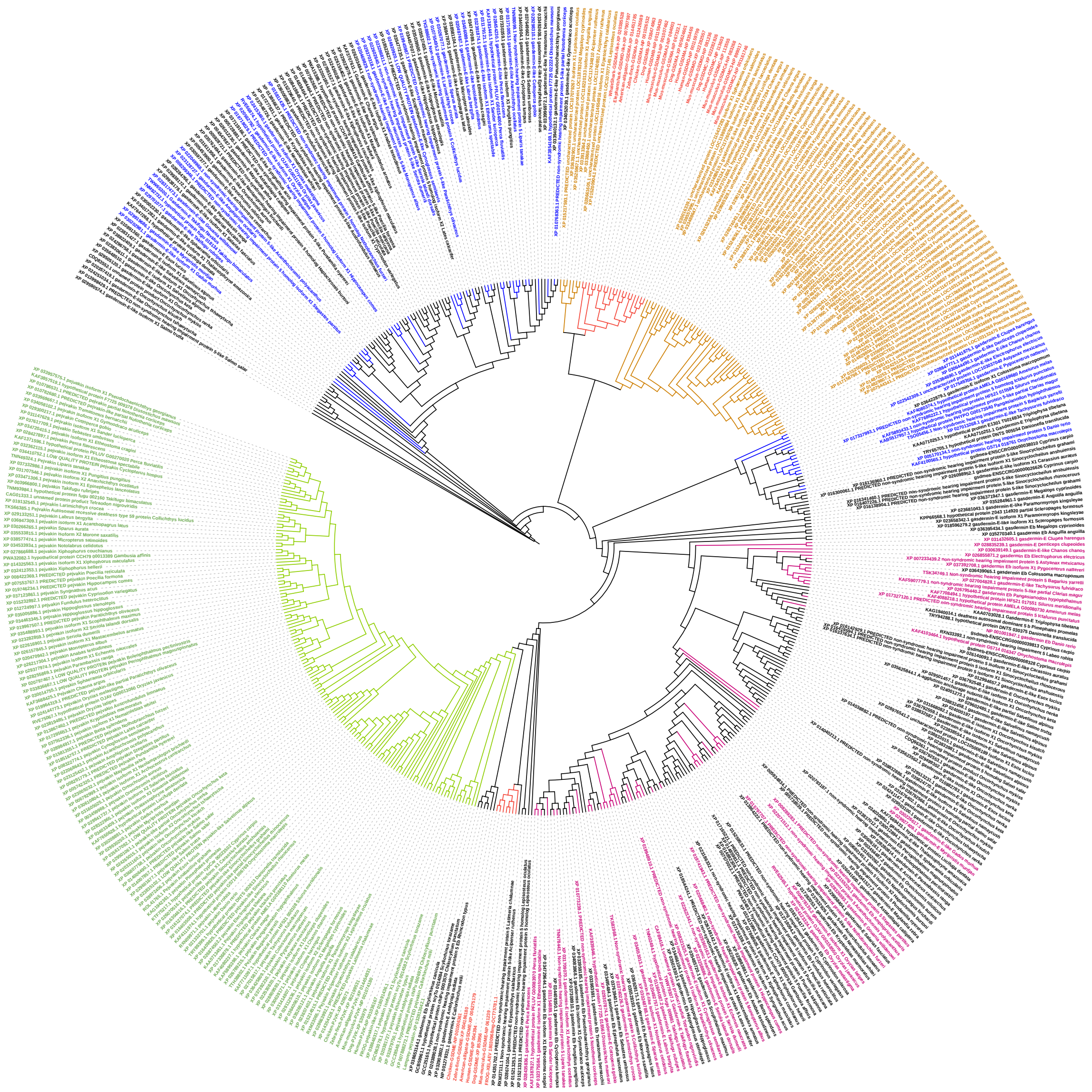

Fig. S14. The phylogenetic tree based on all identified Actinopterygii GSDME and GSDMF. The Actinopterygii GSDMEa and GSDMEb reference sequences from the NCBI Orthologs are marked in blue and magenta, respectively, and the newly identified GSDMEc are marked in yellow. The GSDME sequences from other vertebrates are marked in red. All GSDMF are marked in green. The bootstrap of 1000 and the method of JTT+F+R9 were applied.

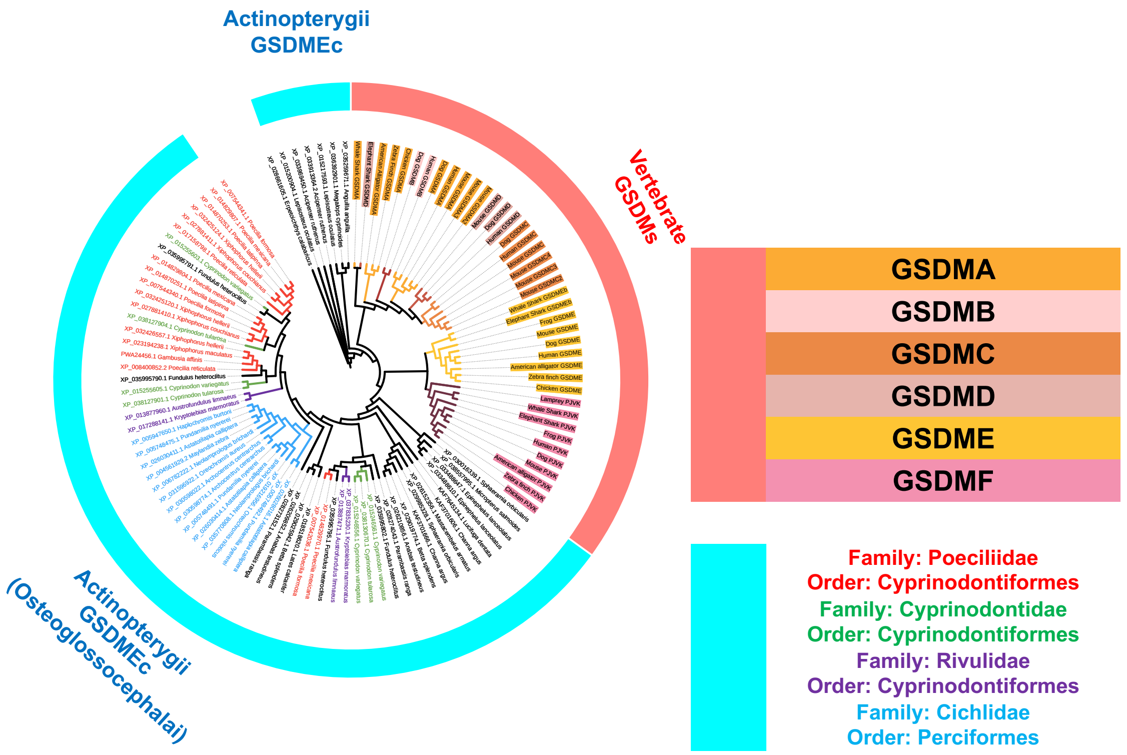

Fig. S15. The phylogenetic tree of Actinopterygii GSDMEc and vertebrate GSDMs. The Actinopterygii GSDMEc and the vertebrate GSDMs are indicated by blue and red, respectively, in the outer circle around the tree. Different Actinopterygii groups are indicated by different colors in the tree.

Table S1. The deduplicated sequences and accessions of the identified GSDM from every phylum.

	Accessions	Species
$GSDM_{in}$	XP_002109134.1	*Trichoplax adhaerens*
	XP_015769608.1	*Acropora digitifera*
	XP_029180327.1	*Acropora millepora*
	XP_031556688.1	*Actinia tenebrosa*
	XP_028402809.1	*Dendronephthya gigantea*
	KXJ08722.1	*Exaiptasia diaphana*
	XP_012557585.1	*Hydra vulgaris*
	EDO30320.1	*Nematostella vectensis*
	XP_020607257.1	*Orbicella faveolata*
	XP_027054110.1	*Pocillopora damicornis*
	PFX27013.1	*Stylophora pistillata*
	KAF6032630.1	*Bugula neritina*
	KAF6029209.1	*Bugula neritina*
	CAE1327994.1	*Sepia pharaonis*
	PVD39084.1	*Pomacea canaliculata*
	XP_033728722.1	*Pecten maximus*
	XP_033728469.1	*Pecten maximus*
	XP_033728401.1	*Pecten maximus*
	XP_029658425.1	*Octopus sinensis*
	XP_014790989.1	*Octopus bimaculoides*
	VDI06270.1	*Mytilus galloprovincialis*
	OWF51316.1	*Mizuhopecten yessoensis*
	XP_021355884.1	*Mizuhopecten yessoensis*
	XP_009046123.1	*Lottia gigantea*
	XP_022328273.1	*Crassostrea virginica*
	XP_034300423.1	*Crassostrea gigas*
	XP_013387688.1	*Lingula anatina*
	XP_030830373.1	*Strongylocentrotus purpuratus*
	XP_030830813.1	*Strongylocentrotus purpuratus*
	XP_006824139.1	*Saccoglossus kowalevskii*
	XP_002740828.1	*Saccoglossus kowalevskii*
	XP_035697721.1	*Branchiostoma floridae*
	XP_019614155.1	*Branchiostoma belcheri*
	98013420|pjvk	*Hofstenia miamia* [1]
	g3144.t1	*Phoronis australis* [2]
	g5835.t1	*Notospermus geniculatus* [2]
Ancient fish GSDM	XP_032879750.1	*Amblyraja radiata*
	XP_007888272.1	*Callorhinchus milii*
	GCC28549.1	*Chiloscyllium punctatum*
	XP_020381727.1	*Rhincodon typus*
	XP_038646317.1	*Scyliorhinus canicula*
	GCB63078.1	*Scyliorhinus torazame*
	XP_032903802.1	*Amblyraja radiata*
	NP_001279331.1	*Callorhinchus milii*
	GCC25165.1	*Chiloscyllium punctatum*
	XP_020389028.1	*Rhincodon typus*
	XP_038653144.1	*Scyliorhinus canicula*
	GCB59961.1	*Scyliorhinus torazame*
	XP_014351702.1	*Latimeria chalumnae*
	XP_014354128.1	*Latimeria chalumnae*
	XP_032813042.1	*Lamprey*
	XP_022049968.1	*Acanthochromis polyacanthus*

Actinopterygii GSDME	XP_022071512.1	*Acanthochromis polyacanthus*
	XP_036929171.1	*Acanthopagrus latus*
	XP_036947870.1	*Acanthopagrus latus*
	RXM37111.1	*Acipenser ruthenus*
	XP_033869450.1	*Acipenser ruthenus*
	XP_033913364.2	*Acipenser ruthenus*
	KAF4080374.1	*Ameiurus melas*
	KAF4088218.1	*Ameiurus melas*
	XP_023128727.1	*Amphiprion ocellaris*
	XP_023155220.1	*Amphiprion ocellaris*
	XP_026203484.1	*Anabas testudineus*
	XP_026209852.1	*Anabas testudineus*
	XP_026210856.1	*Anabas testudineus*
	XP_026226820.1	*Anabas testudineus*
	XP_031709070.1	*Anarrhichthys ocellatus*
	XP_031710053.1	*Anarrhichthys ocellatus*
	XP_035259671.1	*Anguilla anguilla*
	XP_035270340.1	*Anguilla anguilla*
	XP_035284961.1	*Anguilla anguilla*
	XP_030597377.1	*Archocentrus centrarchus*
	XP_030598022.1	*Archocentrus centrarchus*
	XP_030598774.1	*Archocentrus centrarchus*
	XP_030606161.1	*Archocentrus centrarchus*
	XP_026012230.1	*Astatotilapia calliptera*
	XP_026028016.1	*Astatotilapia calliptera*
	XP_026030411.1	*Astatotilapia calliptera*
	XP_026030414.1	*Astatotilapia calliptera*
	XP_026041600.1	*Astatotilapia calliptera*
	XP_007233439.2	*Astyanax mexicanus*
	XP_022542309.1	*Astyanax mexicanus*
	XP_013864222.1	*Austrofundulus limnaeus*
	XP_013866637.1	*Austrofundulus limnaeus*
	XP_013877960.1	*Austrofundulus limnaeus*
	XP_013887471.1	*Austrofundulus limnaeus*
	TSK34749.1	*Bagarius yarrelli*
	TSO05456.1	*Bagarius yarrelli*
	XP_028983881.1	*Betta splendens*
	XP_029019774.1	*Betta splendens*
	XP_029023078.1	*Betta splendens*
	XP_029025942.1	*Betta splendens*
	XP_020793187.1	*Boleophthalmus pectinirostris*
	XP_026098952.1	*Carassius auratus*
	XP_026140093.1	*Carassius auratus*
	KAF3701606.1	*Channa argus*
	KAF3701666.1	*Channa argus*
	KAF3707611.1	*Channa argus*
	XP_030639149.1	*Chanos chanos*
	XP_030644490.1	*Chanos chanos*
	KAF5893433.1	*Clarias magur*
	KAF5907779.1	*Clarias magur*
	XP_031432605.1	*Clupea harengus*
	XP_031441875.1	*Clupea harengus*
	TKS81968.1	*Collichthys lucidus*
	TKS88602.1	*Collichthys lucidus*
	XP_036422879.1	*Colossoma macropomum*
	XP_036439065.1	*Colossoma macropomum*
	XP_029298310.1	*Cottoperca gobio*
	XP_029307874.1	*Cottoperca gobio*
	XP_034401904.1	*Cyclopterus lumpus*

	XP_034409350.1	*Cyclopterus lumpus*
	XP_008321525.1	*Cynoglossus semilaevis*
	XP_024920920.1	*Cynoglossus semilaevis*
	XP_038125417.1	*Cyprinodon tularosa*
	XP_038127901.1	*Cyprinodon tularosa*
	XP_038127904.1	*Cyprinodon tularosa*
	XP_038130670.1	*Cyprinodon tularosa*
	XP_015229640.1	*Cyprinodon variegatus*
	XP_015246556.1	*Cyprinodon variegatus*
	XP_015246561.1	*Cyprinodon variegatus*
	XP_015255603.1	*Cyprinodon variegatus*
	XP_015255605.1	*Cyprinodon variegatus*
	XP_015259533.1	*Cyprinodon variegatus*
	ENSCCRG00000008328	*Cyprinus carpio*
	ENSCCRG00000026626	*Cyprinus carpio*
	ENSCCRG00000038010	*Cyprinus carpio*
	ENSCCRG00000039813	*Cyprinus carpio*
	NP_001001947.1	*Danio rerio*
	XP_005170134.1	*Danio rerio*
	TRY65705.1	*Danionella translucida*
	TRY94288.1	*Danionella translucida*
	XP_028835239.1	*Denticeps clupeoides*
	XP_028847771.1	*Denticeps clupeoides*
	KAF3839846.1	*Dissostichus mawsoni*
	KAF3854169.1	*Dissostichus mawsoni*
	XP_029360163.1	*Echeneis naucrates*
	XP_029370318.1	*Echeneis naucrates*
	XP_026855871.2	*Electrophorus electricus*
	XP_035384690.1	*Electrophorus electricus*
	XP_033488610.1	*Epinephelus lanceolatus*
	XP_033488647.1	*Epinephelus lanceolatus*
	XP_033492408.1	*Epinephelus lanceolatus*
	XP_033495484.1	*Epinephelus lanceolatus*
	XP_028661605.1	*Erpetoichthys calabaricus*
	XP_028674104.1	*Erpetoichthys calabaricus*
	XP_010882783.1	*Esox lucius*
	XP_010891360.1	*Esox lucius*
	XP_012994657.2	*Esox lucius*
	XP_028976541.2	*Esox lucius*
	XP_034729644.1	*Etheostoma cragini*
	XP_034747089.1	*Etheostoma cragini*
	XP_032375584.1	*Etheostoma spectabile*
	XP_032391874.1	*Etheostoma spectabile*
	XP_012718094.2	*Fundulus heteroclitus*
	XP_035995790.1	*Fundulus heteroclitus*
	XP_035995791.1	*Fundulus heteroclitus*
	XP_035995795.1	*Fundulus heteroclitus*
	XP_035995802.1	*Fundulus heteroclitus*
	XP_036000882.1	*Fundulus heteroclitus*
	XP_030202981.1	*Gadus morhua*
	XP_030226617.1	*Gadus morhua*
	PWA15389.1	*Gambusia affinis*
	PWA24456.1	*Gambusia affinis*
	PWA33483.1	*Gambusia affinis*
	XP_028317473.1	*Gouania willdenowi*
	XP_028326593.1	*Gouania willdenowi*
	XP_034052039.1	*Gymnodraco acuticeps*
	XP_034083868.1	*Gymnodraco acuticeps*
	XP_005928721.1	*Haplochromis burtoni*

	XP_005934834.1	*Haplochromis burtoni*
	XP_005947650.1	*Haplochromis burtoni*
	XP_019719029.1	*Hippocampus comes*
	XP_019741943.1	*Hippocampus comes*
	XP_034457007.1	*Hippoglossus hippoglossus*
	XP_034466104.1	*Hippoglossus hippoglossus*
	XP_035019303.1	*Hippoglossus stenolepis*
	XP_035039500.1	*Hippoglossus stenolepis*
	XP_017327120.1	*Ictalurus punctatus*
	XP_017337993.1	*Ictalurus punctatus*
	XP_017277831.1	*Kryptolebias marmoratus*
	XP_017282053.1	*Kryptolebias marmoratus*
	XP_017288141.1	*Kryptolebias marmoratus*
	XP_037835230.1	*Kryptolebias marmoratus*
	RXN33393.1	*Labeo rohita*
	XP_020492757.1	*Labrus bergylta*
	XP_020497487.1	*Labrus bergylta*
	XP_010740226.3	*Larimichthys crocea*
	XP_010755495.2	*Larimichthys crocea*
	XP_018518620.1	*Lates calcarifer*
	XP_018522627.1	*Lates calcarifer*
	XP_018544241.1	*Lates calcarifer*
	XP_015200904.1	*Lepisosteus oculatus*
	XP_015213253.1	*Lepisosteus oculatus*
	XP_015213331.1	*Lepisosteus oculatus*
	XP_015217593.1	*Lepisosteus oculatus*
	TNN78443.1	*Liparis tanakae*
	TNN88099.1	*Liparis tanakae*
	KAF7645134.1	*Lucifuga dentata*
	KAF7647268.1	*Lucifuga dentata*
	KAF7669433.1	*Lucifuga dentata*
	XP_026149142.1	*Mastacembelus armatus*
	XP_026152356.1	*Mastacembelus armatus*
	XP_026155402.1	*Mastacembelus armatus*
	XP_004541527.2	*Maylandia zebra*
	XP_004547887.2	*Maylandia zebra*
	XP_004561929.2	*Maylandia zebra*
	XP_036371947.1	*Megalops cyprinoides*
	XP_036392901.1	*Megalops cyprinoides*
	XP_036395434.1	*Megalops cyprinoides*
	XP_038557995.1	*Micropterus salmoides*
	XP_038581104.1	*Micropterus salmoides*
	XP_020468482.1	*Monopterus albus*
	XP_020469829.1	*Monopterus albus*
	XP_035528193.1	*Morone saxatilis*
	XP_035537455.1	*Morone saxatilis*
	XP_029919686.1	*Myripristis murdjan*
	XP_029927409.1	*Myripristis murdjan*
	XP_037532929.1	*Nematolebias whitei*
	XP_037548726.1	*Nematolebias whitei*
	XP_006782222.1	*Neolamprologus brichardi*
	XP_006801451.1	*Neolamprologus brichardi*
	XP_035762494.1	*Neolamprologus brichardi*
	XP_035770808.1	*Neolamprologus brichardi*
	XP_015797707.1	*Nothobranchius furzeri*
	XP_015811435.1	*Nothobranchius furzeri*
	XP_034553013.1	*Notolabrus celidotus*
	XP_034560894.1	*Notolabrus celidotus*
	XP_010768363.1	*Notothenia coriiceps*

	XP_010773239.1	*Notothenia coriiceps*
	XP_035623562.1	*Oncorhynchus keta*
	XP_035624770.1	*Oncorhynchus keta*
	XP_035625844.1	*Oncorhynchus keta*
	XP_035648510.1	*Oncorhynchus keta*
	XP_020357419.1	*Oncorhynchus kisutch*
	XP_031668082.1	*Oncorhynchus kisutch*
	XP_031676402.1	*Oncorhynchus kisutch*
	CDQ83552.1	*Oncorhynchus mykiss*
	CDQ96361.1	*Oncorhynchus mykiss*
	XP_036792548.1	*Oncorhynchus mykiss*
	XP_036792553.1	*Oncorhynchus mykiss*
	XP_036792559.1	*Oncorhynchus mykiss*
	XP_036792566.1	*Oncorhynchus mykiss*
	XP_029499302.1	*Oncorhynchus nerka*
	XP_029501457.1	*Oncorhynchus nerka*
	XP_029503100.1	*Oncorhynchus nerka*
	XP_029505120.1	*Oncorhynchus nerka*
	XP_029513231.1	*Oncorhynchus nerka*
	XP_024241147.1	*Oncorhynchus tshawytscha*
	XP_024251034.1	*Oncorhynchus tshawytscha*
	XP_024296758.1	*Oncorhynchus tshawytscha*
	KAF4100565.1	*Onychostoma macrolepis*
	KAF4103464.1	*Onychostoma macrolepis*
	XP_031596922.1	*Oreochromis aureus*
	XP_031602092.1	*Oreochromis aureus*
	XP_031611958.1	*Oreochromis aureus*
	XP_003454487.2	*Oreochromis niloticus*
	XP_005453900.1	*Oreochromis niloticus*
	XP_019216956.1	*Oreochromis niloticus*
	RVE62565.1	*Oryzias javanicus*
	RVE66874.1	*Oryzias javanicus*
	XP_004078135.2	*Oryzias latipes*
	XP_020562792.1	*Oryzias latipes*
	XP_024123324.1	*Oryzias melastigma*
	XP_024153460.1	*Oryzias melastigma*
	KAB5517957.1	*Pangasianodon hypophthalmus*
	XP_026795440.2	*Pangasianodon hypophthalmus*
	XP_019945987.1	*Paralichthys olivaceus*
	XP_019948910.1	*Paralichthys olivaceus*
	XP_028273152.1	*Parambassis ranga*
	XP_028274043.1	*Parambassis ranga*
	XP_028280814.1	*Parambassis ranga*
	XP_028284766.1	*Parambassis ranga*
	XP_023658342.1	*Paramormyrops kingsleyae*
	XP_023681043.1	*Paramormyrops kingsleyae*
	XP_028435836.1	*Perca flavescens*
	XP_028454230.1	*Perca flavescens*
	KAF1381440.1	*Perca fluviatilis*
	KAF1387827.1	*Perca fluviatilis*
	XP_033837012.1	*Periophthalmus magnuspinnatus*
	KAG1940014.1	*Pimephales promelas*
	XP_007542036.1	*Poecilia formosa*
	XP_007544340.1	*Poecilia formosa*
	XP_007544341.1	*Poecilia formosa*
	XP_007562681.1	*Poecilia formosa*
	XP_007570012.1	*Poecilia formosa*
	XP_014870251.1	*Poecilia latipinna*
	XP_014870253.1	*Poecilia latipinna*

	XP_014884464.1	*Poecilia latipinna*
	XP_014892308.1	*Poecilia latipinna*
	XP_014829804.1	*Poecilia mexicana*
	XP_014829807.1	*Poecilia mexicana*
	XP_014829970.1	*Poecilia mexicana*
	XP_014838454.1	*Poecilia mexicana*
	XP_014859811.1	*Poecilia mexicana*
	XP_008400852.2	*Poecilia reticulata*
	XP_008419845.1	*Poecilia reticulata*
	XP_017158798.1	*Poecilia reticulata*
	XP_017165221.1	*Poecilia reticulata*
	XP_033940132.1	*Pseudochaenichthys georgianus*
	XP_033959185.1	*Pseudochaenichthys georgianus*
	XP_005728881.1	*Pundamilia nyererei*
	XP_005738539.1	*Pundamilia nyererei*
	XP_005748475.1	*Pundamilia nyererei*
	XP_005748491.1	*Pundamilia nyererei*
	XP_005748492.1	*Pundamilia nyererei*
	XP_037308910.1	*Pungitius pungitius*
	XP_037330359.1	*Pungitius pungitius*
	XP_017549356.1	*Pygocentrus nattereri*
	XP_037392708.1	*Pygocentrus nattereri*
	XP_029938172.1	*Salarias fasciatus*
	XP_029938178.1	*Salarias fasciatus*
	XP_029958404.1	*Salarias fasciatus*
	XP_013998429.1	*Salmo salar*
	XP_014038882.1	*Salmo salar*
	XP_014040213.1	*Salmo salar*
	XP_029590574.1	*Salmo trutta*
	XP_029602485.1	*Salmo trutta*
	XP_023830611.1	*Salvelinus alpinus*
	XP_023831427.1	*Salvelinus alpinus*
	XP_023833981.1	*Salvelinus alpinus*
	XP_023838554.1	*Salvelinus alpinus*
	XP_024000197.1	*Salvelinus alpinus*
	XP_024001273.1	*Salvelinus alpinus*
	XP_038822409.1	*Salvelinus namaycush*
	XP_038832458.1	*Salvelinus namaycush*
	XP_038832587.1	*Salvelinus namaycush*
	XP_038832886.1	*Salvelinus namaycush*
	XP_038833015.1	*Salvelinus namaycush*
	XP_031154889.1	*Sander lucioperca*
	XP_031179121.1	*Sander lucioperca*
	KPP66568.1	*Scleropages formosus*
	XP_018596279.2	*Scleropages formosus*
	XP_035477235.1	*Scophthalmus maximus*
	XP_035485720.1	*Scophthalmus maximus*
	XP_037639401.1	*Sebastes umbrosus*
	XP_037649662.1	*Sebastes umbrosus*
	XP_022602640.1	*Seriola dumerili*
	XP_022621341.1	*Seriola dumerili*
	XP_023258886.1	*Seriola lalandi dorsalis*
	XP_023286811.1	*Seriola lalandi dorsalis*
	KAF7686222.1	*Silurus meridionalis*
	KAF7708494.1	*Silurus meridionalis*
	XP_016300061.1	*Sinocyclocheilus anshuiensis*
	XP_016337634.1	*Sinocyclocheilus anshuiensis*
	XP_016341460.1	*Sinocyclocheilus anshuiensis*
	XP_016136960.1	*Sinocyclocheilus grahami*

	XP_016138904.1	*Sinocyclocheilus grahami*
	XP_016142929.1	*Sinocyclocheilus grahami*
	XP_016407226.1	*Sinocyclocheilus rhinocerous*
	XP_016419594.1	*Sinocyclocheilus rhinocerous*
	XP_030248788.1	*Sparus aurata*
	XP_030267077.1	*Sparus aurata*
	XP_029985328.1	*Sphaeramia orbicularis*
	XP_030002330.1	*Sphaeramia orbicularis*
	XP_030012894.1	*Sphaeramia orbicularis*
	XP_030016339.1	*Sphaeramia orbicularis*
	XP_008289283.1	*Stegastes partitus*
	XP_008299346.1	*Stegastes partitus*
	XP_037119166.1	*Syngnathus acus*
	XP_037130338.1	*Syngnathus acus*
	XP_027004828.1	*Tachysurus fulvidraco*
	XP_027013268.1	*Tachysurus fulvidraco*
	TNM92732.1	*Takifugu bimaculatus*
	TNN02494.1	*Takifugu bimaculatus*
	TWW65300.1	*Takifugu flavidus*
	TWW79307.1	*Takifugu flavidus*
	XP_011604087.2	*Takifugu rubripes*
	XP_029701077.1	*Takifugu rubripes*
	CAF89418.1	*Tetraodon nigroviridis*
	XP_034017282.1	*Thalassophryne amazonica*
	XP_034030880.1	*Thalassophryne amazonica*
	XP_033983591.1	*Trematomus bernacchii*
	XP_033996972.1	*Trematomus bernacchii*
	KAA0703028.1	*Triplophysa tibetana*
	KAA0710251.1	*Triplophysa tibetana*
	KAA0710253.1	*Triplophysa tibetana*
	XP_027868605.1	*Xiphophorus couchianus*
	XP_027881410.1	*Xiphophorus couchianus*
	XP_027881411.1	*Xiphophorus couchianus*
	XP_027891527.1	*Xiphophorus couchianus*
	XP_032413990.1	*Xiphophorus hellerii*
	XP_032425120.1	*Xiphophorus hellerii*
	XP_032425124.1	*Xiphophorus hellerii*
	XP_032426557.1	*Xiphophorus hellerii*
	XP_032436028.1	*Xiphophorus hellerii*
	XP_023186332.1	*Xiphophorus maculatus*
	XP_023194238.1	*Xiphophorus maculatus*
	XP_023200180.1	*Xiphophorus maculatus*
Actinopterygii GSDMF	XP_022068643.1	*Acanthochromis polyacanthus*
	XP_036947309.1	*Acanthopagrus latus*
	RXM33065.1	*Acipenser ruthenus*
	KAF4080237.1	*Ameiurus melas*
	XP_023125432.1	*Amphiprion ocellaris*
	ROL41765.1	*Anabarilius grahami*
	XP_026217304.1	*Anabas testudineus*
	XP_031707546.1	*Anarrhichthys ocellatus*
	XP_035265121.1	*Anguilla anguilla*
	XP_030610804.1	*Archocentrus centrarchus*
	XP_022530820.1	*Astyanax mexicanus*
	XP_013867462.1	*Austrofundulus limnaeus*
	TTH38802.1	*Bagarius yarrelli*
	XP_028984917.1	*Betta splendens*
	XP_020797467.1	*Boleophthalmus pectinirostris*
	XP_026070516.1	*Carassius auratus*
	KAF3688425.1	*Channa argus*

	XP_030639171.1	*Chanos chanos*
	KAF5895398.1	*Clarias magur*
	XP_031414807.1	*Clupea harengus*
	TKS66385.1	*Collichthys lucidus*
	XP_036425809.1	*Colossoma macropomum*
	XP_029300217.1	*Cottoperca gobio*
	XP_034410752.1	*Cyclopterus lumpus*
	XP_008322774.1	*Cynoglossus semilaevis*
	XP_015232892.1	*Cyprinodon variegatus*
	KTF75217.1	*Cyprinus carpio*
	XP_021332701.1	*Danio rerio*
	TRY89399.1	*Danionella translucida*
	XP_028809593.1	*Denticeps clupeoides*
	KAF3857519.1	*Dissostichus mawsoni*
	XP_029377974.1	*Echeneis naucrates*
	XP_026874502.2	*Electrophorus electricus*
	XP_033471306.1	*Epinephelus lanceolatus*
	XP_028663992.1	*Erpetoichthys calabaricus*
	XP_028972406.2	*Esox lucius*
	XP_034720415.1	*Etheostoma cragini*
	XP_032362115.1	*Etheostoma spectabile*
	XP_012724997.1	*Fundulus heteroclitus*
	XP_030208891.1	*Gadus morhua*
	PWA32082.1	*Gambusia affinis*
	XP_028322965.1	*Gouania willdenowi*
	XP_034058102.1	*Gymnodraco acuticeps*
	XP_019746234.1	*Hippocampus comes*
	XP_034463345.1	*Hippoglossus hippoglossus*
	XP_035005886.1	*Hippoglossus stenolepis*
	XP_017336878.1	*Ictalurus punctatus*
	XP_017295653.1	*Kryptolebias marmoratus*
	RXN09971.1	*Labeo rohita*
	XP_029133251.1	*Labrus bergylta*
	XP_019132545.1	*Larimichthys crocea*
	XP_018516757.1	*Lates calcarifer*
	XP_015214515.1	*Lepisosteus oculatus*
	TNN49324.1	*Liparis tanakae*
	KAF7650628.1	*Lucifuga dentata*
	XP_026157845.1	*Mastacembelus armatus*
	XP_023009232.1	*Maylandia zebra*
	XP_036400610.1	*Megalops cyprinoides*
	XP_038577474.1	*Micropterus salmoides*
	XP_020470942.1	*Monopterus albus*
	XP_035533815.1	*Morone saxatilis*
	XP_029930250.1	*Myripristis murdjan*
	XP_037552336.1	*Nematolebias whitei*
	XP_006792999.1	*Neolamprologus brichardi*
	XP_015813551.1	*Nothobranchius furzeri*
	XP_034533934.1	*Notolabrus celidotus*
	XP_010782680.1	*Notothenia coriiceps*
	XP_010786531.1	*Notothenia coriiceps*
	XP_035617092.1	*Oncorhynchus keta*
	XP_020331160.2	*Oncorhynchus kisutch*
	XP_036807758.1	*Oncorhynchus mykiss*
	XP_029501061.1	*Oncorhynchus nerka*
	XP_024265015.1	*Oncorhynchus tshawytscha*
	KAF4111974.1	*Onychostoma macrolepis*
	XP_031605025.1	*Oreochromis aureus*
	XP_003459969.1	*Oreochromis niloticus*

	RVE75067.1	*Oryzias javanicus*
	XP_023819485.1	*Oryzias latipes*
	XP_024144773.1	*Oryzias melastigma*
	XP_026789215.1	*Pangasianodon hypophthalmus*
	XP_019964319.1	*Paralichthys olivaceus*
	XP_019967507.1	*Paralichthys olivaceus*
	XP_028255869.1	*Parambassis ranga*
	XP_023657402.1	*Paramormyrops kingsleyae*
	XP_028427897.1	*Perca flavescens*
	KAF1371596.1	*Perca fluviatilis*
	XP_033836667.1	*Periophthalmus magnuspinnatus*
	KAG1942361.1	*Pimephales promelas*
	XP_007553767.1	*Poecilia formosa*
	XP_008422369.1	*Poecilia reticulata*
	XP_033957575.1	*Pseudochaenichthys georgianus*
	XP_005742320.1	*Pundamilia nyererei*
	XP_037332986.1	*Pungitius pungitius*
	XP_037392230.1	*Pygocentrus nattereri*
	XP_029941737.1	*Salarias fasciatus*
	XP_014006490.1	*Salmo salar*
	XP_014007857.1	*Salmo salar*
	XP_029601677.1	*Salmo trutta*
	XP_023835102.1	*Salvelinus alpinus*
	XP_031147629.1	*Sander lucioperca*
	XP_029112536.1	*Scleropages formosus*
	XP_035488993.1	*Scophthalmus maximus*
	XP_037617709.1	*Sebastes umbrosus*
	XP_022616901.1	*Seriola dumerili*
	XP_023282859.1	*Seriola lalandi dorsalis*
	KAF7711097.1	*Silurus meridionalis*
	XP_016304616.1	*Sinocyclocheilus anshuiensis*
	XP_016127074.1	*Sinocyclocheilus grahami*
	XP_016403508.1	*Sinocyclocheilus rhinocerous*
	XP_016409825.1	*Sinocyclocheilus rhinocerous*
	XP_030266265.1	*Sparus aurata*
	XP_030014755.1	*Sphaeramia orbicularis*
	XP_008291779.1	*Stegastes partitus*
	XP_037123861.1	*Syngnathus acus*
	XP_027015817.1	*Tachysurus fulvidraco*
	TNM93984.1	*Takifugu bimaculatus*
	XP_003966800.1	*Takifugu rubripes*
	CAG01333.1	*Tetraodon nigroviridis*
	XP_034033409.1	*Thalassophryne amazonica*
	XP_033969667.1	*Trematomus bernacchii*
	KAA0713451.1	*Triplophysa tibetana*
	XP_027866688.1	*Xiphophorus couchianus*
	XP_032412353.1	*Xiphophorus hellerii*
	XP_014325563.1	*Xiphophorus maculatus*
Amphibia GSDMA	XP_030077860	*Microcaecilia unicolor*
	XP_029428328.1	*Rhinatrema bivittatum*
	XP_033774377.1	*Geotrypetes seraphini*
	XP_033774373.1	*Geotrypetes seraphini*
Amphibia GSDME	XP_033786715.1	*Geotrypetes seraphini*
	XP_030057917.1	*Microcaecilia unicolor*
	XP_018431097.1	*Nanorana parkeri*
	XP_029444983.1	*Rhinatrema bivittatum*
	OCT75781.1	*Xenopus laevis*
	XP_002933445.2	*Xenopus tropicalis*
	XP_033801535.1	*Geotrypetes seraphini*

Amphibia GSDMF	XP_030065796.1	*Microcaecilia unicolor*
	XP_018417121.1	*Nanorana parkeri*
	XP_029461755.1	*Rhinatrema bivittatum*
	XP_018091436.1	*Xenopus laevis*
	XP_017953002.1	*Xenopus tropicalis*
Reptilia GSDME	XP_027689547.1	*Chelonia mydas*
	XP_032629742.1	*Chelonoidis abingdonii*
	XP_023962559.1	*Chrysemys picta bellii*
	XP_038247024.1	*Dermochelys coriacea*
	XP_030406661.1	*Gopherus evgoodei*
	XP_006138433.1	*Pelodiscus sinensis*
	TFK07372.1	*Platysternon megacephalum*
	XP_029766858.1	*Terrapene carolina triunguis*
	XP_034616541.1	*Trachemys scripta elegans*
	XP_003222077	*Anolis carolinensis*
	XP_006275179	*Alligator mississippiensis*
	XP_025063881	*Alligator sinensis*
Reptilia GSDMF	XP_007069837.1	*Chelonia mydas*
	XP_032624383.1	*Chelonoidis abingdonii*
	XP_005300562.1	*Chrysemys picta bellii*
	XP_005293660.1	*Chrysemys picta bellii*
	XP_038276825.1	*Dermochelys coriacea*
	XP_030435617.1	*Gopherus evgoodei*
	XP_025038578.1	*Pelodiscus sinensis*
	TFK14555.1	*Platysternon megacephalum*
	XP_024054274.1	*Terrapene carolina triunguis*
	XP_034641386.1	*Trachemys scripta elegans*
	XP_003225734	*Anolis carolinensis*
	XP_014454831	*Alligator mississippiensis*
	XP_006034600	*Alligator sinensis*
Aves GSDME	XP_009076145.1	*Acanthisitta chloris*
	NWZ63163.1	*Acrocephalus arundinaceus*
	NWH96391.1	*Aegithalos caudatus*
	NWX13329.1	*Aegotheles bennettii*
	NWZ04843.1	*Agelaius phoeniceus*
	NXQ28983.1	*Alaudala cheleensis*
	NXC53029.1	*Aleadryas rufinucha*
	NXL93202.1	*Alectura lathami*
	NXW88766.1	*Alopecoenas beccarii*
	KQK78344.1	*Amazona aestiva*
	NXK76289.1	*Amazona guildingii*
	XP_005020592.3	*Anas platyrhynchos*
	NXC72046.1	*Anhinga anhinga*
	NXT88897.1	*Anhinga rufa*
	XP_013027320.1	*Anser cygnoides domesticus*
	NXI67114.1	*Anseranas semipalmata*
	NXQ57040.1	*Anthoscopus minutus*
	XP_010165530.1	*Antrostomus carolinensis*
	XP_009863572.1	*Apaloderma vittatum*
	NWY09896.1	*Aphelocoma coerulescens*
	XP_009280316.1	*Aptenodytes forsteri*
	KAF1667318.1	*Aptenodytes patagonicus*
	XP_013796819.1	*Apteryx mantelli mantelli*
	XP_025922589.1	*Apteryx rowi*
	XP_029866133.1	*Aquila chrysaetos chrysaetos*
	NXO61008.1	*Aramus guarauna*
	NXE23676.1	*Ardeotis kori*
	NXK21040.1	*Arenaria interpres*
	NWZ27074.1	*Asarcornis scutulata*

	XP_026697503.1	*Athene cunicularia*
	NXV73925.1	*Atlantisia rogersi*
	NXY15092.1	*Atrichornis clamosus*
	XP_032037136.1	*Aythya fuligula*
	NXS48873.1	*Balaeniceps rex*
	XP_010297344.1	*Balearica regulorum gibbericeps*
	XP_010304991.1	*Balearica regulorum gibbericeps*
	NXG80359.1	*Baryphthengus martii*
	NXN84994.1	*Bombycilla garrulus*
	NWZ37985.1	*Brachypodius atriceps*
	NXS52247.1	*Brachypteracias leptosomus*
	NXH15162.1	*Bucco capensis*
	XP_010131040.1	*Buceros rhinoceros silvestris*
	NWR61062.1	*Bucorvus abyssinicus*
	NXT99250.1	*Buphagus erythrorhynchus*
	NWQ98869.1	*Burhinus bistriatus*
	NXE64188.1	*Calcarius ornatus*
	XP_014793123.1	*Calidris pugnax*
	NXY53229.1	*Callaeas wilsoni*
	OXB56210.1	*Callipepla squamata*
	NWX07373.1	*Caloenas nicobarica*
	NXV94332.1	*Calonectris borealis*
	XP_030301104.1	*Calypte anna*
	NWI48919.1	*Calyptomena viridis*
	XP_030799876.1	*Camarhynchus parvulus*
	NXC31051.1	*Campylorhamphus procurvoides*
	NWT22682.1	*Cardinalis cardinalis*
	XP_009702494.1	*Cariama cristata*
	NXE45491.1	*Casuarius casuarius*
	KFP51075.1	*Cathartes aura*
	NXQ44923.1	*Catharus fuscescens*
	XP_032932115.1	*Catharus ustulatus*
	NXX91727.1	*Centropus bengalensis*
	NWR72566.1	*Centropus unirufus*
	NWU15074.1	*Cephalopterus ornatus*
	NXV16249.1	*Cepphus grylle*
	NXU97156.1	*Cettia cetti*
	NXY42496.1	*Ceuthmochares aereus*
	NXT61916.1	*Chaetops frenatus*
	NXD96142.1	*Chaetorhynchus papuensis*
	XP_010005878.1	*Chaetura pelagica*
	XP_009877840.1	*Charadrius vociferus*
	NXK47508.1	*Chauna torquata*
	NWY52550.1	*Chionis minor*
	XP_032566906.1	*Chiroxiphia lanceolata*
	XP_010116360.1	*Chlamydotis macqueenii*
	RLW11495.1	*Chloebia gouldiae*
	NXI58809.1	*Chloroceryle aenea*
	NXP60531.1	*Chloropsis cyanopogon*
	NWH33497.1	*Chloropsis hardwickii*
	NWT43555.1	*Chroicocephalus maculipennis*
	NWS56740.1	*Chunga burmeisteri*
	NXF64154.1	*Ciccaba nigrolineata*
	NXJ36016.1	*Ciconia maguari*
	NXR18913.1	*Cinclus mexicanus*
	NXW21942.1	*Circaetus pectoralis*
	NXO28324.1	*Cisticola juncidis*
	NWW76503.1	*Climacteris rufus*
	NXB07157.1	*Cnemophilus loriae*

	NXE80765.1	*Cochlearius cochlearius*
	OXB74559.1	*Colinus virginianus*
	XP_010199052.1	*Colius striatus*
	PKK32165.1	*Columba livia*
	NWQ72991.1	*Columbina picui*
	NXD35878.1	*Copsychus sechellarum*
	XP_027495202.1	*Corapipo altera*
	XP_017586668.1	*Corvus brachyrhynchos*
	XP_010397332.1	*Corvus cornix cornix*
	XP_031965410.1	*Corvus moneduloides*
	NXJ96696.1	*Corythaixoides concolor*
	XP_015709251.1	*Coturnix japonica*
	NWS66524.1	*Crotophaga sulcirostris*
	NWI08280.1	*Crypturellus soui*
	NWJ05274.1	*Crypturellus undulatus*
	XP_009561166.1	*Cuculus canorus*
	XP_023776255.1	*Cyanistes caeruleus*
	XP_035418294.1	*Cygnus atratus*
	NWV50539.1	*Daphoenositta chrysoptera*
	NWV84597.1	*Dasyornis broadbenti*
	NXH45857.1	*Dicaeum eximium*
	NXJ22819.1	*Dicrurus megarhynchus*
	NXB77463.1	*Donacobius atricapilla*
	XP_025959583.1	*Dromaius novaehollandiae*
	NWU56626.1	*Dromas ardeola*
	NXU39075.1	*Drymodes brunneopygia*
	XP_009906998.1	*Dryobates pubescens*
	NWI74330.1	*Dryoscopus gambensis*
	NXH84678.1	*Edolisoma coerulescens*
	XP_035749981.1	*Egretta garzetta*
	NXD25299.1	*Elachura formosa*
	NWR16375.1	*Emberiza fucata*
	XP_027751877.1	*Empidonax traillii*
	NXD61783.1	*Eolophus roseicapilla*
	NWY72233.1	*Erithacus rubecula*
	NXS80204.1	*Erpornis zantholeuca*
	NWT50531.1	*Erythrocercus mccallii*
	NXF94231.1	*Eubucco bourcierii*
	NXA35131.1	*Eudromia elegans*
	KAF1502516.1	*Eudyptula minor*
	NXB31810.1	*Eulacestoma nigropectus*
	XP_010144987.1	*Eurypyga helias*
	NXW65146.1	*Eurystomus gularis*
	XP_027670553.1	*Falco cherrug*
	XP_027642247.1	*Falco peregrinus*
	NWW28126.1	*Falcunculus frontatus*
	XP_005041047.1	*Ficedula albicollis*
	NXK89341.1	*Formicarius rufipectus*
	NWH53761.1	*Fregata magnificens*
	NXV99994.1	*Fregetta grallaria*
	XP_009582421.1	*Fulmarus glacialis*
	NWR95182.1	*Furnarius figulus*
	NXI46395.1	*Galbula dea*
	NP_001006361.1	*Gallus gallus*
	XP_009808686.1	*Gavia stellata*
	NWH60538.1	*Geococcyx californianus*
	XP_030913538.1	*Geospiza fortis*
	NXY70906.1	*Glareola pratincola*
	NXL35788.1	*Glaucidium brasilianum*

	NXG24948.1	*Grallaria varia*
	NWV43653.1	*Grantiella picta*
	NWH17386.1	*Grus americana*
	NXM42385.1	*Gymnorhina tibicen*
	NXD83779.1	*Halcyon senegalensis*
	XP_009928905.1	*Haliaeetus albicilla*
	NXP48887.1	*Heliornis fulica*
	NXG56750.1	*Hemiprocne comata*
	NXK11147.1	*Herpetotheres cachinnans*
	NXN63270.1	*Himantopus himantopus*
	NXR49635.1	*Hippolais icterina*
	NXW67737.1	*Hirundo rustica*
	RMC05122.1	*Hirundo rustica rustica*
	NXU65989.1	*Horornis vulcanius*
	NWU43202.1	*Hylia prasina*
	NXR87819.1	*Hypocryptadius cinnamomeus*
	NXA19240.1	*Ibidorhyncha struthersii*
	NWW58082.1	*Ifrita kowaldi*
	NXM53488.1	*Illadopsis cleaveri*
	NXN16725.1	*Indicator maculatus*
	NXI13147.1	*Irena cyanogastra*
	NXS92551.1	*Jacana jacana*
	NWT82582.1	*Lanius ludovicianus*
	NXX04593.1	*Larus smithsonianus*
	NXP33182.1	*Leiothrix lutea*
	XP_017679311.1	*Lepidothrix coronata*
	NXL73245.1	*Leptocoma aspasia*
	XP_009960492.1	*Leptosomus discolor*
	NXB46077.1	*Leucopsar rothschildi*
	PKU29845.1	*Limosa lapponica baueri*
	PKU30542.1	*Limosa lapponica baueri*
	NXO39052.1	*Locustella ochotensis*
	XP_021393912.1	*Lonchura striata domestica*
	NXE11254.1	*Lophotis ruficrista*
	NWY96568.1	*Loxia curvirostra*
	NWV98181.1	*Machaerirhynchus nigripectus*
	NWV68412.1	*Malurus elegans*
	XP_017930682.2	*Manacus vitellinus*
	KAF1495095.1	*Megadyptes antipodes antipodes*
	NXA86679.1	*Melanocharis versteri*
	XP_003207182.1	*Meleagris gallopavo*
	XP_030909397.2	*Melopsittacus undulatus*
	NWQ53888.1	*Melospiza melodia*
	KAF2983348.1	*Melospiza melodia maxima*
	NXE91737.1	*Menura novaehollandiae*
	XP_008933812.1	*Merops nubicus*
	NXL07146.1	*Mesembrinibis cayennensis*
	XP_010183520.1	*Mesitornis unicolor*
	NWS93441.1	*Mionectes macconnelli*
	NXA67296.1	*Mohoua ochrocephala*
	XP_036235014.1	*Molothrus ater*
	NWR97104.1	*Motacilla alba*
	XP_037979427.1	*Motacilla alba alba*
	NXH35697.1	*Myiagra hebetior*
	NXS16208.1	*Mystacornis crossleyi*
	NXS08731.1	*Neodrepanis coruscans*
	XP_027531052.1	*Neopelma chrysocephalum*
	NWQ68931.1	*Neopipo cinnamomea*
	NXA02017.1	*Nesospiza acunhae*

	XP_010009916.1	*Nestor notabilis*
	NXX33261.1	*Nicator chloris*
	XP_009464781.1	*Nipponia nippon*
	NXA52559.1	*Nothocercus julius*
	NXD12366.1	*Nothocercus nigrocapillus*
	NWX88081.1	*Nothoprocta pentlandii*
	XP_025889163.1	*Nothoprocta perdicaria*
	NWX31257.1	*Notiomystis cincta*
	XP_021243118.1	*Numida meleagris*
	NXF35781.1	*Nyctibius bracteatus*
	NXQ90685.1	*Nyctibius grandis*
	NXN33259.1	*Nycticryphes semicollaris*
	NXW49708.1	*Nyctiprogne leucopyga*
	NXF49017.1	*Oceanites oceanicus*
	NXH76941.1	*Oceanodroma tethys*
	NXJ16009.1	*Odontophorus gujanensis*
	NWU74723.1	*Onychorhynchus coronatus*
	XP_009939657.1	*Opisthocomus hoazin*
	NWW07329.1	*Oreocharis arfaki*
	NXU73288.1	*Oreotrochilus melanogaster*
	NWV21137.1	*Origma solitaria*
	NXO10429.1	*Oriolus oriolus*
	NXC10976.1	*Orthonyx spaldingii*
	NXR99776.1	*Oxylabes madagascariensis*
	XP_035172164.1	*Oxyura jamaicensis*
	NXH99567.1	*Pachycephala philippinensis*
	NWS12954.1	*Pachyramphus minor*
	NXS67881.1	*Pandion haliaetus*
	NWW41259.1	*Panurus biarmicus*
	NXU14602.1	*Pardalotus punctatus*
	XP_015473232.2	*Parus major*
	NXP84062.1	*Passerina amoena*
	OPJ81947.1	*Patagioenas fasciata monilis*
	NWW54232.1	*Pedionomus torquatus*
	NXT42547.1	*Pelecanoides urinatrix*
	XP_009489562.1	*Pelecanus crispus*
	NXC43583.1	*Penelope pileata*
	NXQ13184.1	*Peucedramus taeniatus*
	XP_010285141.1	*Phaethon lepturus*
	XP_010288632.1	*Phaethon lepturus*
	NXW29442.1	*Phaetusa simplex*
	NXO65083.1	*Phainopepla nitens*
	XP_009506330.1	*Phalacrocorax carbo*
	XP_031445980.1	*Phasianus colchicus*
	NWY29208.1	*Pheucticus melanocephalus*
	KFQ87781.1	*Phoenicopterus ruber ruber*
	NWH77535.1	*Piaya cayana*
	NWI44660.1	*Picathartes gymnocephalus*
	XP_027594244.1	*Pipra filicauda*
	NXK34734.1	*Piprites chloris*
	NWI94583.1	*Pitta sordida*
	NWU21187.1	*Platysteira castanea*
	NXM13442.1	*Ploceus nigricollis*
	NXT46700.1	*Pluvianellus socialis*
	NXX14623.1	*Podargus strigoides*
	KFZ62372.1	*Podiceps cristatus*
	NXL47137.1	*Podilymbus podiceps*
	NWZ76813.1	*Poecile atricapillus*
	NWS33054.1	*Polioptila caerulea*

	NXS27631.1	*Pomatostomus ruficeps*
	NWS38550.1	*Probosciger aterrimus*
	NWX58673.1	*Promerops cafer*
	NWT73450.1	*Prunella himalayana*
	XP_014104276.1	*Pseudopodoces humilis*
	NXG41079.1	*Psilopogon haemacephalus*
	NXI89846.1	*Psophia crepitans*
	NWU65709.1	*Pterocles burchelli*
	XP_010072951.1	*Pterocles gutturalis*
	NXY01458.1	*Pteruthius melanotis*
	NWV02851.1	*Ptilonorhynchus violaceus*
	NXE33572.1	*Ptilorrhoa leucosticta*
	NXR77211.1	*Pycnonotus jocosus*
	XP_009324004.1	*Pygoscelis adeliae*
	KAF1459802.1	*Pygoscelis antarcticus*
	KAF1441869.1	*Pygoscelis papua*
	NXP77708.1	*Ramphastos sulfuratus*
	NWR51767.1	*Regulus satrapa*
	NXH53164.1	*Rhabdornis inornatus*
	NXR64493.1	*Rhadina sibilatrix*
	NXB21515.1	*Rhagologus leucostigma*
	NWY84340.1	*Rhegmatorhina hoffmannsi*
	NXN90827.1	*Rhinopomastus cyanomelas*
	NXN45809.1	*Rhinoptilus africanus*
	NXI77950.1	*Rhipidura dahli*
	NXF21021.1	*Rhodinocichla rosea*
	NWW88516.1	*Rhynochetos jubatus*
	NXV27807.1	*Rissa tridactyla*
	NXJ61473.1	*Rostratula benghalensis*
	NXN53880.1	*Rynchops niger*
	NXQ96106.1	*Sagittarius serpentarius*
	NXG03141.1	*Sakesphorus luctuosus*
	NXA09568.1	*Sapayoa aenigma*
	NXF78687.1	*Sclerurus mexicanus*
	NXX52263.1	*Scopus umbretta*
	NXP17743.1	*Scytalopus superciliaris*
	NXR07323.1	*Semnornis frantzii*
	NXM73574.1	*Serilophus lunatus*
	XP_030090516.1	*Serinus canaria*
	NXL15596.1	*Setophaga kirtlandii*
	NWR05347.1	*Sinosuthora webbiana*
	NXO77688.1	*Sitta europaea*
	NXF00767.1	*Smithornis capensis*
	KAF1406329.1	*Spheniscus humboldti*
	NXJ51053.1	*Spizaetus tyrannus*
	NWX40938.1	*Steatornis caripensis*
	NXG92549.1	*Stercorarius parasiticus*
	NXI29108.1	*Sterrhoptilus dennistouni*
	XP_030345602.1	*Strigops habroptila*
	NXB58295.1	*Struthidea cinerea*
	XP_009674747.1	*Struthio camelus australis*
	XP_014749815.1	*Sturnus vulgaris*
	NWI30015.1	*Sula dactylatra*
	NWY41975.1	*Sylvia atricapilla*
	NXN03313.1	*Sylvia borin*
	NXK62015.1	*Sylvietta virens*
	NXT23596.1	*Syrrhaptes paradoxus*
	NWR27611.1	*Tachuris rubrigastra*
	XP_004186180.4	*Taeniopygia guttata*

	XP_009980061.1	*Tauraco erythrolophus*
	XP_009983371.1	*Tauraco erythrolophus*
	NXU28240.1	*Thalassarche chlororhynchos*
	NXP12645.1	*Thinocorus orbignyianus*
	NXA79430.1	*Thryothorus ludovicianus*
	NWH97270.1	*Tichodroma muraria*
	XP_010220413.1	*Tinamus guttatus*
	NWI63968.1	*Todus mexicanus*
	NWS90606.1	*Toxostoma redivivum*
	NXX49156.1	*Tricholaema leucomelas*
	NXJ74683.1	*Trogon melanurus*
	KAF4799219.1	*Turdus rufiventris*
	NXU56534.1	*Turnix velox*
	NXL99838.1	*Tyrannus savana*
	KFV45587.1	*Tyto alba*
	XP_009960564.2	*Tyto alba alba*
	NWU95265.1	*Upupa epops*
	NXV48938.1	*Uria aalge*
	NXX82162.1	*Urocolius indicus*
	NWT99995.1	*Urocynchramus pylzowi*
	NXB89577.1	*Vidua chalybeata*
	NWT20729.1	*Vireo altiloquus*
	NXU91921.1	*Xiphorhynchus elegans*
	NXT82065.1	*Zapornia atra*
	XP_005480924.2	*Zonotrichia albicollis*
	TRZ18442.1	*Zosterops borbonicus*
	NXR30653.1	*Zosterops hypoxanthus*
Aves GSDMF	XP_009082354.1	*Acanthisitta chloris*
	NWZ70089.1	*Acrocephalus arundinaceus*
	NWH92666.1	*Aegithalos caudatus*
	NWX17420.1	*Aegotheles bennettii*
	NWZ15463.1	*Agelaius phoeniceus*
	NXQ33592.1	*Alaudala cheleensis*
	NWX73342.1	*Alca torda*
	NXL83846.1	*Alectura lathami*
	NXW93374.1	*Alopecoenas beccarii*
	KQL59988.1	*Amazona aestiva*
	XP_038038033.1	*Anas platyrhynchos*
	NXC70081.1	*Anhinga anhinga*
	XP_013027050.1	*Anser cygnoides domesticus*
	NXI74064.1	*Anseranas semipalmata*
	NXQ51745.1	*Anthoscopus minutus*
	XP_010173029.1	*Antrostomus carolinensis*
	XP_009868885.1	*Apaloderma vittatum*
	NWY11331.1	*Aphelocoma coerulescens*
	XP_009276888.1	*Aptenodytes forsteri*
	XP_013816192.1	*Apteryx mantelli mantelli*
	XP_029874974.1	*Aquila chrysaetos chrysaetos*
	NXO55173.1	*Aramus guarauna*
	NXE21863.1	*Ardeotis kori*
	NWZ19528.1	*Asarcornis scutulata*
	XP_026708820.1	*Athene cunicularia*
	NXV81190.1	*Atlantisia rogersi*
	NXY25541.1	*Atrichornis clamosus*
	XP_032046572.1	*Aythya fuligula*
	NXS46721.1	*Balaeniceps rex*
	XP_010309352.1	*Balearica regulorum gibbericeps*
	POI30071.1	*Bambusicola thoracicus*
	NXG78726.1	*Baryphthengus martii*

	NXN80528.1	*Bombycilla garrulus*
	NWZ33453.1	*Brachypodius atriceps*
	NXH14886.1	*Bucco capensis*
	XP_010133399.1	*Buceros rhinoceros silvestris*
	NWR58911.1	*Bucorvus abyssinicus*
	NXU00957.1	*Buphagus erythrorhynchus*
	NWQ94523.1	*Burhinus bistriatus*
	NXE58408.1	*Calcarius ornatus*
	XP_014809935.1	*Calidris pugnax*
	NXY52690.1	*Callaeas wilsoni*
	OXB61715.1	*Callipepla squamata*
	NWW99470.1	*Caloenas nicobarica*
	NXV98498.1	*Calonectris borealis*
	XP_008503297.1	*Calypte anna*
	NWI57091.1	*Calyptomena viridis*
	NWT31405.1	*Cardinalis cardinalis*
	XP_009694144.1	*Cariama cristata*
	NXE55278.1	*Casuarius casuarius*
	XP_032920040.1	*Catharus ustulatus*
	NXX88455.1	*Centropus bengalensis*
	NWR69469.1	*Centropus unirufus*
	NWU16096.1	*Cephalopterus ornatus*
	NXV19602.1	*Cepphus grylle*
	NXC80023.1	*Cercotrichas coryphoeus*
	NXO95528.1	*Certhia brachydactyla*
	NXC95101.1	*Certhia familiaris*
	NXV04722.1	*Cettia cetti*
	NXY40901.1	*Ceuthmochares aereus*
	NXT71324.1	*Chaetops frenatus*
	NXD91737.1	*Chaetorhynchus papuensis*
	XP_009994488.1	*Chaetura pelagica*
	XP_009883913.1	*Charadrius vociferus*
	NXK56042.1	*Chauna torquata*
	NWY54905.1	*Chionis minor*
	XP_032549672.1	*Chiroxiphia lanceolata*
	XP_010123753.1	*Chlamydotis macqueenii*
	RLW00945.1	*Chloebia gouldiae*
	NXI56937.1	*Chloroceryle aenea*
	NXP62306.1	*Chloropsis cyanopogon*
	NWH30730.1	*Chloropsis hardwickii*
	NXL56939.1	*Chordeiles acutipennis*
	NWT41626.1	*Chroicocephalus maculipennis*
	NWS59032.1	*Chunga burmeisteri*
	NXF58944.1	*Ciccaba nigrolineata*
	NXJ32057.1	*Ciconia maguari*
	NXR25807.1	*Cinclus mexicanus*
	NXO20789.1	*Cisticola juncidis*
	NWW82590.1	*Climacteris rufus*
	NXB05615.1	*Cnemophilus loriae*
	NXE73871.1	*Cochlearius cochlearius*
	OXB78024.1	*Colinus virginianus*
	XP_010198933.1	*Colius striatus*
	XP_005504123.1	*Columba livia*
	NWQ76924.1	*Columbina picui*
	NXD37018.1	*Copsychus sechellarum*
	XP_027493193.1	*Corapipo altera*
	XP_008632943.1	*Corvus brachyrhynchos*
	NXD47704.1	*Corvus moneduloides*
	NXJ91645.1	*Corythaixoides concolor*

	XP_015723838.1	*Coturnix japonica*
	NWS69840.1	*Crotophaga sulcirostris*
	NWI20006.1	*Crypturellus soui*
	NWJ03121.1	*Crypturellus undulatus*
	XP_009553924.1	*Cuculus canorus*
	XP_023786694.1	*Cyanistes caeruleus*
	XP_035392834.1	*Cygnus atratus*
	NWV57530.1	*Daphoenositta chrysoptera*
	NWV78815.1	*Dasyornis broadbenti*
	NXH49295.1	*Dicaeum eximium*
	NXJ25364.1	*Dicrurus megarhynchus*
	NXB72502.1	*Donacobius atricapilla*
	NXG36977.1	*Dromaius novaehollandiae*
	NWU56459.1	*Dromas ardeola*
	NXU40100.1	*Drymodes brunneopygia*
	XP_009897380.1	*Dryobates pubescens*
	NWI72917.1	*Dryoscopus gambensis*
	NXH86703.1	*Edolisoma coerulescens*
	XP_009644325.1	*Egretta garzetta*
	NXD25015.1	*Elachura formosa*
	NWR18335.1	*Emberiza fucata*
	XP_027752516.1	*Empidonax traillii*
	NWY76443.1	*Erithacus rubecula*
	NXS77645.1	*Erpornis zantholeuca*
	NWT51400.1	*Erythrocercus mccallii*
	NXF96846.1	*Eubucco bourcierii*
	NXA32803.1	*Eudromia elegans*
	KAF1613385.1	*Eudyptes chrysolophus*
	KAF1585203.1	*Eudyptes robustus*
	KAF1548990.1	*Eudyptes schlegeli*
	KAF1498376.1	*Eudyptula novaehollandiae*
	NXB41073.1	*Eulacestoma nigropectus*
	XP_010150780.1	*Eurypyga helias*
	NXW64023.1	*Eurystomus gularis*
	XP_005442982.1	*Falco cherrug*
	XP_005237060.1	*Falco peregrinus*
	NWW20991.1	*Falcunculus frontatus*
	XP_005049151.1	*Ficedula albicollis*
	NXK94067.1	*Formicarius rufipectus*
	NWH48423.1	*Fregata magnificens*
	XP_009580620.1	*Fulmarus glacialis*
	NWR94728.1	*Furnarius figulus*
	NXI44921.1	*Galbula dea*
	XP_426573.2	*Gallus gallus*
	XP_009813285.1	*Gavia stellata*
	NWH63192.1	*Geococcyx californianus*
	XP_005415947.1	*Geospiza fortis*
	NXY71154.1	*Glareola pratincola*
	NXL29853.1	*Glaucidium brasilianum*
	NXG15199.1	*Grallaria varia*
	NWV41223.1	*Grantiella picta*
	NWH17218.1	*Grus americana*
	NXM37599.1	*Gymnorhina tibicen*
	NXD75789.1	*Halcyon senegalensis*
	XP_009912995.1	*Haliaeetus albicilla*
	NXP54238.1	*Heliornis fulica*
	NXG62016.1	*Hemiprocne comata*
	NXK09050.1	*Herpetotheres cachinnans*
	NXR55460.1	*Hippolais icterina*

	NXW75708.1	*Hirundo rustica*
	NWU40809.1	*Hylia prasina*
	NXR94402.1	*Hypocryptadius cinnamomeus*
	NXA22144.1	*Ibidorhyncha struthersii*
	NWW69016.1	*Ifrita kowaldi*
	NXM56359.1	*Illadopsis cleaveri*
	NXN17692.1	*Indicator maculatus*
	NXI20327.1	*Irena cyanogastra*
	NXS97934.1	*Jacana jacana*
	KAG0128765.1	*Lamprotornis superbus*
	NWT78559.1	*Lanius ludovicianus*
	NXX06372.1	*Larus smithsonianus*
	NXP35692.1	*Leiothrix lutea*
	XP_017691037.1	*Lepidothrix coronata*
	NXL73432.1	*Leptocoma aspasia*
	XP_009957273.1	*Leptosomus discolor*
	NXB49206.1	*Leucopsar rothschildi*
	PKU47079.1	*Limosa lapponica baueri*
	NXO34244.1	*Locustella ochotensis*
	XP_021408102.1	*Lonchura striata domestica*
	NXE06189.1	*Lophotis ruficrista*
	NWZ01414.1	*Loxia curvirostra*
	NWV89259.1	*Machaerirhynchus nigripectus*
	NWV61304.1	*Malurus elegans*
	XP_017927185.1	*Manacus vitellinus*
	KAF1506214.1	*Megadyptes antipodes antipodes*
	NXA87663.1	*Melanocharis versteri*
	XP_019472642.1	*Meleagris gallopavo*
	XP_005152626.1	*Melopsittacus undulatus*
	NWQ49044.1	*Melospiza melodia*
	KAF2987511.1	*Melospiza melodia maxima*
	NXE91554.1	*Menura novaehollandiae*
	XP_008945516.1	*Merops nubicus*
	NXK99089.1	*Mesembrinibis cayennensis*
	XP_010178165.1	*Mesitornis unicolor*
	NWS93920.1	*Mionectes macconnelli*
	NXA66458.1	*Mohoua ochrocephala*
	XP_036242196.1	*Molothrus ater*
	NWR98737.1	*Motacilla alba*
	XP_037998770.1	*Motacilla alba alba*
	NXH26574.1	*Myiagra hebetior*
	NXS18194.1	*Mystacornis crossleyi*
	XP_027534255.1	*Neopelma chrysocephalum*
	NWQ72057.1	*Neopipo cinnamomea*
	NWZ96000.1	*Nesospiza acunhae*
	XP_010021626.1	*Nestor notabilis*
	NXX24172.1	*Nicator chloris*
	XP_009460604.1	*Nipponia nippon*
	NXA48827.1	*Nothocercus julius*
	NXD10797.1	*Nothocercus nigrocapillus*
	NWX83018.1	*Nothoprocta pentlandii*
	XP_025907871.1	*Nothoprocta perdicaria*
	NWX27445.1	*Notiomystis cincta*
	XP_021255407.1	*Numida meleagris*
	NXF34696.1	*Nyctibius bracteatus*
	NXQ83449.1	*Nyctibius grandis*
	NXN24959.1	*Nycticryphes semicollaris*
	NXW44842.1	*Nyctiprogne leucopyga*
	NXF50856.1	*Oceanites oceanicus*

	NXH78617.1	*Oceanodroma tethys*
	NWU75606.1	*Onychorhynchus coronatus*
	XP_009932320.1	*Opisthocomus hoazin*
	NWW01058.1	*Oreocharis arfaki*
	NXU70333.1	*Oreotrochilus melanogaster*
	NWV25296.1	*Origma solitaria*
	NXO15746.1	*Oriolus oriolus*
	NXB99580.1	*Orthonyx spaldingii*
	NXM28672.1	*Oxyruncus cristatus*
	XP_035187318.1	*Oxyura jamaicensis*
	NXH99692.1	*Pachycephala philippinensis*
	NWS14052.1	*Pachyramphus minor*
	NXS70306.1	*Pandion haliaetus*
	NWW41051.1	*Panurus biarmicus*
	NXU08060.1	*Pardalotus punctatus*
	XP_015491030.1	*Parus major*
	NXP89187.1	*Passerina amoena*
	OPJ80113.1	*Patagioenas fasciata monilis*
	NWW49886.1	*Pedionomus torquatus*
	NXT36159.1	*Pelecanoides urinatrix*
	XP_009491858.1	*Pelecanus crispus*
	NXC40379.1	*Penelope pileata*
	NXQ21310.1	*Peucedramus taeniatus*
	XP_010285122.1	*Phaethon lepturus*
	NXW28731.1	*Phaetusa simplex*
	NXO73510.1	*Phainopepla nitens*
	XP_009498492.1	*Phalacrocorax carbo*
	XP_031456993.1	*Phasianus colchicus*
	NWY26211.1	*Pheucticus melanocephalus*
	NWH77420.1	*Piaya cayana*
	NWI40770.1	*Picathartes gymnocephalus*
	XP_027587289.1	*Pipra filicauda*
	NXK29999.1	*Piprites chloris*
	NWI91341.1	*Pitta sordida*
	NWU21086.1	*Platysteira castanea*
	NXM14334.1	*Ploceus nigricollis*
	NXT55396.1	*Pluvianellus socialis*
	NXX23865.1	*Podargus strigoides*
	NXL50176.1	*Podilymbus podiceps*
	NWZ77490.1	*Poecile atricapillus*
	NWS31006.1	*Polioptila caerulea*
	NXY31913.1	*Pomatorhinus ruficollis*
	NXS34784.1	*Pomatostomus ruficeps*
	NWX62176.1	*Promerops cafer*
	NXT04933.1	*Prunella fulvescens*
	NWT65553.1	*Prunella himalayana*
	XP_005519906.1	*Pseudopodoces humilis*
	NXG41566.1	*Psilopogon haemacephalus*
	NXJ00979.1	*Psophia crepitans*
	NWU66835.1	*Pterocles burchelli*
	XP_010079514.1	*Pterocles gutturalis*
	NXY09753.1	*Pteruthius melanotis*
	NWV07481.1	*Ptilonorhynchus violaceus*
	NXE32828.1	*Ptilorrhoa leucosticta*
	NXR76930.1	*Pycnonotus jocosus*
	XP_009330652.1	*Pygoscelis adeliae*
	KAF1671373.1	*Pygoscelis papua*
	NXQ65629.1	*Quiscalus mexicanus*
	NXP77852.1	*Ramphastos sulfuratus*

	NWR52593.1	*Regulus satrapa*
	NXH54621.1	*Rhabdornis inornatus*
	NXR67183.1	*Rhadina sibilatrix*
	NXB26226.1	*Rhagologus leucostigma*
	NWY88292.1	*Rhegmatorhina hoffmannsi*
	NXN99868.1	*Rhinopomastus cyanomelas*
	NXN35863.1	*Rhinoptilus africanus*
	NXI83036.1	*Rhipidura dahli*
	NWW91075.1	*Rhynochetos jubatus*
	NXJ59699.1	*Rostratula benghalensis*
	NXR00020.1	*Sagittarius serpentarius*
	NXG06998.1	*Sakesphorus luctuosus*
	NXA14089.1	*Sapayoa aenigma*
	NXF73936.1	*Sclerurus mexicanus*
	NXX55761.1	*Scopus umbretta*
	NXP27065.1	*Scytalopus superciliaris*
	NXM72021.1	*Serilophus lunatus*
	XP_009086279.1	*Serinus canaria*
	NWQ99908.1	*Sinosuthora webbiana*
	NXO75284.1	*Sitta europaea*
	NXF12597.1	*Smithornis capensis*
	KAF1428768.1	*Spheniscus magellanicus*
	KAF1399644.1	*Spheniscus mendiculus*
	NXJ46460.1	*Spizaetus tyrannus*
	NXX69544.1	*Spizella passerina*
	NWX52316.1	*Steatornis caripensis*
	NXG82266.1	*Stercorarius parasiticus*
	NXI25234.1	*Sterrhoptilus dennistouni*
	XP_030341457.1	*Strigops habroptila*
	NXB61160.1	*Struthidea cinerea*
	XP_009674596.1	*Struthio camelus australis*
	XP_014745550.1	*Sturnus vulgaris*
	NWY48204.1	*Sylvia atricapilla*
	NXM99811.1	*Sylvia borin*
	NXK58304.1	*Sylvietta virens*
	NXT28764.1	*Syrrhaptes paradoxus*
	NWR28642.1	*Tachuris rubrigastra*
	XP_002199531.1	*Taeniopygia guttata*
	XP_009985399.1	*Tauraco erythrolophus*
	NXP08017.1	*Thinocorus orbignyianus*
	NXA79682.1	*Thryothorus ludovicianus*
	NWI04416.1	*Tichodroma muraria*
	XP_010217678.1	*Tinamus guttatus*
	NWS79361.1	*Toxostoma redivivum*
	NXJ79361.1	*Trogon melanurus*
	NXU49014.1	*Turnix velox*
	NXM00709.1	*Tyrannus savana*
	XP_009964462.2	*Tyto alba alba*
	NWU92803.1	*Upupa epops*
	NXV41897.1	*Uria aalge*
	NXX84617.1	*Urocolius indicus*
	NWT96562.1	*Urocynchramus pylzowi*
	NXB96432.1	*Vidua chalybeata*
	NXQ04014.1	*Vidua macroura*
	NWT18044.1	*Vireo altiloquus*
	NXT84156.1	*Zapornia atra*
	XP_005484170.1	*Zonotrichia albicollis*
Mammalia GSDME	XP_026930937.1	*Acinonyx jubatus*
	XP_034525218.1	*Ailuropoda melanoleuca*

	XP_021525832.1	*Aotus nancymaae*
	XP_037002709.1	*Artibeus jamaicensis*
	XP_034374699.1	*Arvicanthis niloticus*
	XP_038174893.1	*Arvicola amphibius*
	XP_007171559.1	*Balaenoptera acutorostrata scammoni*
	XP_036720084.1	*Balaenoptera musculus*
	KAB0390338.1	*Balaenoptera physalus*
	XP_010853670.1	*Bison bison bison*
	XP_019824428.1	*Bos indicus*
	XP_005902937.1	*Bos mutus*
	NP_001180041.1	*Bos taurus*
	XP_006055333.1	*Bubalus bubalis*
	XP_002751525.1	*Callithrix jacchus*
	XP_025729082.1	*Callorhinus ursinus*
	XP_010966934.1	*Camelus bactrianus*
	EPY87717.1	*Camelus ferus*
	XP_025320188.1	*Canis lupus dingo*
	XP_038292060.1	*Canis lupus familiaris*
	ALN66867.1	*Capra hircus*
	XP_008070518.1	*Carlito syrichta*
	XP_020034992.1	*Castor canadensis*
	XP_005002891.1	*Cavia porcellus*
	XP_017382492.1	*Cebus imitator*
	XP_004418969.1	*Ceratotherium simum simum*
	XP_011937023.1	*Cercocebus atys*
	OWK06618.1	*Cervus elaphus hippelaphus*
	XP_013366053.1	*Chinchilla lanigera*
	XP_007980046.2	*Chlorocebus sabaeus*
	XP_037692845.1	*Choloepus didactylus*
	XP_006832344.1	*Chrysochloris asiatica*
	XP_011793943.1	*Colobus angolensis palliatus*
	XP_012576204.1	*Condylura cristata*
	XP_027272248.1	*Cricetulus griseus*
	KAF0875838.1	*Crocuta crocuta*
	XP_004447610.1	*Dasypus novemcinctus*
	XP_022440687.1	*Delphinapterus leucas*
	XP_024418892.1	*Desmodus rotundus*
	KAF5927743.1	*Diceros bicornis minor*
	XP_012879848.1	*Dipodomys ordii*
	XP_004702903.1	*Echinops telfairi*
	XP_006902459.1	*Elephantulus edwardii*
	XP_022359062.1	*Enhydra lutris kenyoni*
	XP_008146232.1	*Eptesicus fuscus*
	XP_014694495.1	*Equus asinus*
	NP_001075358.1	*Equus caballus*
	XP_007529121.1	*Erinaceus europaeus*
	XP_027975160.1	*Eumetopias jubatus*
	XP_003982936.1	*Felis catus*
	XP_010626527.1	*Fukomys damarensis*
	XP_008591692.1	*Galeopterus variegatus*
	XP_030713228.1	*Globicephala melas*
	XP_004045237.1	*Gorilla gorilla gorilla*
	XP_028645887.1	*Grammomys surdaster*
	VCX43294.1	*Gulo gulo*
	XP_035953098.1	*Halichoerus grypus*
	XP_021109770.1	*Heterocephalus glaber*
	XP_019507556.1	*Hipposideros armiger*
	NP_004394.1	*Homo sapiens*
	XP_032615033.1	*Hylobates moloch*

	XP_021577894.1	*Ictidomys tridecemlineatus*
	XP_004652861.1	*Jaculus jaculus*
	XP_026958176.1	*Lagenorhynchus obliquidens*
	XP_006729473.2	*Leptonychotes weddellii*
	XP_030877431.1	*Leptonychotes weddellii*
	XP_007466126.1	*Lipotes vexillifer*
	XP_032728902.1	*Lontra canadensis*
	XP_023400115.1	*Loxodonta africana*
	XP_030163969.1	*Lynx canadensis*
	VFV47865.1	*Lynx pardinus*
	XP_005550019.1	*Macaca fascicularis*
	AFE76967.1	*Macaca mulatta*
	XP_011729489.1	*Macaca nemestrina*
	XP_011825996.1	*Mandrillus leucophaeus*
	XP_036846446.1	*Manis javanica*
	XP_036733825.1	*Manis pentadactyla*
	XP_027788502.1	*Marmota flaviventris*
	XP_015345627.1	*Marmota marmota marmota*
	VTJ58429.1	*Marmota monax*
	XP_031237728.1	*Mastomys coucha*
	XP_021486754.1	*Meriones unguiculatus*
	XP_005087240.1	*Mesocricetus auratus*
	XP_012628783.1	*Microcebus murinus*
	XP_005366675.2	*Microtus ochrogaster*
	XP_016072537.1	*Miniopterus natalensis*
	XP_034876651.1	*Mirounga leonina*
	XP_036102909.1	*Molossus molossus*
	XP_001368943.1	*Monodelphis domestica*
	XP_029089784.1	*Monodon monoceros*
	KAB0344585.1	*Muntiacus muntjak*
	KAB0375691.1	*Muntiacus reevesi*
	XP_021020548.1	*Mus caroli*
	AAI32304.1	*Mus musculus*
	XP_021046698.1	*Mus pahari*
	XP_032160672.1	*Mustela erminea*
	XP_004743370.1	*Mustela putorius furo*
	XP_005872327.1	*Myotis brandtii*
	XP_006779071.1	*Myotis davidii*
	XP_006088855.1	*Myotis lucifugus*
	XP_036183766.1	*Myotis myotis*
	XP_008839709.1	*Nannospalax galili*
	XP_021545343.1	*Neomonachus schauinslandi*
	XP_024610804.1	*Neophocaena asiaeorientalis asiaeorientalis*
	OBS73827.1	*Neotoma lepida*
	XP_003270463.1	*Nomascus leucogenys*
	XP_004582613.1	*Ochotona princeps*
	XP_004626528.1	*Octodon degus*
	XP_004397435.1	*Odobenus rosmarus divergens*
	XP_020733679.1	*Odocoileus virginianus texanus*
	XP_036036139.1	*Onychomys torridus*
	XP_004265687.1	*Orcinus orca*
	XP_028926175.1	*Ornithorhynchus anatinus*
	XP_007944697.1	*Orycteropus afer afer*
	XP_002713872.1	*Oryctolagus cuniculus*
	XP_003788653.1	*Otolemur garnettii*
	XP_014950834.1	*Ovis aries*
	XP_003807975.1	*Pan paniscus*
	XP_001159685.3	*Pan troglodytes*
	XP_007089104.1	*Panthera tigris altaica*

	XP_003896237.1	*Papio anubis*
	XP_028748470.1	*Peromyscus leucopus*
	XP_006982246.1	*Peromyscus maniculatus bairdii*
	XP_020849085.1	*Phascolarctos cinereus*
	XP_032257813.1	*Phoca vitulina*
	XP_032498637.1	*Phocoena sinus*
	XP_028381748.1	*Phyllostomus discolor*
	XP_023987154.2	*Physeter catodon*
	XP_023081707.1	*Piliocolobus tephrosceles*
	XP_036309606.1	*Pipistrellus kuhlii*
	XP_024105635.1	*Pongo abelii*
	XP_012496187.1	*Propithecus coquereli*
	XP_006912063.1	*Pteropus alecto*
	XP_023387299.1	*Pteropus vampyrus*
	XP_025785639.1	*Puma concolor*
	NP_001178678.1	*Rattus norvegicus*
	XP_032762429.1	*Rattus rattus*
	KAF6301714.1	*Rhinolophus ferrumequinum*
	XP_017719434.1	*Rhinopithecus bieti*
	XP_010374230.1	*Rhinopithecus roxellana*
	XP_016015046.2	*Rousettus aegyptiacus*
	XP_003935184.1	*Saimiri boliviensis boliviensis*
	XP_032142870.1	*Sapajus apella*
	XP_031796450.1	*Sarcophilus harrisii*
	XP_004604319.1	*Sorex araneus*
	TEA22943.1	*Sousa chinensis*
	XP_029786181.1	*Suricata suricatta*
	XP_003134903.2	*Sus scrofa*
	XP_038616349.1	*Tachyglossus aculeatus*
	XP_037365241.1	*Talpa occidentalis*
	XP_033048935.1	*Trachypithecus francoisi*
	XP_004377520.1	*Trichechus manatus latirostris*
	XP_014448712.2	*Tupaia chinensis*
	XP_033718980.1	*Tursiops truncatus*
	XP_026256023.1	*Urocitellus parryii*
	XP_026363606.1	*Ursus arctos horribilis*
	XP_008684626.1	*Ursus maritimus*
	XP_015092757.1	*Vicugna pacos*
	XP_027728241.1	*Vombatus ursinus*
	XP_025848911.1	*Vulpes vulpes*
	XP_027429302.2	*Zalophus californianus*
Mammalia GSDMF	XP_014942207.1	*Acinonyx jubatus*
	XP_002918869.4	*Ailuropoda melanoleuca*
	XP_012326893.1	*Aotus nancymaae*
	XP_036985941.1	*Artibeus jamaicensis*
	XP_034351820.1	*Arvicanthis niloticus*
	XP_038193264.1	*Arvicola amphibius*
	XP_028024398.1	*Balaenoptera acutorostrata scammoni*
	XP_036714871.1	*Balaenoptera musculus*
	KAB0395846.1	*Balaenoptera physalus*
	XP_019825658.1	*Bos indicus*
	XP_027412455.1	*Bos indicus x Bos taurus*
	MXQ91385.1	*Bos mutus*
	NP_001180112.1	*Bos taurus*
	XP_006059644.1	*Bubalus bubalis*
	XP_035160279.1	*Callithrix jacchus*
	XP_025743405.1	*Callorhinus ursinus*
	XP_006194628.1	*Camelus ferus*
	XP_025316114.1	*Canis lupus dingo*

	XP_022270525.1	*Canis lupus familiaris*
	ALN66868.1	*Capra hircus*
	XP_008053529.1	*Carlito syrichta*
	XP_020033548.1	*Castor canadensis*
	XP_003478567.1	*Cavia porcellus*
	XP_017388169.1	*Cebus imitator*
	XP_004426736.1	*Ceratotherium simum simum*
	OWJ99816.1	*Cervus elaphus hippelaphus*
	KAF4026341.1	*Cervus hanglu yarkandensis*
	XP_005373436.1	*Chinchilla lanigera*
	XP_037857819.1	*Chlorocebus sabaeus*
	XP_037705241.1	*Choloepus didactylus*
	XP_006866750.1	*Chrysochloris asiatica*
	XP_011799435.1	*Colobus angolensis palliatus*
	XP_004674538.1	*Condylura cristata*
	ERE71929.1	*Cricetulus griseus*
	KAF0875374.1	*Crocuta crocuta*
	XP_004476912.1	*Dasypus novemcinctus*
	XP_022425842.1	*Delphinapterus leucas*
	XP_024417352.1	*Desmodus rotundus*
	KAF5910680.1	*Diceros bicornis minor*
	XP_012883962.1	*Dipodomys ordii*
	XP_004701516.1	*Echinops telfairi*
	AEX07715.1	*Eidolon helvum*
	XP_006878851.1	*Elephantulus edwardii*
	XP_022356789.1	*Enhydra lutris kenyoni*
	XP_008136734.1	*Eptesicus fuscus*
	XP_014689590.1	*Equus asinus*
	XP_001500909.3	*Equus caballus*
	XP_007534290.1	*Erinaceus europaeus*
	XP_010635074.1	*Fukomys damarensis*
	XP_008565263.1	*Galeopterus variegatus*
	XP_030864437.1	*Gorilla gorilla gorilla*
	XP_035955027.1	*Halichoerus grypus*
	XP_004868199.1	*Heterocephalus glaber*
	XP_019509576.1	*Hipposideros armiger*
	XP_016859710.1	*Homo sapiens*
	XP_032611167.1	*Hylobates moloch*
	XP_005324712.1	*Ictidomys tridecemlineatus*
	XP_004660349.1	*Jaculus jaculus*
	XP_006733299.1	*Leptonychotes weddellii*
	XP_007450786.1	*Lipotes vexillifer*
	XP_032712096.1	*Lontra canadensis*
	XP_003406242.2	*Loxodonta africana*
	XP_015287897.1	*Macaca fascicularis*
	XP_028686721.1	*Macaca mulatta*
	XP_011717757.1	*Macaca nemestrina*
	XP_011847214.1	*Mandrillus leucophaeus*
	XP_017517118.1	*Manis javanica*
	XP_036735358.1	*Manis pentadactyla*
	XP_027801937.1	*Marmota flaviventris*
	XP_015336589.1	*Marmota marmota marmota*
	XP_021505179.1	*Meriones unguiculatus*
	XP_005065182.1	*Mesocricetus auratus*
	XP_012641314.1	*Microcebus murinus*
	XP_005346676.1	*Microtus ochrogaster*
	XP_016065696.1	*Miniopterus natalensis*
	XP_034862518.1	*Mirounga leonina*
	XP_036110605.1	*Molossus molossus*

	XP_001368857.1	*Monodelphis domestica*
	TKC39667.1	*Monodon monoceros*
	KAB0356577.1	*Muntiacus muntjak*
	KAB0379441.1	*Muntiacus reevesi*
	XP_021012402.1	*Mus caroli*
	NP_001074180.1	*Mus musculus*
	XP_032211508.1	*Mustela erminea*
	XP_004769035.1	*Mustela putorius furo*
	XP_005857693.1	*Myotis brandtii*
	XP_023599455.1	*Myotis lucifugus*
	XP_036176235.1	*Myotis myotis*
	XP_008853561.1	*Nannospalax galili*
	XP_021558921.1	*Neomonachus schauinslandi*
	XP_024588595.1	*Neophocaena asiaeorientalis asiaeorientalis*
	OBS71892.1	*Neotoma lepida*
	XP_030658209.1	*Nomascus leucogenys*
	XP_004634773.1	*Octodon degus*
	XP_004403794.1	*Odobenus rosmarus divergens*
	XP_020738717.1	*Odocoileus virginianus texanus*
	XP_036042054.1	*Onychomys torridus*
	XP_028928169.1	*Ornithorhynchus anatinus*
	XP_007935422.1	*Orycteropus afer afer*
	XP_002712360.1	*Oryctolagus cuniculus*
	XP_012661631.2	*Otolemur garnettii*
	XP_004004594.1	*Ovis aries*
	XP_008966741.2	*Pan paniscus*
	XP_009442090.1	*Pan troglodytes*
	XP_019316325.1	*Panthera pardus*
	XP_007083963.1	*Panthera tigris altaica*
	XP_031507246.1	*Papio anubis*
	XP_015843879.1	*Peromyscus maniculatus bairdii*
	XP_020846715.1	*Phascolarctos cinereus*
	XP_032275467.1	*Phoca vitulina*
	XP_032494333.1	*Phocoena sinus*
	XP_028365357.1	*Phyllostomus discolor*
	XP_007130807.2	*Physeter catodon*
	XP_023068106.1	*Piliocolobus tephrosceles*
	XP_036276665.1	*Pipistrellus kuhlii*
	PNJ60097.1	*Pongo abelii*
	XP_012506180.1	*Propithecus coquereli*
	AEX07714.1	*Pteronotus parnellii*
	XP_006921303.1	*Pteropus alecto*
	XP_023384880.1	*Pteropus vampyrus*
	XP_038962363.1	*Rattus norvegicus*
	XP_032757859.1	*Rattus rattus*
	AEX07713.1	*Rhinolophus ferrumequinum*
	XP_017723774.1	*Rhinopithecus bieti*
	XP_030772353.1	*Rhinopithecus roxellana*
	XP_016008060.1	*Rousettus aegyptiacus*
	XP_003921870.1	*Saimiri boliviensis boliviensis*
	XP_032148968.1	*Sapajus apella*
	XP_003764058.1	*Sarcophilus harrisii*
	XP_004601427.1	*Sorex araneus*
	TEA23281.1	*Sousa chinensis*
	XP_036900263.1	*Sturnira hondurensis*
	XP_029790180.1	*Suricata suricatta*
	XP_003133557.4	*Sus scrofa*
	XP_038606935.1	*Tachyglossus aculeatus*
	XP_037379522.1	*Talpa occidentalis*

	XP_025260079.1	*Theropithecus gelada*
	XP_033086897.1	*Trachypithecus francoisi*
	XP_004375524.1	*Trichechus manatus latirostris*
	XP_036601720.1	*Trichosurus vulpecula*
	XP_006151718.1	*Tupaia chinensis*
	XP_004311474.1	*Tursiops truncatus*
	XP_026247819.1	*Urocitellus parryii*
	XP_026348228.1	*Ursus arctos horribilis*
	XP_008685415.1	*Ursus maritimus*
	XP_006210278.1	*Vicugna pacos*
	XP_027722001.1	*Vombatus ursinus*
	XP_027447016.1	*Zalophus californianus*

1. Predicted from genome SCFE01000000
2. Collected from Notospermus geniculatus ver. 2.0

Table S2. The repetitive element landscape in *P. formosa* scaffold 1076.

SW	perc	perc	perc	query	Position in query				matching	repeat	Position in repeat		
score	div.	del.	ins.	sequence	begin	end	(left)		repeat	class/family	begin	end	(left)
70	0	0	0	NW_006801015.1	2484	2545	-31277	+	(CTGA)n	Simple_repeat	1	62	0
848	9.7	2.4	0	NW_006801015.1	3781	3904	-29918	+	rnd-1_family-458	Unknown	1	127	-169
11	9.7	8.6	2.7	NW_006801015.1	4064	4098	-29724	+	(AAGTAAT)n	Simple_repeat	1	37	0
2829	6.9	18.1	0	NW_006801015.1	4435	4898	-28924	+	rnd-4_family-1732	DNA/TcMar-Tigger	3	550	-532
823	12.4	11.3	4.2	NW_006801015.1	4898	5074	-28748	+	rnd-5_family-6937	Unknown	3	191	-234
272	22.2	14.8	2.5	NW_006801015.1	5525	5741	-28081	+	rnd-6_family-1973	DNA/CMC-EnSpm	757	999	-545
586	8.3	6	6.9	NW_006801015.1	5732	5846	-27976	+	rnd-5_family-6937	Unknown	184	297	-128
557	11.4	1.9	8.1	NW_006801015.1	5847	5951	-27871	C	rnd-1_family-76	DNA/hAT-Charlie	0	159	61
23	5.6	0	5.1	NW_006801015.1	5961	6001	-27821	+	(TTTTATC)n	Simple_repeat	1	39	0
353	16.4	0	0	NW_006801015.1	6014	6074	-27748	+	rnd-6_family-2308	DNA/hAT-Charlie	114	174	-27
805	3.8	7.5	0	NW_006801015.1	6067	6172	-27650	C	rnd-5_family-2464	Unknown	-22	115	2
2338	7.2	1.3	0	NW_006801015.1	6188	6494	-27328	+	rnd-1_family-122	DNA/PIF-ISL2EU	1	311	0
383	8	20.7	0.7	NW_006801015.1	6495	6525	-27297	+	rnd-2_family-85	LTR/Ngaro	287	333	-53
2306	6.2	3.3	0	NW_006801015.1	6547	6851	-26971	C	rnd-5_family-1226	DNA/PIF-ISL2EU	-15	769	455
48	0	0	0	NW_006801015.1	6960	7000	-26822	+	(AC)n	Simple_repeat	1	41	0
12	7.8	3.2	6.7	NW_006801015.1	7685	7715	-26107	+	A-rich	Low_complexity	1	30	0
13	17.1	2.3	4.8	NW_006801015.1	7779	7821	-26001	+	(CATTT)n	Simple_repeat	1	42	0
98	8.2	3.2	0	NW_006801015.1	8420	8576	-25246	+	(ACCGGG)n	Simple_repeat	1	162	0
12	5.5	0	0	NW_006801015.1	8692	8710	-25112	+	(ATG)n	Simple_repeat	1	19	0
270	7.7	5.1	0	NW_006801015.1	8863	8901	-24921	+	rnd-1_family-36	LINE/L2	47	87	-466
724	15.7	9	0.3	NW_006801015.1	9005	9193	-24629	+	rnd-1_family-36	LINE/L2	39	181	-372
518	14.7	19.7	2.7	NW_006801015.1	9339	9526	-24296	+	rnd-1_family-16	DNA/hAT-Charlie	2	185	-4
256	28.6	17.4	0.6	NW_006801015.1	9792	9946	-23876	C	rnd-6_family-1729	LINE/L2	-384	1629	1449
283	19.2	0	0	NW_006801015.1	10003	10054	-23768	+	rnd-6_family-14096	Unknown	507	558	-10
277	15.5	6.9	0	NW_006801015.1	10030	10087	-23735	+	rnd-6_family-14096	Unknown	497	558	-10
277	15.5	6.9	0	NW_006801015.1	10063	10120	-23702	+	rnd-6_family-14096	Unknown	497	558	-10
299	15.6	6.2	0	NW_006801015.1	10096	10159	-23663	+	rnd-6_family-14096	Unknown	497	564	-4
307	17.9	4.9	4.9	NW_006801015.1	10183	10264	-23558	C	rnd-6_family-3217	LINE/L2	-123	299	218
406	7.4	0	0.4	NW_006801015.1	10386	10441	-23381	C	rnd-1_family-356	LINE/Rex-Babar	-10	96	6
49	0	0	0	NW_006801015.1	11023	11066	-22756	+	(TGA)n	Simple_repeat	1	44	0
1117	11.3	10.1	0.3	NW_006801015.1	15007	15246	-18576	+	rnd-1_family-1	DNA/hAT-Charlie	4	229	0
440	18.2	0	0	NW_006801015.1	16982	17058	-16764	C	rnd-5_family-737	Unknown	-478	85	9
226	14.5	16.1	1.6	NW_006801015.1	17183	17238	-16584	C	rnd-4_family-783	DNA/hAT-Charlie	-568	344	281
449	19.4	8.6	4.1	NW_006801015.1	17290	17429	-16393	C	rnd-6_family-3330	LINE/Rex-Babar	-19	1055	910
430	22	3	1	NW_006801015.1	17429	17529	-16293	C	rnd-4_family-783	DNA/hAT-Charlie	-781	131	29
407	13	5.8	0	NW_006801015.1	17750	17818	-16004	C	rnd-1_family-356	LINE/Rex-Babar	-30	76	4
485	10.8	0	12.9	NW_006801015.1	23612	23707	-10115	+	rnd-1_family-391	Unknown	168	252	-460
1510	11.9	3.7	8.1	NW_006801015.1	23739	24037	-9785	+	rnd-1_family-530	Unknown	48	328	-2
247	14.3	4.1	0	NW_006801015.1	24106	24154	-9668	+	rnd-1_family-553	Unknown	10	60	-125
378	18.6	15.7	0	NW_006801015.1	24182	24282	-9540	+	rnd-5_family-302	SINE/tRNA	48	164	-11
1536	11.9	0.4	0.9	NW_006801015.1	24283	24528	-9294	C	rnd-1_family-14	DNA/hAT-Charlie	0	214	1
84	10.7	0	0	NW_006801015.1	26215	26347	-7475	+	(CCATCTGTCT)n	Simple_repeat	1	133	0
3128	8.1	4.3	1.9	NW_006801015.1	26963	27468	-6354	C	rnd-5_family-853	LINE/L2	-53	651	134
529	11.3	13	0	NW_006801015.1	27485	27599	-6223	+	rnd-4_family-283	Unknown	38	167	-294
254	14.4	14.3	8.3	NW_006801015.1	27609	27699	-6123	C	rnd-6_family-2161	Unknown	-306	112	17
1990	6.6	10	1.1	NW_006801015.1	27717	28066	-5756	+	rnd-5_family-853	LINE/L2	322	702	-2
282	29	3.8	1.5	NW_006801015.1	28842	28974	-4848	+	rnd-6_family-220	LINE/L1	653	788	-1870
2900	9.7	14.6	2.6	NW_006801015.1	29347	29929	-3893	C	rnd-6_family-6853	DNA/hAT-Charlie	0	1465	815
879	12.3	20.6	7.5	NW_006801015.1	29955	30309	-3513	C	rnd-6_family-6853	DNA/hAT-Charlie	-724	741	340
308	18.8	2	7.5	NW_006801015.1	30228	30325	-3497	+	rnd-6_family-6853	DNA/hAT-Charlie	815	907	-558
315	0	0	0	NW_006801015.1	30309	30331	-3491	C	rnd-6_family-953	Unknown	0	34	12
859	12.9	4.2	1.4	NW_006801015.1	30332	30473	-3349	C	rnd-1_family-59	DNA/hAT-Charlie	0	155	10
1914	4.8	0.4	0	NW_006801015.1	31474	31701	-2121	C	rnd-6_family-1729	LINE/L2	-959	1054	826
4558	5.5	1.3	0.5	NW_006801015.1	31700	32826	-996	C	rnd-6_family-1729	LINE/L2	-1396	617	1
460	13.7	3.1	3.9	NW_006801015.1	32825	32953	-869	+	rnd-4_family-969	Unknown	465	592	-68

Table S3. The credits for the pictures used in the manuscript.

Animal Phylum	Figure Origin
Placozoa	Mali o Kodis photograph
Ctenophora	Noah Schlottman
Cnidaria	Christoph Schomburg
Platyhelminthes	Cristina Guijarro
Priapulida	Michelle Site
Nematoda	Birgit Lang
Arthropoda	Fernando Campos De Domenico
Tardigrada	Michelle Site
Onychophora	Mali o Kodis photograph by Bruno Vellutini.
Bryozoa	Mali o Kodis image from the Proceedings of the Zoological Society of London
Entoprocta	Mali o Kodis image from the Biodiversity Heritage Library
Mollusca	Scott Hartman
Annelida	Michelle Site
Nemertea	Scott Hartman
Xenacoelomorpha	Andreas Hejnol
Echinodermata	Hans Hillewaert photo and T Michael Keesey vectorization
Chaetognatha	Michelle Site
Hemichordata	Ludwik G siorowski
Homo sapiens	NASA
Avian-1	Ferran Sayol
Avian-2	Steven-Traver
Reptilia-1	Gabriela Palomo-Munoz
Reptilia-2	Sarah-Werning
Amphibia	Yusan Yang
Actinopterygii-1	Milton Tan
Actinopterygii-2	Ingo-Braasch

Table S4. The full length GSDME sequences from GSDMEc containing species.

	GSDMEa	GSDMEb	GSDMEc
1	XP_026203484.1	RXM37111.1	XP_033869450.1
2	XP_030597377.1	XP_026226820.1	XP_033913364.2
3	XP_026012230.1	XP_035270340.1	XP_026209852.1
4	XP_013866637.1	XP_035284961.1	XP_026210856.1
5	XP_029023078.1	XP_030606161.1	XP_035259671.1
6	KAF3707611.1	XP_026041600.1	XP_030598022.1
7	XP_015229640.1	XP_013864222.1	XP_030598774.1
8	XP_033492408.1	XP_028983881.1	XP_026030411.1
9	XP_036000882.1	XP_038125417.1	XP_026030414.1
10	PWA15389.1	XP_015259533.1	XP_013877960.1
11	XP_005928721.1	XP_033495484.1	XP_013887471.1
12	XP_017277831.1	XP_028674104.1	XP_029019774.1
13	XP_018522627.1	XP_012718094.2	KAF3701606.1
14	XP_026155402.1	PWA33483.1	KAF3701666.1
15	XP_004547887.2	XP_005934834.1	XP_038127901.1
16	XP_038581104.1	XP_017282053.1	XP_038127904.1
17	XP_035762494.1	XP_018544241.1	XP_038130670.1
18	XP_005453900.1	KAF7669433.1	XP_015246556.1
19	XP_028284766.1	XP_026149142.1	XP_015246561.1
20	XP_007562681.1	XP_004541527.2	XP_015255603.1
21	XP_014884464.1	XP_036395434.1	XP_015255605.1
22	XP_014838454.1	XP_036371947.1	XP_033488610.1
23	XP_008419845.1	XP_006801451.1	XP_028661605.1
24	XP_005728881.1	XP_031602092.1	XP_035995790.1
25	XP_030002330.1	XP_003454487.2	XP_035995791.1
26	XP_027891527.1	XP_028280814.1	XP_035995795.1
27	XP_032436028.1	XP_007570012.1	XP_005947650.1
28	XP_023200180.1	XP_014892308.1	XP_017288141.1
29		XP_014859811.1	XP_037835230.1
30		XP_017165221.1	XP_018518620.1
31		XP_005738539.1	XP_015200904.1
32		XP_030012894.1	XP_015217593.1
33		XP_027868605.1	XP_026152356.1
34		XP_032413990.1	XP_004561929.2
35		XP_023186332.1	XP_036392901.1
36			XP_038557995.1
37			XP_031596922.1
38			XP_019216956.1
39			XP_028273152.1
40			XP_028274043.1
41			XP_007544341.1
42			XP_014870251.1
43			XP_014870253.1
44			XP_014829804.1
45			XP_014829807.1
46			XP_014829970.1
47			XP_017158798.1
48			XP_008400852.2
49			XP_005748475.1
50			XP_005748491.1
51			XP_005748492.1
52			XP_029985328.1
53			XP_030016339.1
54			XP_027881410.1
55			XP_027881411.1
56			XP_032425120.1
57			XP_032425124.1

Table S5. The primers used for GSDME mutagenesis.

Primer	Sequence (5'-3')
GSDME-F2A forward	5'-TCAGATCTCGAGATGGCGGCCAAAGCAACC-3'
GSDME-F2A reverse	5'-CGCCATCTCGAGATCTGAGTCCGGTAGCGC-3'
GSDME-A5D forward	5'-GAGATGTTTGCCAAAGACACCAGGAATTTT-3'
GSDME-A5D reverse	5'-GTCTTTGGCAAACATCTCGAGATCTGAGTC-3'
GSDME-G17D forward	5'-GAAGTTGATGCTGATGACGACCTGATTGCA-3'
GSDME-G17D reverse	5'-GTCATCAGCATCAACTTCTCTAAGAAAATT-3'
GSDME-W44A forward	5'-AAAAAGAAGAGATTCGCGTGCTGGCAGAGA-3'
GSDME-W44A reverse	5'-CGCGAATCTCTTCTTTTTTGTCACCAGACT-3'
GSDME-Q47A forward	5'-AGATTCTGGTGCTGGGCGAGACCCAAGTAC-3'
GSDME-Q47A reverse	5'-CGCCCAGCACCAGAATCTCTTCTTTTTTGT-3'
GSDME-Y51A forward	5'-TGGCAGAGACCCAAGGCGCAGTTTTTATCC-3'
GSDME-Y51A reverse	5'-CGCCTTGGGTCTCTGCCAGCACCAGAATCT-3'
GSDME-P70A forward	5'-GACCAATTTCCGAGTGCGGTGGTCGTGGAG-3'
GSDME-P70A reverse	5'-CGCACTCGGAAATTGGTCTTCTATGAGTAC-3'
GSDME-E223R forward	5'-GCCTACGGTGTCATTCGCTTATACGTGAAA-3'
GSDME-E223R reverse	5'-GCGAATGACACCGTAGGCAATGGTGGTGGC-3'
GSDME-L327D forward	5'-GAACTACTCATGGTCGACGAACCAGTGTGC-3'
GSDME-L327D reverse	5'-GTCGACCATGAGTAGTTCATCATCAAATAG-3'
GSDME-L451D forward	5'-GGGATTGTGCAGCGCGACTTTGCCTCAGCT-3'
GSDME-L451D reverse	5'-GTCGCGCTGCACAATCCCAAACCTTTCTGT-3'
GSDME-G487D forward	5'-TGTATAACCCTGAATGACCTCTGTGCTTTA-3'
GSDME-G487D reverse	5'-GTCATTCAGGGTTATACAAAGAAGCAGTGG-3'
GSDME-L491D forward	5'-AATGGACTCTGTGCTGACGGCAGAGAACAT-3'
GSDME-L491D reverse	5'-GTCAGCACAGAGTCCATTCAGGGTTATACA-3'
GSDME forward	5'-CTACCGGACTCAGATCTCGAGATGTTTGCCAAAGCAACCAGGAATTTTC-3'
GSDME reverse	5'-CGACTGCAGAATTCGAAGCTTTGAATGTTCTCTGCCTAAAGCACAGAG-3'
GSDME-NT reverse	5'-CGACTGCAGAATTCGAAGCTTATCTGGCATGTCTATGAATGCAAACTC-3'
GSDME-F2A-NT forward	5'-CTACCGGACTCAGATCTCGAGATGGCGGCCAAAGCAACCAGGAATTTT-3'
GSDME-A5D-NT forward	5'-CTACCGGACTCAGATCTCGAGATGTTTGCCAAAGACACCAGGAATTTT-3'
